# Emerging cellular themes in leukodystrophies

**DOI:** 10.3389/fcell.2022.902261

**Published:** 2022-08-08

**Authors:** Joseph C. Nowacki, Ashley M. Fields, Meng Meng Fu

**Affiliations:** NINDS (National Institute of Neurological Disorders and Stroke), National Institutes of Health, Bethesda, MD, United States

**Keywords:** leukodystrophy, oligodendrocyte, myelin, white matter (WM), astrocyte, microglia, glia

## Abstract

Leukodystrophies are a broad spectrum of neurological disorders that are characterized primarily by deficiencies in myelin formation. Clinical manifestations of leukodystrophies usually appear during childhood and common symptoms include lack of motor coordination, difficulty with or loss of ambulation, issues with vision and/or hearing, cognitive decline, regression in speech skills, and even seizures. Many cases of leukodystrophy can be attributed to genetic mutations, but they have diverse inheritance patterns (e.g., autosomal recessive, autosomal dominant, or X-linked) and some arise from *de novo* mutations. In this review, we provide an updated overview of 35 types of leukodystrophies and focus on cellular mechanisms that may underlie these disorders. We find common themes in specialized functions in oligodendrocytes, which are specialized producers of membranes and myelin lipids. These mechanisms include myelin protein defects, lipid processing and peroxisome dysfunction, transcriptional and translational dysregulation, disruptions in cytoskeletal organization, and cell junction defects. In addition, non-cell-autonomous factors in astrocytes and microglia, such as autoimmune reactivity, and intercellular communication, may also play a role in leukodystrophy onset. We hope that highlighting these themes in cellular dysfunction in leukodystrophies may yield conceptual insights on future therapeutic approaches.

## Introduction

White matter development initiates *in utero* in the fetus, continues into late adolescence and adulthood, and culminates to constitute ∼40% of adult human brains. White matter contains both neuronal axons and myelin sheaths that wrap concentrically in many layers around axons like paper towels. White matter is so named, because myelin is lipid rich and has a white, fatty appearance. Myelin functions to insulate axons, to facilitate saltatory conduction, and to increase axon potential velocity. During human development, myelin production begins slowly during the third trimester of pregnancy, and rapidly accelerates postnatally and during early childhood (Barkovich et al., 1988; Jakovcevski et al., 2009; Tomassy, Dershowitz, and Arlotta 2016). By 2 years of age, the majority of myelination is complete, as visualized by MRI scans (magnetic resonance imaging) and postmortem silver staining (Flechsig 1920; Wolf et al., 1993). Although white matter volume continues to increase into adulthood, its rate of formation is significantly slower (Fields 2010; Groeschel et al., 2010).

Developmental white matter diseases generally fall under the category of leukodystrophies. The term leukodystrophy can be broken down into its Greek origins: “leuko” for white, “dys'' for bad or abnormal, and “trophy” for growth. Together, these root words define leukodystrophies as a broad spectrum of neurological disorders that are characterized primarily by deficiencies in myelin formation that are usually not secondary to neuronal defects. Incidence estimates range from one in 8,000 to lower estimates of one in 50,000 or one in 80,000 (Vanderver et al., 2012). Symptom onset typically occurs during childhood, when neural pathways and associated white matter tracts involved in speech, motor coordination, and memory are refined. Thus, common symptoms of leukodystrophies include regression or loss of developmental abilities, such as speech and walking, poor motor control, and cognitive defects. Though the majority of leukodystrophies present during childhood, some are adult-onset. This disparity in age of onset likely reflects diverse etiologies and disease mechanisms and, therefore, is an ongoing topic of research.

Though some cases of leukodystrophy have unclear etiology (see Box 1), many types have a genetic cause with diverse patterns of inheritance, including autosomal recessive, autosomal dominant, or X-linked recessive. These modes of inheritance dictate the appropriate types of therapeutic approaches that have been attempted in translational studies. Recessive leukodystrophies are often addressed through replacement therapies that aim to restore loss of function. These approaches include nutrient supplementation, enzymatic replacement via viruses, and transplantation of stem cells. For example, iPSCs (induced pluripotent stem cells) derived from human skin cells can be induced to become specialized cells, such as oligodendrocytes (Chanoumidou et al., 2020). Autosomal dominant leukodystrophies are often addressed through therapies that aim to suppress the mutant gene, mRNA, or protein. Traditional small molecule drug screens have been performed, typically on cellular models of leukodystrophy. In addition, a new class of drug called ASOs (antisense oligonucleotides) can bind to RNA targeting sequences to achieve gene silencing. ASOs for the treatment of SMA (spinal muscular atrophy) are FDA-approved and several ongoing ASO clinical trials target ALS (amyotrophic lateral sclerosis) and other genetic neurological diseases (Amado and Davidson 2021). Thus, multiple therapeutic approaches are available to address the diverse types of leukodystrophies.

BOX 1 **Non-genetic Factors**

Non-genetic factors, particularly neonatal white matter injury, can adversely affect neurodevelopment in long-lasting ways. Abnormal MRI findings in premature infants are very strong predictors of unfavorable neurodevelopmental outcomes, including cognitive delays, motor delays, and cerebral palsy (Woodward et al., 2006). Congenital heart disease is the most common major birth defect, and can place infants at risk of hypoxia (Marelli et al., 2007). Hypoxic insult causes oligodendrocyte death and delayed oligodendrocyte differentiation, thus leading to abnormalities in developmental myelination (Back et al., 2001; Jablonska et al., 2012). Interestingly, hypoxic newborn brains may share some common molecular markers with adult multiple sclerosis (MS) brains. Indeed, the remyelination regulator *Axin2* is present in white matter lesions that are found in both human newborn brains with hypoxic damage and active MS lesions in adults (Fancy et al., 2011).

Pathogens and the immune responses that they instigate can also have effects on white matter development. Perinatal inflammatory insult subsequent to maternal infection is associated with cerebral palsy as well as low scores on a number of development indexes (Stoll et al., 2004). For example, *E. coli* infection reduces the expression of oligodendrocyte-differentiation promoting transcripts and leads to myelin loss during a critical period of peak myelination (Favrais et al., 2011; Lieblein-Boff et al., 2013). Thus, the etiology of leukodystrophies are diverse and genetic causes are not the only consideration in white matter development and disease.

**FIGURE 1:**
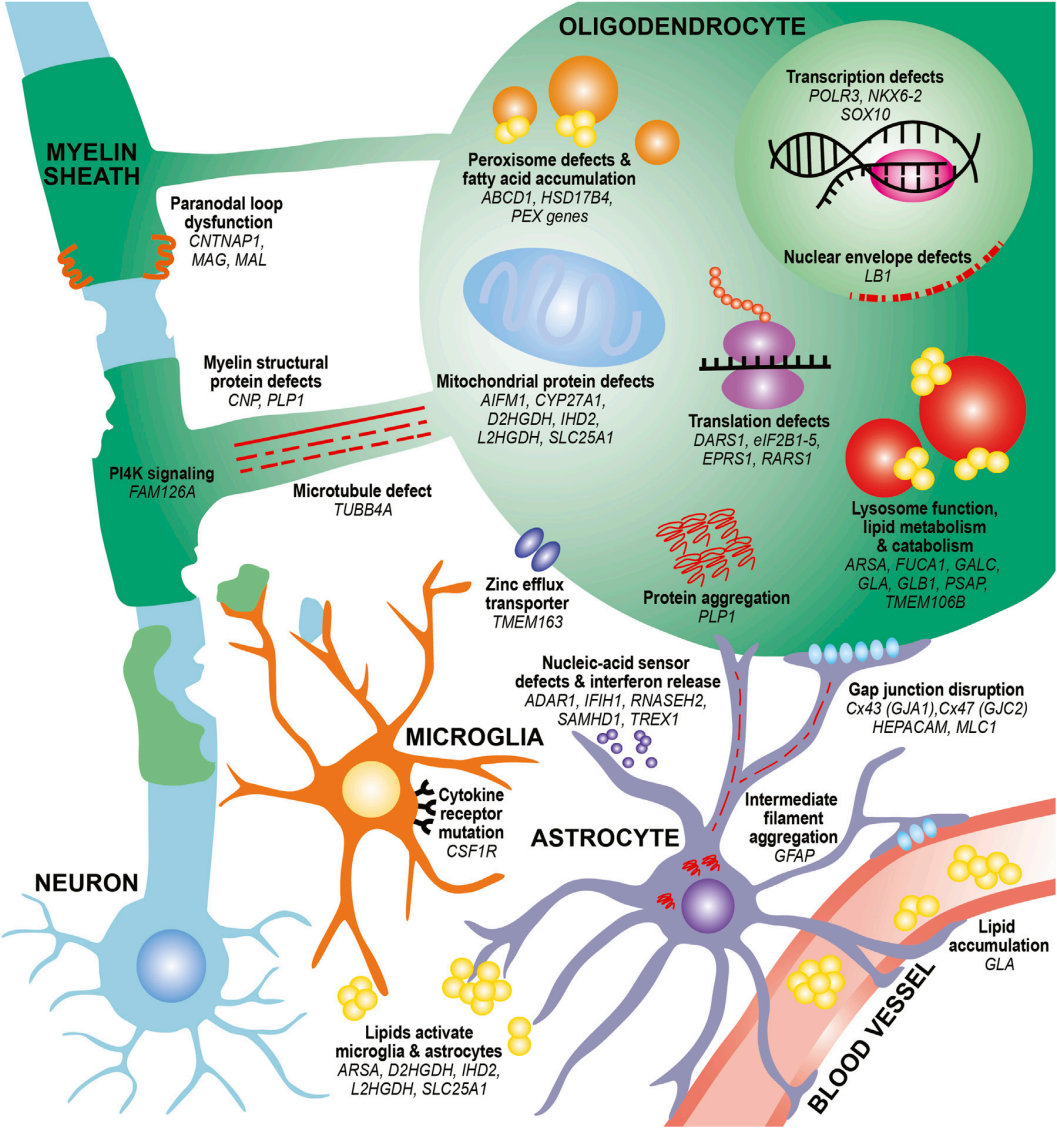
Diverse mechanisms and cell types contribute to hypomyelination in leukodystrophies. In oligodendrocytes, mutations in myelin structural proteins (*CNP*, *PLP1*) can lead to thinner myelin sheaths and paranodal loop malformation (*CNTNAP1*, *MAG*, *MAL*). PLP mutations can also lead to aberrant protein aggregation. Other mutations can affect the function of organelles that participate in lipid metabolism and catabolism, such as peroxisomes (*ABCD1*, *HSD17B4*, *PEX* family genes) and lysosomes (*ARSA*, *FUCA1*, *GALC*, *GLA*, *GLB1*, *PSAP*). These defects can also lead to toxic lipid accumulation that builds up in the bloodstream (*GLA*) or activate astrocytes and microglia (*ARSA*). Finally, mutations affecting transcription factors (*NKX6-2*, *SOX10*), transcriptional machinery (*POLR3* genes) and translation machinery (*DARS1*, *eIF2B* genes, *EPRS1*, *RARS1*) cause impair differentiation of oligodendrocytes and reduce production of myelin proteins. Defects in microtubules that project toward and within the myelin sheath (*TUBB4A*) as well as in PI4K signaling pathways that contribute to wrapping (*FAM126A*) can affect myelin sheath growth. Mutations in mitochondrial genes can lead to lipid processing defects (*CYP27A1*), aberrant activation of apoptotic pathways (*AIFM1*), and metabolic issues (*D2HGDH*, *IHD2*, *L2HGDH*, *SLC25A1*). Other pathways, such as nuclear envelope integrity (*LB1*) and zinc efflux (*TMEM163*) can also affect oligodendrocyte health. In astrocytes, intermediate filament protein mutations (*GFAP*) can lead to its aggregation and formation of Rosenthal fibers. Mutations affecting gap junctions can disrupt cell junctions between astrocytes and oligodendrocytes (*GJA1*, *GJC2*) and between astrocytes and vasculature (*HEPACAM*/*GLIALCAM*, *MLC1*). Mutations affecting nucleic-acid sensors (*ADAR1*, *IFIH1*, *RNASEH2* genes, *SAMHD1*, *TREX1*) can activate an immune response and release of interferons from astrocytes. In microglia, mutations in cytokine receptors (*CSF1R*) can affect microglial proliferation, differentiation, and activation. Finally, mutations affecting lipid processing (*ARSA*, *D2HGDH*, *IHD2*, *L2HGDH*, *SLC25A1*) can lead to aberrant lipid accumulation, which can activate microglia and astrocytes.

In this review, we provide an updated overview of 35 hypomyelinating leukodystrophies and home in on cellular mechanisms for disease. We find many common mechanisms affecting oligodendrocytes, the brain cells responsible for making myelin sheaths. These mechanisms often affect specialized oligodendrocyte functions, such as defects in myelin protein production, lipid processing, and peroxisome health. Additional oligodendrocyte-centric mechanisms include metabolic dysfunction, transcriptional and translational alterations, cytoskeletal dysregulation, and defects in cell junctions. Other glial cells, including astrocytes and microglia, may also be involved in certain leukodystrophies (Figure 1; Table 1). By identifying these emerging themes in cellular dysfunction, we hope to highlight conceptual insights that can contribute to the development of future therapeutic strategies for leukodystrophies.

**TABLE 1:** Leukodystrophy-causing mutations and their cellular mechanisms.

Disease type	Name	Gene(s)	Cell type	Cellular mechanism	Inheritance
**Lysosomal Lipid Metabolism and Catabolism Defects**	Fabry Disease (FD)	*GLA*	Unknown	Lysosomal enzyme α-galactosidase A (GLA) metabolizes globotriaosylceramide. Mutant α-galactosidase cannot break down glycolipids leading to accumulation in tissues.	X-linked Recessive, but female carriers can be affected
	Fucosidosis	*FUCA1*	Oligodendrocytes	Lysosomal α-l-fucosidase 1 (FUCA1) degrades fucose-containing glycoproteins and glycolipids. Mutant α-l-fucosidase cannot break down glycolipids and glycoproteins, leading to accumulation in tissues, reduction of oligodendrocyte differentiation, and increased apoptosis.	Autosomal Recessive
	GM1 Gangliosidosis	*GLB1*	Oligodendrocytes	Lysosomal enzyme β-galactosidase (GLB1) degrades glycosphingolipids into sphingosine, which is used in myelin synthesis. Mutant β-galactosidase cannot break down GM1 ganglioside, which accumulates in tissues and the brain.	Autosomal Recessive
	Krabbe Disease; Globoid-Cell Leukodystrophy (GCL)	*GALC*	Oligodendrocytes Macrophages	Lysosomal enzyme galactosylceramidase (GALC) catabolizes galactosylceramide and galactosylsphingosine (psychosine) during normal myelin turnover. Mutant GALC cannot catabolize sphingolipids, leading to accumulation and oligodendrocyte cell death. Lipid accumulation attracts macrophages, which form multinucleated globoid cells.	Autosomal Recessive
	Metachromatic Leukodystrophy (MLD)	*ARSA, PSAP*	Oligodendrocytes, Microglia, Macrophages	Lysosomal arylsulfatase A (ARSA) metabolizes sulfatides, which are abundant in myelin. Mutant ARSA cannot metabolize sulfatides, which accumulate and activate microglia and macrophages, leading to apoptosis and demyelination.	Autosomal Recessive
**Peroxisome Defects and Fatty Acid Accumulation**	D-Bifunctional Protein (DBP) Deficiency	*HSD17B4*	Neurons; Oligodendrocytes	DBP catalyzes β-oxidation of fatty acids. DBP deficiency in animal models leads to lipid accumulation, brain atrophy, and hypomyelination.	Autosomal Recessive
	X-linked Adreno-leukodystrophy (X-ALD)	*ABCD1*	Neurons, Astrocytes, Oligodendrocytes	*ABCD1* encodes adrenoleukodystrophy protein (ALDP), a peroxisomal transmembrane protein responsible for transporting very long-chain fatty acids (VLCFA) into peroxisomes. Mutations in *ABCD1* disrupt VLCFA metabolism. Accumulation of VLCFA is toxic to neurons, oligodendrocytes, and astrocytes.	X-linked recessive
	Zellweger Syndrome Spectrum Disorders (ZSDs)	*PEX1, PEX2, PEX3, PEX5, PEX6, PEX10, PEX11, PEX12, PEX13, PEX14, PEX16, PEX19, PEX26*	Oligodendrocytes	*PEX* genes assemble and allow transport into and out of peroxisomes. Mutations in *PEX* genes cause misassembly of peroxisomes, which dysregulates lipid synthesis and metabolism.	Autosomal Recessive
**Mitochondrial Protein Defects and Other Metabolic Deficiencies**	Cerebrotendinous Xanthomatosis (CTX)	*CYP27A1*	Microglia, Oligodendrocytes	Sterol-27 hydroxylase breaks down cholesterol into a bile acid. Sterol-27 hydroxylase deficiency leads to cholesterol-derivative accumulation.	Autosomal Recessive
	Hypomyelination with Spondylometa physeal Dysplasia (H-SMD)	*AIFM1*	Unknown	Mitochondrial apoptosis-inducing factor 1 (AIFM1) stabilizes complexes I and III through its redox activity. Mutant AIFM1 may not perform its electron-transport chain functions.	X-linked recessive
	2-Hydroxyglutaric Aciduria (2HGA)	*D2HGDH, IHD2, L2HGDH, SLC25A1*	Neurons, Astrocytes Oligodendrocytes, Microglia	Mitochondrial enzymes break down D-2-hydroxyglutarate and L-2-hydroxyglutarate. Various mitochondrial enzymes cannot metabolize 2-hydroxyglutarate, leading to oxidative stress, metabolic dysregulation, glial cell death, and myelin degradation.	Autosomal Recessive; Autosomal Dominant (Type II D-2-HGA)
	Canavan Disease	*ASPA*	Oligodendrocytes	Aspartoacylase (ASPA) catabolizes N-acetyl-l-aspartic acid (NAA) into acetate and aspartate. ASPA deficiency leads to accumulation of NAA and deficiency of acetate and aspartate in oligodendrocytes, thus reducing myelin production.	Autosomal Recessive
**Cytoskeletal Proteins**	Alexander Disease (AxD)	*GFAP*	Astrocytes	Glial fibrillary acidic protein (GFAP) is an astrocyte-specific intermediate filament protein. GFAP overexpression leads to aggregation (Rosenthal fibers) and cell toxicity.	Autosomal Dominant; *De novo*
	Autosomal Dominant Leukodystrophy with Autonomic Disease (ADLD)	*LMNB1*	Oligodendrocytes	Lamin B1 is an intermediate filament that builds the nuclear lamina. Gene duplication of *LMNB1* causes overexpression, affecting expression of lipid synthesis genes.	Autosomal Dominant
	Hypomyelination with Atrophy of the Basal Ganglia and Cerebellum (H-ABC)	*TUBB4A*	Oligodendrocytes, Neurons	*TUBB4A* encodes the microtubule protein β-tubulin. Mutations at the α-β dimer interface affect tubulin dynamics and motor protein binding, which can cause reduced myelination and cell death.	Autosomal Dominant
**Transcription Defects**	4H Leukodystrophy; POLR3-related Leukodystrophy	*POLR3A, POLR3B, POLR3C, POLR3K*	Oligodendrocytes	RNA polymerase III (POLR3) transcribes DNA into RNA. Mutations affect assembly of the RNA polymerase III complex and its DNA binding properties, thus reducing transcription of rRNA and tRNA.	Autosomal Recessive
	*NKX6-2*-related Spastic Ataxia with Hypomyelination (SPAX8)	*NKX6-2*	Oligodendrocytes	NKX6-2 is a transcription factor involved in oligodendrocyte differentiation and myelination.	*De novo*
	Waardenburg–Hirschsprung disease (WS4)	*SOX10*	Oligodendrocytes, Schwann cells	SOX10 is a transcription factor involved in oligodendrocyte differentiation and myelination.	*De novo*
**Translational Machinery**	tRNA Synthetase-related Leukodystrophies	*DARS1, EPRS1, RARS1*	Oligodendrocytes, Neurons	Mutations affecting proteins in the tRNA synthetase complex may disrupt protein translation	Autosomal Recessive
	Vanishing White Matter (VWM) Disease; Childhood Ataxia and Cerebral Hypomyelination (CACH)	*EIF2B1, EIF2B2, EIF2B3, EIF2B4, EIF2B5*	Oligodendrocytes	Eukaryotic translation initiation factor 2B (EIF2B) initiates mRNA translation and is a GTPase. Mutations impair translational control, thus reducing OPC differentiation and myelin production.	Autosomal Recessive
**Cell Junction Defects**	Megalencephalic Leukoencephalopathy with Subcortical Cysts (MLC); van der Knaap Disease	*MLC1, HEPACAM (GLIALCAM)*	Astrocytes, Oligodendrocytes	MLC1 and HEPACAM (GLIALCAM) contribute to astrocytic osmoregulation. Mutations can lead to gliovascular coupling defects, causing fluid buildup and macrocephaly.	Autosomal Recessive
	Oculodentodigital Dysplasia (ODDD)	*GJA1 (Cx43)*	Astrocytes, Oligodendrocytes	Gap junction α1/Connexin 43 create astrocyte gap junctions. Mutations disrupt gap junction formation and function between astrocytes and oligodendrocytes.	Autosomal Dominant; Autosomal Recessive; *De novo*
	Pelizaeus-Merzbacher-like Disease (PMLD)	*GJC2 (Cx47)*	Astrocytes; Oligodendrocytes	Mutations in connexin 47 disrupt gap junction function and electrical coupling.	Autosomal Recessive
**Myelin Structural Protein Defects**	*CNP*-related hypomyelinating leukodystrophy	*CNP*	Oligodendrocytes	Mutant 2′,3′-cyclic nucleotide-3′-phosphodiesterase (CNP) protein is expressed at very low levels.	Autosomal Recessive
	*CNTNAP1*-related Arthrogryposis and Leukodystrophy	*CNTNAP1*	Neurons, Oligodendrocytes	*CNTNAP1* encodes the paranodal axonal cell-adhesion protein contactin-associated protein 1 (Caspr1). Patient sural nerve EMs lack paranodal junctions.	Autosomal Recessive
	*MAG-*related PMLD	*MAG*	Oligodendrocytes	Mutant myelin-associated glycoprotein (MAG) is retained in the ER, resulting in misshapen paranodes and thin myelin sheaths.	Autosomal Dominant
	*MAL*-related Leukodystrophy	*MAL*	Oligodendrocytes	Myelin and lymphocyte (MAL) protein is important for the structure of paranodal loops. Mutant MAL accumulates in the ER, indicative of protein misfolding.	*De novo*
	Pelizaeus–Merzbacher Disease (PMD)	*PLP1*	Oligodendrocytes	PLP is the most abundant protein in myelin and important for compaction. Mutant PLP protein can misfold and aggregate. Disruption of iron metabolism may occur.	X-linked Recessive
**Other Transmem-brane Protein Defects**	Hypomyelination with Congenital Cataracts (HCC)	*FAM126A*	Oligodendrocytes	*FAM126A* encodes hyccin, a transmembrane protein expressed in the brain. Hyccin is a component of the PI4K complex that is important for phosphoinositide signaling, which regulates myelin wrapping.	Autosomal Recessive
	*SLC35B2*-related Chondrodysplasia with Hypomyelinating Leukodystrophy	*SLC35B2 (PAPST1)*	Unknown	SLC35B2 is a PAPS transporter that is important for making sulfated proteoglycans. Mutant SLC35B2 loses its localization to Golgi.	Autosomal Recessive
	Transient Infantile Hypomyelinating Leukodystrophy-19 (HLD19)	*TMEM63A*	Oligodendrocytes	TMEM63A is a mechanosensitive ion channel. Mutant TMEM63A does not produce stretch-activated currents in response to mechanical stimulation.	Autosomal Dominant
	*TMEM106B*-related Hypomyelinating Leukodystrophy	*TMEM106B*	Oligodendrocytes, Neurons	TMEM106B is involved in lysosomal trafficking and acidification. Mutant TMEM106B disrupts these lysosomal functions and may reduce PLP trafficking.	*De novo*
	*TMEM163*-related Hypomyelinating Leukodystrophy	*TMEM163*	Oligodendrocytes	TMEM163 is a zinc efflux transporter. In cultured cells, mutant TMEM163 is hyperactive in zinc efflux.	*De novo*
**Immune System Activation**	Aicardi-Goutieres Syndrome (AGS)	*TREX1, RNASEH2A/B/C, SAMHD1, ADAR1, IFIH1*	Astrocytes, Oligodendrocytes	These genes encode the intracellular nucleic acid sensor machinery that induces astrocyte release of interferons and subsequent cell death when foreign or defective genetic material is detected.	Autosomal Recessive
	Hereditary Diffuse Leukoencephalo pathy with Spheroids (HDLS)	*CSF1R*	Microglia; Oligodendrocytes; Neurons	Mutant CSF1 receptor is defective in activating following CSF1 ligand binding, leading to defects in microglia function and demyelination.	Autosomal Dominant

## Lysosomal lipid metabolism and catabolism defects

Oligodendrocytes are extraordinary producers of membranes and each oligodendrocyte is capable of forming multiple myelin sheaths. For example, oligodendrocytes from the cortex region of the brain can make as many as 50 myelin sheaths (Hughes et al., 2013). Each oligodendrocyte can make an estimated 2 mm^2^ of membrane surface area, which is 200 times higher than a typical epithelial cell (Poitelon, Kopec, and Belin 2020). Thus, oligodendrocytes are one of the most lipid-rich cell types in the body and, therefore, perhaps more vulnerable to mutations affecting lipid production.

Many mutations associated with leukodystrophies affect pathways involved in the production or breakdown of myelin lipids. The three major classes of lipids found in the myelin sheath include cholesterol, phospholipids (e.g., plasmalogen, phosphatidylcholine), glycolipids (e.g., galactosylceramide and its sulfated form, sulfatide), and sphingomyelin. Early studies found CNS myelin to contain molar percentages of 40–46% cholesterol, 26% phospholipid, 19–20% glycolipid, and 4–6% sphingomyelin (O’Brien 1965; Norton and Poduslo 1973). Leukodystrophy mutations affect lysosomal enzymes that participate in both lipid metabolism and catabolism. Defects in lipid metabolism can lead to aberrant lipid accumulation and dysfunctional myelin growth. Myelin is dynamic and, in addition to developmental myelin growth, myelin turnover replaces old membranes. This involves the modification of myelin and lipid breakdown as well as the formation of organelles called myelinoid bodies within the myelin sheath (Meschkat et al., 2022). Though it is still unclear whether lysosomal function is necessary for myelin clearance, myelin turnover may explain why mutations in lysosomal enzymes affecting lipid catabolism can lead to disease. Thus, understanding the nuanced relationship between lysosomal lipid metabolism and catabolism could be crucial to understanding the following leukodystrophies.

### Fabry disease (FD)

Fabry disease is a lysosomal storage disorder characterized by deposits of glycosphingolipids in blood vessels and smooth muscle tissue (Sweeley and Klionsky 1963; Aerts et al., 2008). Patients present with clusters of dark red spots on the skin (angiokeratomas), cloudy corneas, episodes of pain localized to hands and feet (acroparesthesias), gastrointestinal symptoms, and hearing loss. Symptoms eventually progress to affect the heart and kidneys, with patient death usually attributed to renal failure (Schiffmann et al., 2009). The most severe cases of FD begin between 3 and 10 years of age (Hopkin et al., 2008), but large-scale metabolic screening of newborns revealed that undiagnosed later-onset FD is about 11 times more common (Spada et al., 2006).

FD is caused by mutations in the *GLA* gene encoding α-galactosidase A. This enzyme is involved in the catabolism of multiple glycoproteins, glycolipids, and polysaccharides (Brady et al., 1967). The majority of the nearly 600 mutations associated with FD are thought to cause a complete loss of function (Gal 2010)*. GLA* is located on the X chromosome, and thus FD-causing mutations are inherited in an X-linked pattern. Interestingly, women heterozygous for FD mutations are not unaffected but in fact also experience significant disease burden, including renal and cardiovascular involvement (Wang et al., 2007; Waldek et al., 2009). Indeed, women carriers experience lifespan reduction of ∼10–15 years while men with FD experience lifespan reduction of ∼20 years (MacDermot et al., 2001; 2001).

Hypomyelination is one facet of progressive FD. Both affected men and women over the age of 35 display white-matter lesions (WML) in the central nervous system (CNS) (Fellgiebel et al., 2005), but the mechanism for this is unclear. Changes in cerebral blood flow and vascular changes due to glycolipid deposits have been suggested to contribute to glial cell death and demyelination. However it is unclear if cerebral blood flow increases or decreases in FD. One MRI study found that FD patients have increased cerebral blood flow and reduced glucose metabolism (Moore et al., 2003; Vagli et al., 2020). However, both a *Gla* knockout mouse model of FD and human imaging studies found reduced cerebral blood flow (Itoh et al., 2001; Hilz et al., 2004). Thus, the mechanism for hypomyelination in FD remains a mystery.

There are two standard treatment courses for FD: enzyme replacement therapy and oral chaperone therapy. First, enzyme replacement consists of infusions of recombinant human GLA enzyme (Eng et al., 2001), which reduces sphingolipid levels in the urine, plasma, and tissues (Rombach et al., 2013) and has been shown to stabilize WML progression. However, it is time-consuming and does not cure FD (Fellgiebel et al., 2005). Second, oral chaperone therapy introduces a small molecule ligand that aims to stabilize mutant GLA and restore its enzymatic activity. In cultured patient fibroblasts, this ligand increased baseline GLA levels and reduced accumulated sphingolipids (Yam, Zuber, and Roth 2005; Benjamin et al., 2009). Oral chaperone therapy presents some benefits over enzyme replacement, because patients display improvements in cardiac and digestive symptoms and do not develop antibodies to the ligand (Wilcox et al., 2012; Lenders et al., 2016). However, OCT is only effective in patients with certain mutations, thus limiting its use (Germain et al., 2016; Hughes et al., 2017).

Very recently, five men with FD received stem cell therapy through transfusions of hematopoietic stem cells (HSCs) transduced with human *GLA* cDNA. All patients who received this treatment reached healthy reference levels of circulating GLA enzyme and remained above baseline levels indefinitely (Khan et al., 2021). Thus, stem cell therapy is a promising therapeutic for FD patients, but additional clinical studies are required before this option can become widely available to patients.

### Fucosidosis

Fucosidosis is a progressive lysosomal storage disorder with autosomal recessive inheritance pattern. Patients with fucosidosis present with aberrant facial features (enlarged head, lips, and tongue, small or malformed teeth, and flattened nose), impaired physical growth and intellectual ability, recurrent respiratory infections, progressive skeletal dysplasia, and neurodegeneration (Stepien, Ciara, and Jezela-Stanek 2020). The mean age of onset is ∼1 year old (Willems et al., 1991). Fucosidosis presents with myelin loss in the cerebellum and cerebrum that is visible by MRI (Steenweg et al., 2010; Jain et al., 2012). Some patients display rapidly progressing neurodegeneration resulting in death before the age of 10 (Loeb et al., 1969) while others have a life expectancy into young adulthood (Kousseff et al., 1976).

Fucosidosis is linked to mutations in *FUCA1* (α-l-fucosidase 1), that lead to enzymatic deficiency (Willems et al., 1988; 1991). This results in the inability to metabolize certain sugar compounds (e.g., fucose-containing glycolipids and fucose-containing glycoproteins), which then accumulate (Van Hoof and Hers 1968). A canine model of fucosidosis displayed reduced expression of key oligodendrocyte differentiation genes, including *CNP, PLP* (proteolipid protein)*, MAG, MAL, MRF, OPALIN*) as well as oligodendrocyte death that was confirmed with apoptosis markers. Because these defects were observed during developmental stages, they support a mechanism of hypomyelination rather than demyelination (Fletcher et al., 2011; 2014). In addition, a *Fuca1* knockout mouse exhibited lysosomal dysregulation, lipid accumulation, neuroinflammation, and behavioral deficits (e.g., impaired coordination and spatial learning) that parallel the motor and intellectual deficits observed in fucosidosis patients (Stroobants et al., 2018). Thus, both canine and murine disease models indicate that many downstream effects of FUCA1 deficiency contribute to myelin defects in fucosidosis.

Most potential fucosidosis treatment strategies aim to ameliorate symptoms. Canine studies have shown success with enzyme replacement, which reduces vacuolation, neuron death, and neuroinflammation. However, these therapies only reduce symptoms and do not stop neurological progression (Kondagari et al., 2011; 2015). Bone marrow transplantation has shown success in both canine models and one human patient, but the risks and side effects of this procedure may outweigh the benefits (Taylor, Farrow, and Stewart 1992; Vellodi et al., 1995; Miano et al., 2001). Thus, additional preclinical studies are needed in order for any of these approaches to move forward as viable therapies for fucosidosis.

### GM1 gangliosidosis

GM1 gangliosidosis is an autosomal recessive lysosomal storage disorder ranging from infantile-onset to chronic presentation. Patients experience intrauterine growth restriction, abnormal fluid accumulation in serous cavities, and placental vacuolization (Regier et al., 1993) coupled with the rapid progression of CNS dysfunction including blindness, deafness, seizures, and feeding difficulties. Pathologically, multiple brain regions including caudate, putamen, corpus callosum, basal ganglia, and cerebellar white matter are atrophied (Nestrasil et al., 2018). MRI scans and postmortem brain analyses revealed hypomyelination and atrophy of the caudate, putamen, corpus callosum, and basal ganglia (King et al., 2020; Uchino et al., 2020).

GM1 gangliosidosis is a result of homozygous or compound heterozygous (biallelic) mutations in the *GLB1* gene (Morrone et al., 2000; Bidchol et al., 2015). *GLB1* encodes for the ubiquitous lysosomal enzyme β-galactosidase (β-GAL), which is responsible for the degradation of GM1 ganglioside, a type of glycosphingolipid composed of a ceramide lipid tail linked to a glycan headgroup containing a sialic acid residue (Regier et al., 1993; Sipione et al., 2020). The breakdown of GM1 gangliosides creates the downstream catabolite sphingosine, which is used in sphingomyelin synthesis and ultimately myelin formation. Mutations in *GLB1* cause reduced or null β-GAL enzymatic activity, which leads to toxic and progressive accumulation of GM1 gangliosides and other glycoproteins (Kajihara et al., 2020).

Several studies have revealed cellular pathways involved in GM1 gangliosidosis pathogenesis, such as an activated unfolded protein response (Tessitore et al., 2004), calcium signaling in the endoplasmic reticulum (ER) (Sano et al., 2009), autophagy (Takamura et al., 2008), and inflammasome activation (Son et al., 2015). Additional studies could elucidate molecular and cellular mechanisms of hypomyelination in GM1 gangliosidosis.

Recent studies have focused on two potential therapeutic approaches. First, multiple studies have used viruses, such as AAV9 (adeno-associated virus), a virus with broad CNS tropism, to deliver a functional copy of the *GLB1* gene. In one study, *Glb1* knockout mice received intravenous injections of AAV9 containing the *GLB1* gene, which increased β-GAL activity in various regions of the brain and in peripheral tissues. Interesting sex-specific effects were observed, including more viral copies in females and a significant difference in median survival from ∼100 to ∼577 days (in females) and ∼398 days (in males) (Weismann et al., 2015). In a more recent study, researchers used human iPSCs to create *GLB1* knockout cerebral organoids that recapitulated GM1 gangliosidosis by exhibiting progressive accumulation of gangliosides at 10 and 20 weeks in culture. Delivering AAV9 containing the *GLB1* gene significantly increased β-GAL activity and reduced GM1 accumulation in these organoids (Latour et al., 2019). Thus, enzyme replacement via stem cell therapy is a viable strategy for the treatment of GM1 gangliosidosis.

Researchers have also taken a drug screening approach. iPSCs from GM1 gangliosidosis patients were differentiated into neural stem cells, which model disease biochemistry by exhibiting accumulated GM1 gangliosides. High-content screening identified 25 small molecule compounds that reduced the accumulation of GM1 gangliosides. Of these, amodiaquine (a heme polymerase inhibitor) and thiethylperazine (an adrenergic antagonist) were the most effective. When administered *in vivo* to β-GAL null mice, these two compounds decreased GM1 ganglioside accumulation in the brain (Kajihara et al., 2020). Thus, researchers are exploring both stem cell-based enzymatic replacement and drug therapies as therapeutic strategies to treat GM1 gangliosidosis.

### Krabbe disease

Krabbe disease, also known as globoid-cell leukodystrophy (GCL), is a rapidly progressive autosomal recessive neurodegenerative disorder. Patients have rapid demyelination resulting in near total loss of myelin, severe astrogliosis, and the presence of multinucleated globoid cells in the white matter (Suzuki and Suzuki 1970). Hypomyelination in the CNS leads to muscle spasticity, paralysis, irritability, loss of vision and hearing, seizures, peripheral neuropathy, and premature death (Hagberg, Sourander, and Svennerholm 1963; Morse and Rosman 2006; Sakai 2009; Brodsky and Hunter 2011; Beltran-Quintero et al., 2019). The median survival is 1.5 and 9.5 years for early- and late-infantile Krabbe disease, respectively, whereas juvenile- and adult-onset subtypes have projected survival beyond 30 years of age (Komatsuzaki et al., 2019).

Krabbe disease patients have mutations in the *GALC* (galactosylceramidase) gene that result in loss of function of this lysosomal enzyme (Wenger and Luzi 2020). GALC removes the sugar galactose from galactosylceramide, galactosylsphingosine (psychosine), and other galactolipids (Suzuki and Suzuki 1970; Beltran-Quintero et al., 2019; Komatsuzaki et al., 2019). The inability to catabolize galactosylceramide and galactosylsphingosine leads to their accumulation. On the one hand, galactosylceramide accumulation is postulated to attract macrophages, which phagocytose it, then form multinucleated globoid cells (Komatsuzaki et al., 2019). These globoid cells are associated with severe demyelination, axonopathy, and neuronal death (Wenger and Luzi 2020). On the other hand, psychosine accumulation is cytotoxic to oligodendrocytes (Komatsuzaki et al., 2019) and therefore may be the pathological driver of Krabbe disease symptoms. Indeed, in a human oligodendroglioma cell line, treatment with psychosine caused the mislocalization of fatty acid binding protein 5 (FABP5) to the mitochondria, the formation of mitochondrial pores, and reduced levels of mitochondrial DNA. The authors postulated that this could lead to oxidative stress and apoptosis (Cheng, Kawahata, and Fukunaga 2020). Thus, loss of GALC activity results in aberrant lipid accumulation and various downstream effects.

Surprisingly, of the >70 disease-causing mutations in *GALC*, most are not located in the enzymatic domain. For example, in the D528N mutation, the aberrant asparagine residue leads to hyperglycosylation and misfolding of GALC (Lee et al., 2010). This glycosylation defect is reminiscent of the D32N MPZ (myelin protein zero) mutation in Charcot-Marie-Tooth neuropathy. In the peripheral nervous system (PNS), this mutation leads to hyperglycosylation of MPZ and defects in MPZ trafficking to the plasma membrane (Prada et al., 2012). This is consistent with experiments in cell lines in which mutating the MPZ glycosylation site can lead to a membrane adhesion defect (Filbin and Tennekoon 1993), which is hypothesized to impact myelin compaction. Thus, Krabbe disease mutations can affect both enzymatic activity as well as post-translational modifications (PTMs).

Currently, multiple potential therapeutic strategies for Krabbe disease exist. HSC transplant has been performed and is most successful when it precedes symptom onset. Indeed, to facilitate early diagnosis and treatment, several states (e.g., New York, Ohio, Pennsylvania, Kentucky, Indiana, etc.) require all newborns to be screened within 48 h of birth (Orsini et al., 2016). Unfortunately, long-term outcomes of HSC transplant (regardless of whether the treatment was initiated prior to symptom onset) are poor and high-risk with elevated morbidity rates (Krivit et al., 1998; Escolar et al., 2005; Aldenhoven and Kurtzberg 2015; Wasserstein et al., 2016; Wright et al., 2017).

Ongoing research in mice has focused on another approach: viral delivery of the *Galc* gene. The *twitcher* mouse, which has a *Galc* mutation, is often used to model Krabbe disease. By postnatal day 30, these mice exhibit severe myelin loss, astrocytic gliosis, and the presence of globoid cells (Kobayashi et al., 1980; Li and Sands 2014). Several studies have looked at viral vector delivery of *Galc* to presymptomatic *twitcher* mice. Two studies administered AAV9 encoding mouse *Galc* via intrathecal (Karumuthil-Melethil et al., 2016) and intracisternal injections (Bradbury et al., 2020). Both studies found reduced galactosylsphingosine levels, ameliorated myelin abnormalities, and ultimately prolonged survival of the *twitcher* mice. Another study used a virus with a different serotype (AAVrh10) and a different delivery method (intravenous administration on postnatal day 10). This virus crossed the blood–brain barrier, and prevented both myelin loss and galactosylsphingosine accumulation (Rafi, Luzi, and Wenger 2021). Thus, these promising results in rodents may open the door for future pre-clinical studies on viral delivery of the *Galc* gene.

### Metachromatic leukodystrophy (MLD)

MLD is an autosomal recessive demyelinating disorder that can be divided into four subtypes according to age of onset: late infantile, early-juvenile, late-juvenile, and adult-onset (Von Figura, Gieselmann, and Jaeken 2001; Sevin, Aubourg, and Cartier 2007). The most common form of MLD, which comprises ∼50% of all cases, is the late infantile subtype, which has symptom onset by ∼2 years of age (Cesani et al., 2016). Clinical presentations include muscle wasting, weakness and rigidity, developmental delays, progressive vision loss, seizures, paralysis, and dementia (Gieselmann and Krägeloh-Mann 2010). In the majority of cases, the prognosis is severe and often leads to a persistent vegetative state or death within a few years of symptom onset (Von Figura, Gieselmann, and Jaeken 2001; Gieselmann and Krägeloh-Mann 2010; Aubourg et al., 2011).

Most MLD subtypes can be traced back to >150 mutations in the gene encoding the enzyme arylsulfatase A enzyme (ARSA or ASA) (Von Figura, Gieselmann, and Jaeken 2001; Aubourg et al., 2011; Batzios and Zafeiriou 2012). In rare instances, MLD can also be caused by a mutation in the *PSAP* gene encoding presaposin, the precursor to saposin B (SAP-B), an activator of ARSA (Holtschmidt et al., 1991). Because ARSA is a lysosomal enzyme, MLD is often referred to as a lysosomal storage disorder. Mutations in *ARSA* lead to an enzymatic deficiency, ultimately resulting in the impaired metabolism of sulfatide, a major lipid component of the myelin sheath. Sulfatide can accumulate in microglia, neurons, and oligodendrocytes along with peripheral tissues (Gieselmann 2003; Molander-Melin et al., 2004; Patil and Maegawa 2013). Excess sulfatide triggers an inflammatory response which includes microglial activation, astrogliosis, and recruitment of peripheral macrophages. Together, these events are thought to cause apoptosis of glia and neurons as well as demyelination in the CNS (Sevin, Aubourg, and Cartier 2007; Patil and Maegawa 2013). Loss of function in MLD is also supported by a global *Arsa* knockout mouse that displays many clinical hallmarks of MLD, such as sulfatide accumulation, motor dysfunction, and cognitive impairment. However, these mice lacked shortened lifespans and white matter defects (Hess et al., 1996). Thus, additional research should clarify the mechanistic link between sulfatide accumulation and demyelination.

One therapeutic approach for MLD is enzyme replacement. Recent Phase 1 and 2 clinical trials evaluated the safety and efficacy of intravenous delivery of recombinant human ARSA. 13 children with MLD received ARSA at 50, 100 or 200 U/kg every 2 weeks for 1 year. The results revealed that cerebrospinal fluid (CSF) sulfatide levels significantly decreased in the 100 and 200 U/kg groups, but perplexingly increased in the 50 U/kg group. Nevertheless, despite improvements in sulfatide levels, the other efficacy variables, including MRI, and motor and cognitive function, showed either no change or worsening measurements (Í Dali et al., 2021). It is unclear whether intravenously administered ARSA can cross the blood–brain barrier and this could explain the lack of efficacy in the CNS. This is further confounded by a study indicating that different cell types have varying levels of ARSA uptake. In primary cultures of neurons, microglia, astrocytes, and oligodendrocytes treated with ARSA for 24 h, microglia had the highest uptake, whereas oligodendrocytes had less uptake (Kaminski et al., 2021). Thus, peripheral administration of recombinant ARSA has yet to yield promising results.

Another therapeutic approach for MLD is HSC transplant with the goal for donor cells to secrete functional ARSA (Rosenberg et al., 2016). Early work demonstrated that transplanted bone marrow cells expressing green fluorescent protein (GFP) from transgenic mice could be detected in the brains of wildtype recipients (Brazelton et al., 2000; Mezey et al., 2000) and these engrafted cells expressed microglia markers (Priller et al., 2001). In a clinical trial with three MLD patients, patient-derived HSCs were transduced with a lentivirus expressing a functional copy of ARSA, then reinfusion into patients. This led to functional ARSA expression and halting of disease progression (Biffi et al., 2013). Additional HSC transplant studies also report delays in disease progression (van Egmond et al., 2013; Groeschel et al., 2016; van Rappard et al., 2016). Thus, HSC transplantation is a promising approach for MLD treatment.

## Peroxisome defects and fatty acid accumulation

Peroxisomes are organelles that play multiple important roles in lipid metabolism and the building of the myelin sheath. First, peroxisomes perform β-oxidation of fatty acids, such as very-long-chain fatty acids (VLCFAs), pristanic acid, long-chain dicarboxylic acids, and some eicosanoids and polyunsaturated fatty acids (PUFAs). Though mitochondria also perform β-oxidation of fatty acids, each organelle displays substrate exclusivity. Second, peroxisomes synthesize plasmalogens, a type of ether-phospholipid found in myelin (Kassmann 2014). Thus, it is not surprising that multiple leukodystrophies can be attributed to mutations in genes encoding peroxisomal proteins.

### D-Bifunctional Protein (DBP) deficiency

DBP deficiency is an autosomal recessive disorder caused by mutations to the *HSD17B4* (hydroxysteroid 17-β dehydrogenase 4) gene that encodes DBP. DBP enzyme catalyzes steps in the peroxisomal β-oxidation of fatty acids. Subtypes of this leukodystrophy are classified according to which DBP domains are affected. Type I, the most severe, is caused by mutations in both the hydratase and dehydrogenase domains; Type II is caused by mutations in the hydratase domain; Type III caused by mutations in the dehydrogenase domain (Ferdinandusse et al., 2006b). The patient phenotype includes abnormal facial features, reduced muscle tone, seizures within the first month of life, intellectual and sensory disability, increased plasma VLCFAs, as well as cerebellar atrophy, progressive white matter dystrophy, and demyelination visualized on MRI (van Grunsven et al., 1999; Ferdinandusse et al., 2006a; Yamamoto et al., 2021).

Several DBP deficient animal models have been generated. Zebrafish embryos lacking DBP display lipid accumulation, general growth delays, aberrant axon development, and reduced myelination (Kim et al., 2014). Interestingly, a neuronal-specific knockout mouse developed motor symptoms and cerebellar atrophy, but an oligodendrocyte-specific knockout mouse had no clinical features (Verheijden et al., 2013). These results hint at intricate neuron-glia interactions and highlight the need to untangle the precise mechanisms by which DBP deficiency causes a range of symptoms.

### X-linked adrenoleukodystrophy (X-ALD)

X-ALD encompasses four distinct presentations: a severe childhood type (ALD), the milder adrenomyeloneuropathy (AMN), adrenal insufficiency (Addison’s Disease) and an asymptomatic form. The childhood form (ALD) typically begins between ages 4–10 with neurological symptoms that include worsening vision, difficulty swallowing, poor coordination, and seizures. Patient death from ALD typically occurs within a few years. Adult-onset AMN is the most common X-ALD and presents with weakness in the legs (paraparesis), changes in cognitive ability, and urogenital tract disorders. Adrenal insufficiency rarely presents with neurological symptoms (Berger, Forss-Petter, and Eichler 2014; Turk et al., 2020).

X-ALD is caused by mutations on the X chromosome in the *ABCD1* gene that encodes adrenoleukodystrophy protein (ALDP). ALDP is a peroxisomal transmembrane protein involved in the transport of VLCFAs into peroxisomes for their subsequent β-oxidation (Wiesinger et al., 2013). ALDP deficiency causes increased plasma VLCFA levels and VLCFA accumulation in the adrenal cortex, testes, and brain regions preceding demyelination (Theda et al., 1992). However, it is unclear whether increasing levels of VLCFA contribute to disease severity. On the one hand, VLCFAs are toxic to cultured rat neurons, oligodendrocytes, and astrocytes, perhaps by altering inner mitochondrial membrane permeability (Hein et al., 2008). On the other hand, plasma VLCFA levels in ALD and AMN are similar, even though the AMN phenotype is far less severe (Moser et al., 1999; Stradomska and Tylki-Szymańska 2009; Stradomska et al., 2020). Moreover, mouse models expressing truncated deficient ALDP exhibit increased VLCFA levels and impaired β-oxidation, but no demyelination in adulthood (Forss-Petter et al., 1997; Kobayashi et al., 1997; Lu et al., 1997). By contrast, *Abcd1*-null mice display late-onset locomotion defects, and axonal degeneration in the spinal cord and sciatic nerve beginning ∼15 months of age (Pujol et al., 2002); therefore, this mouse more appropriately models AMN than ALD.

A number of approaches to treating ALD have been investigated. First, Lorenzo’s oil, a mixture of two long-chain fatty acids, can reduce plasma levels of VLCFA by competitively inhibiting their synthesis (Sassa et al., 2014). Second, bone marrow transplantation during the early stage of the disease led to reduced plasma VLCFA levels and stabilization or improvement of demyelinating lesions on MRI scans (Shapiro et al., 2000). Finally, patients transfused with HSCs that were transduced *ex vivo* with *ABCD1* cDNA showed a halt in disease progression and even began to express functional ALDP (Eichler et al., 2017). Thus, basic understanding of peroxisome function and disease mechanism has contributed to promising steps towards the treatment of X-ALDs.

### Zellweger syndrome spectrum disorders (ZSDs)

ZSDs are a set of neonatal-onset progressive leukodystrophies that include neonatal adrenoleukodystrophy (NALD) and Infantile Refsum disease. In newborns, symptoms include poor muscle tone, trouble with feeding, seizures, renal cysts, liver dysfunction, skeletal abnormalities, and hearing and vision loss. Children with ZSD have varying levels of symptom severity. Infants with severe ZSD rarely survive past the first year of life. Intermediate cases may show progressive hearing and vision loss as well as fatal myelin degeneration during childhood (Steinberg et al., 2003). Non-progressive cases often result in children surviving until school age with the possibility of normal intellect (Poll-The et al., 2004).

ZSDs are classified as peroxisome biogenesis disorders, because they are caused by autosomal recessive mutations in any of at least 12 peroxin (*PEX*) genes that are responsible for peroxisome assembly. The most commonly mutated genes are *PEX1* and *PEX6*. The most severe cases usually result from loss-of-function mutations, such as large deletions and nonsense mutations (Steinberg et al., 2003). The prevailing hypothesis for the cause of ZSD posits that deficiencies in peroxisome assembly can cause dysregulation of fatty acid metabolism and subsequent accumulation of long-chain fatty acids, which may lead to membrane disruption and cellular toxicity. Indeed, when compared to unaffected brains, ZSD brains have higher levels of certain species of lipids (ceramide monohexosides, cholesterol ester, dipalmitoyl phosphatidylcholine and dipalmitoyl phosphatidylserine) (Steinberg et al., 2006; Saitoh et al., 2007). Interestingly, mouse studies indicate that cholesterol homeostasis and peroxisome assembly may be intricately connected. *Lrp1* knockout mice with dysregulation of cholesterol homeostasis display aberrant peroxisome assembly and impaired oligodendrocyte maturation (Lin et al., 2017).

In mouse models, cell-specific knockout of *Pex5* indicates that many cell types contribute to ZSD pathology. Knockout of *Pex5* in both neurons and glia results in demyelination, axonal degeneration, lack of muscle control (ataxia), tremors, and premature death ∼6 months of age (Hulshagen et al., 2008). Knockout of *Pex5* only in oligodendrocytes recapitulates these phenotypes, but mice die later, at ∼12 months of age (Kassmann et al., 2007). However, knockout of *Pex5* in either neurons or astrocytes results in high VLCFA and reduced plasmalogens levels, but no behavioral phenotypes and unperturbed axonal integrity (Bottelbergs et al., 2010). Together, these studies show that while peroxisome dysfunction in oligodendrocytes can lead to ZSD phenotypes, contributions by peroxisomes in neurons and astrocytes likely play a role in ZSD pathology as well.

Currently, no cure exists for ZSD and treatments focus on managing symptoms (e.g., gastrostomy to provide nutrition, hearing aids for hearing loss). Unfortunately, dietary supplementation of lipids has not been able to affect disease course. An early study found that ZSD patients had very low levels of docosahexaenoic acid (DHA) in the brain, retina and other tissues (Martinez 2001). However, in a clinical trial, DHA supplementation for 1 year did not improve the visual function of individuals with peroxisome assembly disorders (Paker et al., 2010). Thus, additional therapeutic approaches should be considered for ZSD.

## Mitochondrial protein defects and other metabolic deficiencies

Though mitochondria are commonly known as the “powerhouse of the cell” due to their role in the efficient production of adenosine triphosphate (ATP), they also have additional functions, such as regulation of cell death and metabolism of cholesterol. Several leukodystrophies can be attributed to mutations in genes encoding mitochondrial-specific proteins with diverse functions as well as other genes involved in cellular metabolism.

### Cerebrotendinous xanthomatosis (CTX)

CTX is an autosomal recessive disorder caused by mutations in the gene encoding the mitochondrial enzyme sterol 27-hydroxylase (CYP27A1). These mutations result in defective versions of this enzyme with low or undetectable enzyme activity (Cali et al., 1991; Carson and De Jesus 2021). CTX is a lipid storage disease characterized by disruptions in bile acid synthesis. Normally, cholesterol is metabolized into a bile acid called chenodeoxycholic acid (CDCA) by CYP27A1. As a result of reduced feedback (i.e., lack of CDCA), cholesterol synthesis is upregulated, resulting in aberrant accumulation of the byproduct cholestanol in various tissues and organs (Serizawa et al., 1982; Carson and De Jesus 2021).

Sites of localized cholestanol accumulation, such as the brain, eyes, arteries, and tendons, reflect the clinical manifestation of CTX. Around puberty, CTX patients begin to show neurological signs and symptoms, such as cerebellar ataxia, pseudobulbar affect, and brain atrophy (Carson and De Jesus 2021). Furthermore, MRI scans revealed white matter atrophy in the dentate nuclei (Vanrietvelde et al., 2000; Chang et al., 2010). Other clinical features of CTX include cataracts, atherosclerosis, and fatty tumors on tendons and muscles. As the disease progresses, patients can develop paralysis and dementia. Eventually cholestanol deposits affect the brainstem and patient death occurs during mid-to-late adulthood from cardiac arrest (Carson and De Jesus 2021).

Many studies support the mechanistic link between CYP27A1 deficiency and cholestanol accumulation in the brain. An early study of postmortem CTX brains revealed that cholestanol preferentially accumulates in myelin fractions while healthy brain fractions had no detectable cholestanol (Stahl, Sumi, and Swanson 1971). Another study found that feeding mice 1% cholestanol led to significant accumulation of cholestanol in the cerebellum, suggesting that peripheral cholestanol can cross the blood–brain barrier (Byun et al., 1988). *Cyp27a1* knockout mice, which have increased cholesterol synthesis and a two-fold increase in cholestanol levels in peripheral tissues, display a striking 12-fold increase in cholestanol levels in the cerebellum, indicating that cholestanol preferentially accumulates in the brain (Båvner et al., 2010). Indeed, a recent RNA-seq database from mouse brains indicates that *Cyp27a1* mRNA is more highly enriched in oligodendrocytes than other brain cell types (e.g., neurons, astrocytes, microglia/macrophages) (Zhang et al., 2014). Thus, these studies suggest that oligodendrocyte-specific CYP27A1 activity and tissue-specific cholestanol accumulation in the brain play important roles in CTX.

CTX is currently treated through dietary supplementation therapy. CDCA supplementation lowers the accumulation of cholestanol in both plasma and CSF, likely by exerting negative feedback on cholesterol synthesis, and has led to improvements in many clinical symptoms, including cataracts, dementia, neuropathy, and other neurological symptoms (Salen, Meriwether, and Nicolau 1975; Berginer, Salen, and Shefer 1984; Duell et al., 2018). This treatment has shown success when implemented before the age of 25 (Yahalom et al., 2013). However, CDCA is hepatotoxic, prompting some studies to suggest cholic acid as an alternative treatment for patients who tolerate CDCA poorly (Pierre et al., 2008; Koyama et al., 2021). In addition, a recent study in a mouse model of CTX used an AAV to express *CYP27A1* in the liver and this reestablished bile acid metabolism and restored normal plasma bile acid levels (Lumbreras et al., 2021). Thus, promising steps have been made in the management and treatment of this leukodystrophy, though more translational and clinical studies are needed.

### Hypomyelination with spondylometaphyseal dysplasia (H-SMD)

H-SMD is a disorder characterized by abnormal growth and development of the vertebrae and bone growth plates (metaphyses), as well as significant lack of myelin on MRI. Beginning between 1 and 2 years of age, patients may start to lose the ability to walk, which may slowly progress into motor deterioration, spasticity and tremors. Other symptoms include mild cognitive impairment, bone and joint issues such as scoliosis, and visual symptoms such as involuntary eye movement (nystagmus) (Kettwig et al., 2015; Miyake et al., 2017).

Multiple studies have found mutations in the *AIFM1* (apoptosis-inducing factor, mitochondria-associated 1) gene in H-SMD. AIFM1 has dual functions: 1) to regulate the assembly of the mitochondrial machinery that participate in oxidative phosphorylation (Vahsen et al., 2004), and 2) to act as a signal for cell death when it translocates to the nucleus (Susin et al., 1999). An early study discovered mutations in affected sons born to asymptomatic mothers, indicative of X-linked recessive inheritance (Bieganski, Dawydzik, and Kozlowski 1999). Most H-SMD mutations are missense mutations localized to a 70-base-pair region in exon seven of *AIFM1*. However, some cases involve intronic mutations and synonymous variant mutation (a codon substitution that doesn’t change the encoded amino acid) (Mierzewska et al., 2017; Edgerley et al., 2021). Conflicting evidence exists on whether *AIFM1* mutations in H-SMD cause loss of function. In one study, patient osteoblast cells displayed significantly reduced *Aifm1* mRNA and protein levels, which the authors suggest could be due to bioinformatically predicted mRNA splicing disruptions (Miyake et al., 2017). However, comparisons of four mutant AIFM1 proteins showed that the extent of structural alterations and changes in redox activity vary by mutation (Sevrioukova 2016), which indicates that only some AIFM1 mutations lead to loss of function. Hence, the molecular mechanisms underlying hypomyelination in H-SMD are still unclear.

### 2-Hydroxyglutaric aciduria (2HGA)

2HGA is an autosomal recessive neurometabolic disorder characterized by the accumulation of 2-hydroxyglutarate (2-HG). 2-HG is a side product of nonspecific Krebs Cycle enzyme activity and is closely related to the Krebs Cycle intermediate ɑ-ketoglutarate (Rzem et al., 2007). There are three forms of 2HGA; each form is associated with mutations in specific mitochondrial genes and named according to which 2-HG enantiomer (L or D) is affected.

First, L-2-HGA (LHGA) primarily affects the cerebellum, presenting with ataxia, seizures, and an enlarged head (macrocephaly). LHGA is associated with mutations in the *L2HGDH* gene, which codes for a mitochondrial enzyme that converts L-2-HG into α-ketoglutarate. A deficiency in this enzyme causes increased levels of L-2-HG in urine, plasma and CSF (Barth et al., 1993; Rzem et al., 2004)*.*

Second, D-2-HGA (DHGA) falls into two categories. Type I is less severe with later onset and associated with mutations in the *D2HGDH* gene. Type II is more severe with earlier onset and can present with heart enlargement (cardiomyopathy). Unlike other 2HGA’s, Type II D-2-HGA is uniquely inherited in an autosomal dominant pattern and associated with mutations in the *IHD2* gene (Struys et al., 2005; Kranendijk et al., 2010)*.*

Finally, D,L-2-HGA is caused by mutations in the *SLC25A1* gene, which encodes a protein that transports citrate across the inner mitochondrial membrane. In cultured patient cells, lack of SLC25A1 activity results in the increased presence of both D-2-HG and L-2-HG (Nota et al., 2013)*.*

The mechanism for 2-HG accumulation leading to symptoms is likely due to two types of toxicity–oxidative damage and excitotoxicity. In primary neuron cultures, both L-2-HG and D-2-HG induce a number of metabolic effects, such as inhibiting mitochondrial creatine kinase and ATP synthase (Kölker et al., 2002; da Silva et al., 2003). In rats, intracerebroventricular administration of D-2-HG resulted in increased oxidative stress and upregulation of markers for reactive astrocytes and microglia (Ribeiro et al., 2021). Indeed, in *L2HGDH* knockout mice, vacuolar lesions appeared in oligodendrocyte and astrocyte cytoplasm as well as in myelin tracts in the cortex and corpus callosum (Rzem et al., 2015). In addition, 2-HG shares chemical structure similarity with the excitatory transmitter glutamate. D-2-HG treatment to cultured neurons led to excitotoxic cell damage (Kölker et al., 2002). In rats, a NMDA glutamate receptor antagonist administration prevented the formation of some oxidative species (Ribeiro et al., 2021). Thus, signs of both excitotoxicity and oxidative damage are observed in cell and animal models of 2HGA and these two types of damage might synergistically exacerbate symptoms in 2HGA.

Treatment of 2HGA primarily focuses on managing symptoms, especially seizures when they are present. One case study successfully managed patient tremors and reduced urinary 2-HG by supplementing with FAD and carnitine (Samuraki et al., 2008). Another found similar effects with riboflavin (Vitamin B2) supplementation (Yilmaz 2009). However, the mechanism of action for these supplements is unclear. Thus, further research is necessary to uncover effective treatments for 2HGA.

### Canavan disease

Canavan disease is a progressive, autosomal recessive leukodystrophy. It typically affects infants ∼3 months of age, who begin to develop symptoms that include lethargy, poor vision, little-to-no motor development, seizures, and other progressive neurological defects. Symptoms continue to worsen until the child dies ∼10 years of age (Bokhari et al., 2022).

Canavan disease results from a mutation in the gene encoding aspartoacylase (ASPA) (Bokhari et al., 2022). ASPA is an oligodendrocyte-enriched enzyme that hydrolyzes *N*-acetyl L-aspartic acid (NAA), which is primarily synthesized in neurons, into acetate and aspartate. Canavan disease patients are unable to catabolize NAA, which progressively accumulates with age in oligodendrocytes. Studies in rodent oligodendrocytes indicate that the products of NAA hydrolysis are important for oligodendrocyte survival and differentiation (Kumar et al., 2009). Indeed, acetate, one of the products of ASPA, is converted to acetyl-CoA, which is an important building block in lipid synthesis. The other product of ASPA, aspartate, can enter cell metabolic pathways (I Amaral et al., 2017), promote oligodendrocyte precursor cell (OPC) differentiation and transcription of myelin basic protein (MBP) (Chakraborty et al., 2001), and may stimulate myelination and post-injury remyelination through glutamate receptor signaling (de Rosa et al., 2019). In a mouse model for Canavan disease, impaired NAA catabolism results in reduced myelin formation and spongy white matter degeneration (Hoshino and Kubota 2014). Therefore, the function of ASPA in NAA catabolism is important for oligodendrocyte health.

Currently, no treatments exist for Canavan disease, making this a fatal disorder, but recent research has targeted dietary supplementation as well as viral gene delivery. Standard of care is largely palliative and includes gastrostomy tubes that deliver nutrients directly to the stomach, anti-seizure medications, and physical therapy to improve posture and to reduce pressure ulcers (Bokhari et al., 2022). Dietary supplementation with glyceryl triacetate has been proposed to address low acetate levels in Canavan disease. Though this was successful in a mouse model, it has yet to cause improvement in human patients (Segel et al., 2011). Recently, researchers engineered a modified AAV with a capsid that has preferential tropism toward oligodendrocytes to express ASPA. Intracerebroventricular injections of this virus in a mouse model of Canavan disease rescued ASPA activity and motor function (Francis et al., 2021). Thus, ASPA replacement therapy may be a feasible therapeutic approach for human patients.

## Cytoskeletal proteins

Cytoskeletal proteins, including microtubules, actin, septin, and intermediate filaments, form the cellular structure of glia. In oligodendrocytes, microtubules mediate long-distance transport and are found in processes that contact axons, as well as inside myelin sheaths (Weigel et al., 2021). Actin dynamics are important for the wrapping of myelin sheaths around axons (Nawaz et al., 2009; Zuchero et al., 2015). Septins also play an important role in oligodendrocyte development (Patzig et al., 2016). In astrocytes, the intermediate filament GFAP (glial fibrillary acidic protein) can play important roles in injury response and can be affected in leukodystrophy.

### Alexander disease (AxD)

AxD is a progressive demyelinating leukodystrophy. Children often present with symptoms (e.g., megalencephaly, limb stiffness or rigidity, seizures, and developmental delays) by 2 years of age and have an average life expectancy of 14–25 years (Prust et al., 2011). AxD is a rare disease with an estimated 5-years prevalence of one in 2.7 million in a study in Japan (Yoshida et al., 2011). AxD is caused by autosomal dominant mutations in the *GFAP* gene. To date, over 100 missense variants in coding regions are associated with AxD (Saito et al., 2018). However, most AxD mutations are not inherited, but, instead, are *de novo* mutations (Li et al., 2006).

Many lines of evidence indicate that protein aggregation is a profound component in the AxD pathology due to the striking accumulation of Rosenthal fibers consisting of GFAP. GFAP, an intermediate filament protein, is a major component of astrocytic processes and becomes upregulated in reactive astrocytes responding to injury (Wilhelmsson et al., 2004). Overexpression of human wildtype *GFAP* in mice recapitulated Rosenthal fiber pathology and was lethal (Messing et al., 1998), indicating that GFAP protein levels are important in AxD pathology. Recent research suggests that certain GFAP isoforms or GFAP containing PTMs may be more prone to aggregation. For example, minor isoforms of GFAP (e.g., delta and kappa) preferentially aggregate in Rosenthal fibers (Lin et al., 2021). In addition, severe AxD patients selectively contain GFAP phosphorylated at Ser13, which facilitates GFAP aggregation in patient-derived iPSCs (Battaglia et al., 2019). Thus, many considerations, such as mutations, isoform specificity, and PTMs, may affect GFAP aggregation in AxD.

Though most treatment options for AxD are based on symptom management, researchers are exploring new approaches, such as ASOs to decrease GFAP expression. This approach is supported by results showing that mice lacking GFAP have very mild defects and therefore decreasing GFAP protein levels is unlikely to produce a toxic loss of function phenotype. In experiments in a mutant *GFAP* (R236H) mouse line, ASOs targeted against the 3′UTR of *Gfap* were injected intracerebroventricularly. By 2 weeks after injection, this resulted in less *Gfap* mRNA, near elimination of GFAP protein, fewer Rosenthal fibers, and improved body condition scores (Hagemann et al., 2018). Thus, GFAP-targeting ASOs are a viable therapeutic approach for AxD and additional follow-up studies should be performed.

### Autosomal dominant leukodystrophy (ADLD) with autonomic disease

ADLD is an adult-onset leukodystrophy characterized by spasticity, ataxia, and autonomic issues, such as dysregulation of blood pressure and body temperature, and loss of bladder/bowel control (Zerbin-Rudin and Peiffer 1964; Eldridge et al., 1984). Patients display white matter lesions before the onset of symptoms, typically in the fifth decade, which progress over the course of 10–20 years (Finnsson et al., 2015). Most cases are autosomal dominant (Schuster et al., 2011) and caused by duplication of the *LMNB1* gene that encodes lamin B1 (Padiath et al., 2006; Brussino et al., 2009).

Lamins are intermediate filament proteins that form the mesh-like network of the nuclear lamina, which lines the inner nucleoplasmic side of the nuclear envelope. Mutations in lamin A can cause premature aging (progeria), muscular dystrophy, cardiomyopathy, and peripheral neuropathy (Shin and Worman 2022). Patient fibroblasts overexpress lamin B1, which leads to increased nuclear rigidity, misshapen nuclei, and possible changes in nuclear signaling (Ferrera et al., 2014). Furthermore, a glioma cell line overexpressing *LMNB1* also has misshapen nuclei with many aberrant layers of nuclear membranes (Ratti et al., 2021). Mouse models have shown that both cell-autonomous and non-cell-autonomous effects occur. Mice globally overexpressing lamin B1 show downregulation of PLP1 (Heng et al., 2013). Mice with oligodendrocyte-specific overexpression of lamin B1 show astrogliosis, microglia infiltration, decreased expression of genes that regulate lipid synthesis in oligodendrocytes, age-dependent demyelination (Rolyan et al., 2015), and motor dysfunction, but no autonomic phenotype (Lo Martire et al., 2018).

Recently, a drug screen in ADLD fibroblasts identified an inhibitor of heat shock protein 90 as a possible modulator of lamin B1 expression (Giorgio et al., 2021). Thus, therapeutic strategies for ADLD treatment are beginning to be identified.

### Hypomyelination with atrophy of the basal ganglia and cerebellum (H-ABC)

H-ABC is a leukodystrophy inherited in an autosomal dominant manner. Beginning in infancy to early childhood, patients present with movement and speech abnormalities, including ataxia and spasticity, and may never develop fine motor skills. MRI studies have revealed atrophy of the basal ganglia, putamen, and cerebellum, and histology studies have confirmed hypomyelination (van der Knaap et al., 2002; van der Knaap et al., 2007).

H-ABC is caused by mutations in the *TUBB4A* gene which codes for β-tubulin, a major structural protein that dimerizes with ⍺-tubulin to form heterodimers that are the building blocks of the microtubule cytoskeleton. Compared to other β-tubulin genes, *TUBB4A* is highly expressed by oligodendrocytes and its expression increases postnatally, consistent with a role in myelination (Zhang et al., 2014; Park et al., 2021). In oligodendrocytes, microtubules play important roles in building the structure of oligodendrocyte branches and myelin sheaths (Fu et al., 2019) as well as in transporting mRNA and other cargos (Carson et al., 1997; Herbert et al., 2017). Loss of the microtubule nucleation protein TPPP (tubulin polymerization promoting protein) results in shorter and thinner myelin sheaths as well as behavioral defects in motor coordination (Fu et al., 2019) and in innate and memory-dependent fear responses (Nguyen et al., 2020).

H-ABC can be caused by many different point mutations in *TUBB4A*. *TUBB4A* mutations can affect microtubule stability and growth, and interfere with motor protein binding to microtubules (Vulinovic et al., 2018). One example is a *de novo* point mutation (D249N) at a highly conserved aspartic acid residue that is located on the dimer interface between α-tubulin and β-tubulin and is important for heterodimer formation (Simons et al., 2013). Indeed, a mouse model of the D249N mutation recapitulates disease-like motor dysfunction and hypomyelination and indicates that both neurons and oligodendrocytes are affected. Cultured cerebellar granule neurons expressing this mutation displayed a variety of morphological defects, including shorter axons, fewer dendrites, and less dendritic branching. Similarly, in the differentiated oligodendrocyte-like cell line Oli-neu, expression of this mutation resulted in lack of complex branching morphology (Curiel et al., 2017). Furthermore, this mouse model also displays fewer mature oligodendrocytes, striatal neurons, and cerebellar granular neurons, as well as more caspase-3-positive apoptotic OPCs and cerebellar granular neurons (Sase et al., 2020).

There are currently no treatments for H-ABC, though rehabilitation and other management strategies are often utilized (Rossi et al., 2018). However, *Tubb4a* knockout mice do not exhibit any deleterious effects (Sase et al., 2020), indicating that H-ABC may be an ideal target for ASO therapy that aims to decrease expression of mutant *TUBB4A* mRNAs.

## Transcription defects

### 4H leukodystrophy

4H leukodystrophy is so named due to its alliterative primary clinical manifestations: hypomyelination, hypodontia, and hypogonadotropic hypogonadism (Bernard and Vanderver 1993). This leukodystrophy is also known as RNA polymerase III (*POLR3*)-related leukodystrophy due to mutations in *POLR3A*, *POLR3B*, and *POLR3K genes* (Bernard et al., 2011; Tétreault et al., 2011; Dorboz et al., 2018). The autosomal recessive inheritance pattern of 4H leukodystrophy requires biallelic expression of the pathogenic variant (e.g., homozygous or compound heterozygous mutations). Consistent with this inheritance pattern, a homozygous conditional knock-in mice expressing mutant *Polr3a* under the *Olig2* promoter displays hypomyelination as well as a range of cognitive, sensory, and sensorimotor defects (Merheb et al., 2021).

Despite the existence of a mouse model for 4H leukodystrophy, the mechanism for how *POLR3* mutations cause hypomyelination remains unclear. Multiple hypotheses have emerged in the literature. First, *POLR3* mutations may lead to tRNA deficits that impact global translation. Indeed, POLR3 transcribes more than 100 tRNA genes (Goffeau et al., 1996), and mutations in *POLR3* result in decreased tRNA transcription and increased tRNA post-translational modifications (Arimbasseri et al., 2015). In addition, oligodendrocytes may be more vulnerable to tRNA deficits due to their heavy reliance on local translation. For example, MBP is locally translated within the myelin sheath and many mRNAs are found locally in myelin fractions by RNA-seq (Thakurela et al., 2016; Herbert et al., 2017; Meservey, Topkar, and Fu 2021). Second, in a human oligodendrocyte-like cell line, *POLR3* mutation can impair production of a non-coding RNA (ncRNA) that may be important for myelin formation and expression of myelin transcripts like *MBP* mRNA (Choquet et al., 2019). However, it is not clear which downstream RNAs could be feasible therapeutic targets for restoring myelination. Thus, future research elucidating the cellular pathology of oligodendrocytes in 4H leukodystrophy models will be important for uncovering logical therapeutic approaches.

### *NKX6-2*-related spastic ataxia with hypomyelination (SPAX8)

*De novo* mutations in the *NKX6-2* gene are associated with spastic cerebellar ataxia that presents with hypomyelination on MRI. While neonatal-onset SPAX8 is associated with global delays, childhood-onset SPAX8 typically results in only motor delays (Chelban et al., 2017; 2020). NKX6-2 is a homeobox transcription factor that is important for oligodendrocyte differentiation and myelination in the hindbrain and spinal cord (Vallstedt, Klos, and Ericson 2005; Cai et al., 2010). Indeed, transcription factors NKX6-2, SOX10, and OLIG2 can be used to reprogram human fibroblasts into O4-positive OPC-like cells that can differentiate into MBP-positive oligodendrocyte-like cells (Ehrlich et al., 2017). Specifically, NKX6-2 can regulate expression of the microtubule severing protein stathmin and paranodal cell adhesion molecules neurofascin and contactin (Southwood et al., 2004), which highlight a role for NKX6-2 in regulating oligodendrocyte cytoskeleton and paranodal structures. Currently, no animal model for this disease exists, though one may prove useful to determine disease mechanisms and investigate treatments.

### Waardenburg-hirschsprung disease (WS4)

Mutations in the gene encoding the transcription factor SOX10 are associated with WS4 (Pingault et al., 1998), which is a subtype of a family of Waardenburg syndromes. WS4 is characterized by deafness, reduced pigmentation, skull and intestine abnormalities, and reduced myelin production by both oligodendrocytes and Schwann cells (Inoue et al., 1999; Inoue et al., 2002). In addition to its roles in the development of the colon and of melanocytes, SOX10 is important for the development of both CNS and PNS glia. *In vivo*, *SOX10*-null mice show impaired development of Schwann cells and other PNS glia (Britsch et al., 2001) and diminished oligodendrocyte differentiation in the CNS (Stolt et al., 2002).

Biochemical studies indicate that mutant SOX10 acquires a toxic gain of function. Though many *SOX10* mutations result in truncated mRNA transcripts, they are not downregulated by the nonsense-mediated decay pathway. In fact, mutant SOX10 proteins surprisingly outcompete wildtype SOX10 for DNA binding when co-expressed in glioblastoma cells (Inoue et al., 2004). Thus, this dominant-negative mechanism indicates that WS4 may be best therapeutically targeted by decreasing mutant *SOX10* mRNA expression or decreasing mutant SOX10 protein activity.

## Translation machinery defects

### tRNA synthetase-related leukodystrophies

Mutations in genes that encode components of the tRNA multisynthetase complex (DARS1, RARS1, EPRS1) are implicated in hypomyelination. These mutations cause a spectrum of clinical phenotypes, from severe seizures and brain atrophy within the first 3 months of age, to mild ataxia and cognitive deficits beginning at 1 year old or later (Taft et al., 2013; Mendes et al., 2020).

tRNA synthetases function to join the proper amino acid to each tRNA molecule. Though it is unclear how mutations in this mechanism cause hypomyelination, studies have shown reduced EPRS protein levels and activity in patients with *EPRS* mutations (Mendes et al., 2018). For *RARS*, the most common mutation is associated with mild disease, while truncation mutations and mutations located in the enzymatic domain are associated with severe disease (Mendes et al., 2020). These findings suggest that disruption in tRNA complex assembly, reduced tRNA synthetase activity, and translation disruption result in insufficient myelination in the developing brain.

Researchers have questioned whether tRNA synthetase mutations cause hypomyelination via oligodendrocyte dysfunction or neuron dysfunction (Mendes et al., 2020; Wolf, ffrench-Constant et al., 2018). Indeed, mutations in tRNA synthetase genes *VARS* (Friedman et al., 2019; Siekierska et al., 2019) and *AIMP2* (Shukla et al., 2018) have been implicated in neuronal disorders with shared phenotypes in severely affected patients, such as cerebral atrophy and seizures. Thus, more research to dissect the cell-specific effects of mutations in tRNA synthetase machinery is important for better mechanistic understanding of these leukodystrophies.

### Vanishing white matter (VWM) disease or childhood ataxia and cerebral hypomyelination (CACH)

VWM disease, also known as CACH, is one of the more prevalent leukodystrophies, with an estimated incidence of one in 80,000–100,000 live births (van der Knaap et al., 2022). VWM is highly variable in age of onset, severity, and progression. In some cases, children exhibit normal early development, whereas others experience ataxia, and delayed speech and cognitive milestones (Schiffmann et al., 1994; van der Knaap et al., 2003). MRI imaging of VWM patients shows distinct patterns of diffuse white matter signal (Schiffmann et al., 1994; van der Knaap et al., 1997). These signs of hypomyelination are present even prior to symptom onset (Schiffmann et al., 2003). As white matter MRI signals decrease and white matter tract deterioration progresses, neurological symptoms worsen. Unfortunately, this hypomyelination can become so severe that it can result in death around the first or second decade of life (van der Knaap et al., 2022).

A number of studies in postmortem brains have described the histopathological characteristics of VWM. First, one study observed oligodendrocytes with “foamy” cytoplasm, which was hypothesized to indicate abnormal glycosylation (Fogli et al., 2002). Second, a thorough literature review of VWM brain pathology studies highlighted a pattern of disproportionately high number of OPCs compared to mature oligodendrocytes. Lastly, oligodendrocytes in VWM brains have abnormal mitochondrial morphology (Wong et al., 2000), and increased rates of apoptosis (Brück et al., 2001; Van Haren et al., 2004). Thus, these histology results indicate that oligodendrocytes in VWM have maturation defects and cell health issues.

Genetics studies of VWM have revealed mutations in the genes encoding the five subunits of the eukaryotic initiation factor 2B (*EIF2B1, EIF2B2, EIF2B3, EIF2B4, and EIF2B5*). EIF2B regulates the first stage of translation initiation: the recruitment of ribosomes and proper identification of the start codon (Bogorad et al., 2018). While mutations can occur in any of the five subunits, 80% are missense mutations occuring in gene encoding the ε-subunit (*EIF2B5*) (van der Knaap et al., 2002). Since eIF2B is a guanine nucleotide exchange factor, *EIF2B* mutants display reduced GTP hydrolysis due to conformational changes, and therefore may adversely impact global mRNA translation (Fogli et al., 2004). These changes can trigger downstream activation of the integrated stress response, a system that regulates protein translation and folding in response to both intracellular and extracellular stress (van der Knaap et al., 2022).

Studies to elucidate the cellular mechanisms of VWM have uncovered both cell-autonomous and non-cell-autonomous mechanisms. Mutant-*Eif2b5* mice exhibit delayed white matter development (Geva et al., 2010), and purified OPCs cultured from these mice had mitochondrial defects, as well as impaired differentiation and morphology (Herrero, Mandelboum, and Elroy-Stein 2019). However, in a co-culture system using glial cells isolated from wildtype and mutant-*Eif2b5* mice, mutant astrocytes secreted factors that inhibited wildtype OPCs from differentiating into mature oligodendrocytes (Dooves et al., 2016). Thus hypomyelination in VWM likely involves defects in both OPCs and astrocytes.

Although there are no treatment options available for VWM, a recent study performed an ambitious small molecule drug screen. The authors conducted a screen of 2400 FDA-approved drugs using iPSC-derived astrocytes from a patient harboring mutations in two subunits of eIF2B. They identified 113 anti-inflammatory drugs with cytoprotective effects, including ursodiol, berberine, deflazacort, and zileuton. Of these, ursodiol rescued oxidative stress and mitochondrial dysfunction in VWM patient iPSC-derived astrocytes (Ng et al., 2020). However, for all the drugs identified, further *in vivo* animal testing is needed prior to moving to any clinical studies.

## Cell junction defects

### Megalencephalic leukoencephalopathy with subcortical cysts (MLC)

MLC is an autosomal-recessive vacuolating leukodystrophy that is also known as van der Knaap disease, after the pediatric neurologist who first described the condition (Bosch and Estévez 2021). MLC is characterized by prominent macrocephaly that progressively develops within the first year of life (van der Knaap et al., 2012). Due to the macrocephaly, children exhibit difficulty walking, while gradual onset of ataxia and spasticity further contribute to gross motor delays and difficulties. Typically, children have normal cognition, though mild mental deterioration can occur later during development. Computed tomography (CT) and MRI scans revealed that MLC patients have mild-to-severe swelling or edema, vacuolization of white matter, and subcortical cysts (van der Knaap et al., 1995; van der Knaap et al., 2002; Dash, Raj, and Sahu 2015).

MLC can be categorized into different subtypes based on the specific genes mutated. MLC1 subtype results from a missense mutation in the *MLC1* gene. *Mlc1* mRNA is expressed exclusively by astrocytes, but not by oligodendrocytes (Schmitt et al., 2003; Dubey et al., 2015). *MLC1* encodes a transmembrane protein (Leegwater et al., 2001) that participates in osmoregulation by astrocytes. In astrocytoma cells, wildtype MLC1 localizes to the plasma membrane in a complex with aquaporin, the potassium channel Kir4.1, and the TRPV4 channel. By contrast, mutant MLC1 localizes intracellularly and this disrupts osmolarity homeostasis (Lanciotti et al., 2012). Consistent with this, a study in HeLa cells demonstrated that mutant MLC1 protein was retained in the ER (Duarri et al., 2008), likely due to protein misfolding. Both mouse and zebrafish models recapitulate human phenotypes, including macrocephaly and edema (Sirisi et al., 2014). A *Mlc1*-null mouse model unveiled morphologically abnormal astrocytes present in the swollen white matter. These astrocytes express lower levels of the adhesion molecule GLIALCAM, which is also known as HEPACAM (hepatocyte cell adhesion molecule). No neuronal or oligodendrocyte defects were observed, because MLC-null axons were fully myelinated in EMs and oligodendrocyte-specific transcripts were expressed at normal levels (Dubey et al., 2015).

MLC2a subtype is caused by mutations in the *HEPACAM* gene, which encodes a cell adhesion molecule in the immunoglobulin family (Sirisi et al., 2014). *Hepacam* mRNA is present in both astrocytes and oligodendrocytes (Hoegg-Beiler et al., 2014; Bugiani et al., 2017). Recent work indicates that HEPACAM plays a crucial role in gap junction coupling between adjacent astrocytes (Baldwin et al., 2021). Mutations in this gene have been shown to cause protein mislocalization (Elorza-Vidal et al., 2020).

MLC1 and HEPACAM likely function in a complex to mediate cell-cell interactions. In mouse models, MLC1 and HEPACAM mutually affect each other’s localization. Knockout of *Hepacam* affects *Mlc1* expression and MLC protein localization (Hoegg-Beiler et al., 2014; Bugiani et al., 2017), while knockout of *Mlc1* decreases HEPACAM levels and leads to its mislocalization (Dubey et al., 2015). A recent study showed that astrocytes in *Mlc1* knockout mice are more branched, but their endfeet make fewer contacts with vasculature. In addition, *Mlc1* knockout mice have high incidence of neurons aberrantly contacting vasculature (Gilbert et al., 2021). These defects in gliovascular coupling may underlie the fluid buildup and macrocephaly observed in MLC patients (van der Knaap et al., 2012; Dubey et al., 2015).

Although there are no treatments for MLC, recent work has provided some preclinical insight into viral replacement therapy. Using *Mlc1* knockout mice, researchers demonstrated that cerebellar subarachnoid administration of an AAV expressing human *MLC1* under a GFAP promoter increased MLC1 protein expression in the cerebellum and decreased white matter vacuolation in a dose-dependent manner (Bosch and Estévez 2021). This study indicates that replacement therapy is a feasible therapeutic approach for MLC.

### Oculodentodigital dysplasia (ODDD)

ODDD is a multisystem syndrome whose symptoms present in three major categories: ocular, dental, and digital. Ocular symptoms include very small corneas (microcornea), eyes that do not look in the same direction (strabismus), and fluid buildup in the eye (glaucoma). Dental symptoms include abnormally small teeth, defective enamel, and a thickened lower jaw. Digital symptoms usually present as webbing between the fourth and fifth fingers and third, fourth, and fifth toes (syndactyly) (Traboulsi, Faris, and Der Kaloustian 1986). The neurological symptoms of ODDD are wide-ranging and include lower limb spasticity or paralysis (paraparesis), ataxia, bladder disturbances, and seizures. MRI imaging has shown decreased white matter intensity in the CNS, which likely contributes to motor symptoms in ODDD (Loddenkemper et al., 2002).

ODDD is caused by mutations in *GJA1* (also known as *Cx43*), which encodes the gap junction α-1 protein (connexin 43). Mutations causing ODDD are typically inherited in an autosomal-dominant fashion (Paznekas et al., 2003). One screen of 17 families found 16 unique missense mutations and one codon duplication; these mutations were present only in affected individuals, indicating an autosomal-dominant pattern of inheritance (Paznekas et al., 2003). However, there are also documented cases of autosomal-recessive inheritance (Joss et al., 2008) and novel *de novo* mutations (Choi et al., 2018; Pace et al., 2019). Thus, ODDD displays a variety of inheritance patterns.

Cx43 is one protein component comprising gap junctions between astrocytes and other glial cells (Orthmann-Murphy et al., 2007). A mouse model deficient in the *Cx43* and *Cx30* genes had a complete loss of astrocyte–astrocyte gap junctions, which reduced the rate of potassium clearance and increased the probability of seizure-like activity in hippocampal neurons *in vitro* (Wallraff et al., 2006). Cx43 has also been shown to be involved in astrocyte–oligodendrocyte gap junctions (Nagy et al., 2003). Double knockout mice with astrocyte-specific deletion of *Cx43* and global deletion of *Cx30* displayed demyelination in multiple regions (corpus callosum, cerebellum, corticospinal and other tracts) and edema within myelin. These mice also performed worse on the rotarod motor coordination assay, which is consistent with ODDD neurological motor symptoms (Lutz et al., 2009).

Treatments for ODDD are symptomatic and supportive. For instance, an eye patch over the stronger eye can correct strabismus, corrective surgery can address defects in the fingers and toes, and antispastic medication can ameliorate neurological symptoms (Shinya et al., 2021). Thus, additional basic research is needed to elucidate the role of gap junctions in astrocytes and oligodendrocytes.

### Connexin-47-related Pelizaeus-Merzbacher-like disease (PMLD)

PMLD presents with similar symptoms as Pelizaeus–Merzbacher Disease (PMD), including nystagmus, spasticity, head tremors, poor muscle tone, and progressive spasticity, but PMLD symptoms are less severe than PMD symptoms. PMLD is autosomal recessive and associated with mutations in connexin 47 (Cx47), which also known as connexin 46.6, gap junction protein γ2 (GJC2), and gap junction protein α12 (GJA12) (Uhlenberg et al., 2004; Bilir et al., 2013; Abrams et al., 2014; Owczarek-Lipska et al., 2019). Many patients present with biallelic (compound heterozygous) mutations in combination (Owczarek-Lipska et al., 2019).

Whether mutant Cx47 causes leukodystrophy via loss or gain of function remains unclear. In one study that treated HeLa cells with mutant Cx47, gap junction formation was normal, but electrical coupling was disrupted (Abrams et al., 2014). In a bulk RNA-seq transcriptome, Cx47 is expressed very highly and specifically in oligodendrocytes (Zhang et al., 2014). However, a *Cx47* knockout mouse did not display any clinical symptoms (Odermatt et al., 2003), which hints that *Cx47* mutations in PMLD patients may exert a toxic gain-of-function effect. Thus, elucidation of PMLD disease mechanisms is needed to lay the important groundwork for the road toward translational studies.

## Myelin structural protein defects

Proteomic analysis of human myelin shows that the protein composition of myelin largely consists of two proteins - PLP1 (45%) and MBP (28%). Additional myelin proteins include CNP (4.5%), MOG (0.8%), MAG (0.3%), MOBP (0.1%), and others (Gargareta et al., 2022). Many of these proteins play a role in compaction, the formation of efficient myelin insulation by bringing together adjacent layers of membrane. Compaction happens both intracellularly (between membranes from the same wrap) and extracellularly (between membranes from different but juxtaposed wraps). While intracellular compaction largely relies on MBP, extracellular compaction relies on PLP. Additional transmembrane proteins, like MAG and MOG, are found in non-compact regions of the myelin sheath (Raasakka and Kursula 2020).

### *CNP*-related hypomyelinating leukodystrophy

A recent study of multiple members of a consanguineous family found a homozygous mutation in the *CNP* (2′,3′-cyclic nucleotide-3′-phosphodiesterase) gene. Symptoms began ∼1 year of age and include arrest then regression in motor skill development, dystonia, irritability, microcephaly, scoliosis and hirsutism. MRI imaging showed hypomyelination, especially of periventricular and subcortical white matter. Ultimately these children passed away ∼five to eight years of age. This mutant CNP is unstable and has barely detectable protein levels by Western blot of patient fibroblast lysates (Al-Abdi et al., 2020). CNP plays an important role in establishment of the cytoplasmic channels in myelin sheaths that allow transport between outer to inner layers of myelin (Snaidero et al., 2017).

### *MAG*-related PMLD

Another gene associated with PMLD is *MAG* (myelin-associated glycoprotein). Three siblings from a consanguineous family presented with infantile-onset PMLD that later in adulthood developed into additional symptoms of mental retardation, dysarthria, optic atrophy, and peripheral neuropathy. They share a homozygous missense mutation in *MAG* that results in mutant MAG retention in the ER, misshapen paranodal junctions, and thin myelin sheaths (Lossos et al., 2015).

### *MAL*-related leukodystrophy

A recent report found that a patient presenting with PMLD had a novel missense mutation (A109D) in the gene encoding myelin and lymphocyte protein (MAL) (Elpidorou et al., 2022). MAL is a four-pass transmembrane protein that is important for the structure of paranodal loops, which are located at the ends of myelin sheaths and flank the nodes of Ranvier. In *Mal* knockout mice, paranodal loops are aberrantly oriented facing away from the axon (Schaeren-Wimers et al., 2004). Experiments in cultured kidney cells demonstrated that mutant MAL aggregates in the ER, likely due to a protein misfolding defect. Treatment with 4-phenylbutyrate, a chemical chaperone, appeared to restore MAL trafficking out of the ER (Elpidorou et al., 2022), which supports targeting protein misfolding as a putative therapeutic strategy for this leukodystrophy.

### Pelizaeus–Merzbacher disease (PMD)

PMD is an X-linked, dysmyelinating leukodystrophy characterized by involuntary eye movement, head tremors, and systematically poor muscle tone. Symptom onset usually occurs within the first year of age (Laukka et al., 2016). PMD is caused by mutations in the gene encoding PLP1, one of the most abundant membrane-associated myelin proteins in the CNS. Many types of *PLP1* mutations can cause PMD. These include duplications (Inoue et al., 1996), deletions (Raskind et al., 1991), and missense mutations. Of these, *PLP1* gene duplication is the most common cause of PMD (Sistermans et al., 1998; Mimault et al., 1999), while missense point mutations in *PLP1* also result in axonal degeneration in the CNS (Garbern et al., 2002).

Both loss-of-function and gain-of-function mechanisms have been suggested for how *PLP1* mutations cause PMD. Early neuropathology studies of PMD brains revealed significant deficiencies in myelination as well as decreased levels of myelin proteins (MBP, MAG, and CNP) and lipids (cerebroside, sulfatides, and sphingomyelin) (Bourre et al., 1978). As a four-pass transmembrane protein, PLP1 functions in myelin compaction, bringing together adjacent extracellular layers of myelin. Indeed, *PLP1*-null mice exhibit myelin structural defects, such as incomplete compaction, and axonal accumulation of organelles (Klugmann et al., 1997; Edgar et al., 2004), which is indicative of axonal transport defects. Additional studies suggest that PMD mutations may lead to toxic gain of function. In patient-derived iPSCs differentiated into oligodendrocytes, mutant PLP1 proteins misfold and mislocalize to the ER (Numasawa-Kuroiwa et al., 2014) and can contribute to iron toxicity. These mechanisms may contribute to oligodendrocyte cell death and iron chelation therapy has even been suggested to prevent cell death (Nobuta et al., 2019). Thus, determining the disease-causing mechanisms for different PLP1 mutations will be crucial for assessing therapeutic rationales for different patient cohorts.

Recently, promising experiments in mice indicate that ASOs may be a feasible therapeutic approach for PMD. First, researchers ensured that decreasing PLP1 levels does not have detrimental effects. The *jimpy* mouse model expresses a truncated defective copy of PLP1 and shares similar cellular and neurological phenotypes with severe PMD patients. Interestingly, targeted germline deletion of the *PLP1* gene improved the lifespan of this mouse and eliminated tremors, ataxia, and seizures. Thus, to recapitulate this positive effect, ASOs targeting *Plp1* mRNA were administered to *jimpy* mice. This resulted in silencing of *Plp1*, restored white matter volume, and astonishingly improved survival rate (Elitt et al., 2020). However, it should be noted that *jimpy* is a spontaneously occurring *PLP1* mutation in mice and does not directly model PMD mutations. Moreover, *PLP1*-null adult mice have myelin and axon defects (Klugmann et al., 1997; Edgar et al., 2004), indicating that complete loss of PLP1 can also be deleterious. Thus, it will be important for future research to focus on long-term effects of administering *Plp1*-targeted ASOs.

Another possible cause of PMD pathology is mutations causing aberrant splicing. *Plp* mRNA can be spliced into two isoforms: *Plp1* and the shorter *Dm20* (Nave et al., 1987). Some PMD patients have mutations in *PLP1* Intron 3, which contains an alternative splicing site, (Taube et al., 2014; Kevelam et al., 2015). These mutations alter alternative splicing and disrupt the ratio of PLP1/DM20 protein in oligodendrocyte cultures (Hobson et al., 2006; Taube et al., 2014). In an attempt to target disease-causing mutations, morpholino oligomers blocking the DM20 splice site successfully shifted alternative splicing towards PLP1 in oligodendrocyte culture. In addition, intracerebroventricular delivery of the same oligomer to neonatal mice modeling this mutation rescued both *Plp* mRNA and PLP protein levels (Tantzer et al., 2018). Thus, approaches targeting both PLP overexpression and splicing defects are being developed and show promising results in animal models.

## Other transmembrane protein defects

### *CNTNAP1*-related arthrogryposis and leukodystrophy

Two children presenting with arthrogryposis (joint stiffness and muscle weakness) and hypomyelination by MRI were found to have either homozygous or compound heterozygous mutations in the *CNTNAP1* gene, which encodes contactin-associated protein 1 (Caspr1). Caspr1 is a paranodal cell-adhesion protein in the axon that interacts with neurofascin 155 to form paranodal junctions. EM of sural nerve biopsies showed a striking absence of paranodal loop structures (Conant et al., 2018).

### Hypomyelination with congenital cataracts (HCC)

HCC is characterized by the appearance of cataracts within the first year of life, followed by progressive neurological and cognitive impairment, and hypomyelination in the CNS and PNS (Biancheri et al., 2011; Wolf et al., 2021). HCC is caused by mutations in *FAM126A* gene that encodes the membrane protein hyccin, which is expressed ubiquitously in the adult brain and also in the lens of the eye (Zara et al., 2006).

Hyccin function may be important for myelin sheath development. Hyccin is a component of the PI4K (phosphatidylinositol 4-kinase) complex that synthesizes phosphatidylinositol 4-phosphate (PI4P). Absence of hyccin destabilizes the PI4K complex (Baskin et al., 2016), which may result in fewer PI4P and other phosphoinositides intermediates that are important for myelin formation. Indeed, phosphoinositides are important both for the association of MBP with adjacent myelin sheath plasma membranes (Nawaz et al., 2009; Zuchero et al., 2015), and for opening cytoplasmic channels that are found from outer to inner myelin sheath layers as they grow and wrap around the axon (Snaidero et al., 2014). More research on how hyccin mutations affect myelination are needed in order to better design novel therapeutic treatments for this leukodystrophy.

### *SLC35B2*-related chondrodysplasia with hypomyelinating leukodystrophy

Homozygous mutations in *SLC35B2* were found in two patients presenting with chondrodysplasia (disrupted growth of cartilage), intellectual disability, and hypomyelination on brain MRI (Guasto et al., 2022). The *SLC35B2* gene (also known as *PAPST1*) encodes a 3′-phosphoadenosine 5′-phosphosulfate (PAPS) transporter that moves PAPS from the cytoplasm into the Golgi apparatus. In the Golgi, sulfotransferases add PAPS to proteins already modified with GAGs (glycosaminoglycan chains). This process generates sulfated proteoglycans (PGs), which are important in the extracellular matrix for building and maintaining connective tissues in bones, cartilage, and even brains (Schwartz and Domowicz, 2018). Biochemical analyses of sulfated proteins in both patient fibroblasts and serum indicate reductions in protein sulfation (Guasto et al., 2022). Moreover, in a transfected kidney cell line, mutant SLC35B2 lost its Golgi-specific localization and instead appeared throughout the cell. These results suggest that mutant SLC35B2 may act through a loss-of-function mechanism. Though the role of PAPS transporters in myelination is still unclear, the discovery of these mutations open the door toward exciting future research.

### Transient infantile hypomyelinating leukodystrophy-19 (HLD19)

Recently, heterozygous missense mutations in the transmembrane protein 63A (*TMEM63A*) gene were identified in a hypomyelinating leukodystrophy that usually presents in the first weeks of life with nystagmus, neurological abnormalities, and hypomyelination by MRI. Surprisingly, all patients showed neurological improvement and nearly recovered hypomyelination by 4 years of age. TMEM63A is a mechanosensitive ion channel (Yan et al., 2019) that is preferentially expressed in oligodendrocytes and microglia (Zhang et al., 2014). While cultured cells expressing wildtype TMEM63A exhibited stretch-activated currents upon mechanical stimulation, cells expressing mutant TMEM63A did not (Yan et al., 2019). Though the mechanism leading to leukodystrophy is yet unknown, this discovery highlights an interesting finding that mechanosensitive ion channels play a role in myelination.

### *TMEM106B*-related hypomyelinating leukodystrophy

Recently, a hypomyelinating leukodystrophy with relatively mild phenotypes was attributed to a single *de novo* mutation in the gene encoding transmembrane protein 106B (*TMEM106B*) leading to a D252N residue change. In 4 unrelated patients, symptoms include nystagmus, delays in motor skills development, and intellectual impairment (Simons et al., 2017).

Multiple studies support a role for TMEM106B in regulating both lysosomal function and acidification as well as myelin sheath structure. First, TMEM106B binds to the lysosomal protein vacuolar-ATPase accessory protein 1 (AP1). Loss of TMEM106B leads to fewer lysosomes in cultured neurons and decreased levels of lysosomal enzymes in mice (Klein et al., 2017). Second, early studies indicate that late endosomes and lysosomes transport the transmembrane protein PLP to the myelin sheath (Trajkovic et al., 2006). Indeed, *Tmem106b* knockout mice have reduced PLP and MOG levels by Western blot and their myelin sheaths contain vacuoles in between myelin layers visible by EM (Feng et al., 2020). Furthermore, *Tmem106b* knockout mice are more susceptible to cuprizone-induced demyelination damage and have poorer ability to remyelinate. Primary oligodendrocytes cultured from *Tmem106b* knockout mice contain clusters of PLP-positive lysosomes inside the cell body and decreased distribution of PLP outside of the cell body (Zhou et al., 2020). Together, these experiments indicate that TMEM106B dysfunction can lead to lysosomal defects in neurons as well as aberrant vacuoles and PLP trafficking defects in the myelin sheath.

Interestingly, TMEM106B dysfunction is also implicated in neurodegenerative diseases in elderly patients. Polymorphisms of *TMEM106B* were identified as a risk factor for frontotemporal degeneration (FTD) (Van Deerlin et al., 2010). Very recently, TMEM106B amyloid filaments were discovered in the brains of older patients with various neurodegenerative diseases, including tauopathies, synucleinopathies, Aβ-amyloidoses and TDP-43 proteinopathies. Similar TMEM106B filaments were also found in older but neurologically normal patients, indicating that TMEM106B is prone to forming filaments in aging brains. The D252N leukodystrophy mutation (Simons et al., 2017) resides within the amyloidogenic region in TMEM106B (residues 120–254) (Schweighauser et al., 2022), but it is unclear whether the D252N mutation could be more amyloidogenic than wildtype TMEM106B. Surprisingly, polymorphisms in other leukodystrophy genes, including *ARSA* and *CYP27A1*, are also linked to FTD (Lok and Kwok 2021). Thus, these connections between FTD and leukodystrophies hint at common mechanisms and an interesting topic for future research.

### *TMEM163*-related hypomyelinating leukodystrophy

Recently, two patients with hypomyelinating leukodystrophy were found to carry two unique *de novo* variants of *TMEM163* (Yan et al., 2022). Both patients presented initially with symptoms consistent with PMLD, such as nystagmus, motor deficits, and hypomyelination on MRI. Similarly to transient infantile HLD19, hypomyelination on MRI improved with age.

TMEM163 is a small protein with six predicted transmembrane domains. It is a member of the ZNT/SLC30 zinc efflux transporter family (Sanchez et al., 2019). In cultured cells exposed to the zinc indicator Newport Green (NPG), expression of wildtype TMEM163 leads to decreased NPG fluorescence, but expression of mutant L76P TMEM163 leads to even lower NPG signal, indicating that mutant TMEM163 has hyperactive zinc efflux activity. Zebrafish in which *tmem163a* and *b* were knocked down and replaced with mutant human *TMEM163* had locomotion defects and decreased myelin protein expression (Yan et al., 2022), which recapitulates the motor deficits and hypomyelination seen in patients. These mutations raise the interesting hypothesis that dysregulation of zinc homeostasis can preferentially disrupt myelination. Consequently, further research should investigate how zinc efflux affects myelin growth and how this process is affected by these novel mutations.

## Immune system activation

### Aicardi-Goutieres syndrome (AGS)

AGS is an autosomal-recessive disorder that mainly affects the brain and the immune system. In the brain, there is a loss of white matter and the appearance of abnormal calcium deposits. Together, hypomyelination and calcification lead to severe disabilities, including joint and muscle rigidity, involuntary muscle contractions, and poor muscle tone. In the immune system, one case report found lymphocytes in the CSF, a 25-fold increase in CSF interferon α (IFN⍺) levels, and autoantibodies against phospholipids (Olivieri et al., 2013).

AGS is linked to mutations in genes encoding intracellular nucleic acid–sensor machinery, including *ADAR1, IFIH1, RNASEH2A, RNASEH2B, RNASEH2C, SAMHD1,* and *TREX1* (Crow 2013; Livingston and Crow 2016). Nucleic-acid sensors play an integral role in the innate immune response by inducing apoptosis, necroptosis, and pyroptosis when foreign or defective DNA and RNA are detected (Maelfait, Liverpool, and Rehwinkel 2020). Once these sensors are activated, they initiate induction of cytokines, leading to the transcriptional upregulation of type I interferons (McNab et al., 2015). AGS mutations lead to the accumulation of unusual double-stranded DNA species, which triggers chronic type I interferon release by astrocytes, and ultimately leads to autoimmunity. Indeed, elevated IFN⍺ level is a clinical marker for AGS (Crow et al., 2006).

Astrocytic release of type I interferon, specifically IFN⍺, may cause hypomyelination via multiple mechanisms. High levels of IFN⍺ adversely affect astrocytes, which may ultimately lead to insufficient trophic support for oligodendrocytes. Indeed, AGS patients have low astrocyte cell counts and chronic IFN⍺ exposure has deleterious effects on astrocytes, including reduced proliferation, increased reactivity, and gene dysregulation (Sase et al., 2018). Chronic IFN⍺ levels in AGS may also lead to hypomyelination by directly causing oligodendrocyte death. The type II interferon, interferon γ (IFNγ), harms cultured primary (*in vitro*) and *in vivo* oligodendrocytes by disrupting cellular bioenergetics pathways, such as glycolysis and mitochondrial respiration (Minchenberg and Massa 2019). However, currently there are no studies investigating the direct link between IFN⍺ and oligodendrocyte death (de Waard and Bugiani 2020).

The rarity of AGS presents difficulties in recruitment for clinical studies; however, anecdotal reports suggest that the kinase inhibitor Ruxolitinib may result in clinical improvements (Crow, Shetty, and Livingston 2020). Clearly, more basic research on cellular nucleic acid–sensor machinery and translation studies are needed in order to identify therapeutic targets in AGS.

### Hereditary diffuse leukoencephalopathy with spheroids (HDLS)

HDLS is an adult-onset disorder typically beginning in the fourth decade of life; therefore, it likely represents a demyelinating disease rather than a developmental disease. HDLS is characterized by various neurological (seizures and parkinsonism), psychiatric (personality changes and dementia), and somatic symptoms (arthritis and gastrointestinal pain). Histopathological studies have found demyelination in the cerebral cortex, axonal spheroids containing aggregates of phosphorylated neurofilament and amyloid precursor protein, and oxidative damage in glia (Axelsson et al., 1984; Baba et al., 2006; Ali et al., 2007). HDLS is an autosomal-dominant disorder and whole-exome sequencing revealed that it is caused by mutations in the gene encoding colony stimulating factor 1 receptor (CSF1R) (Rademakers et al., 2011).

Multiple sources of evidence indicate that microglia defects cause HDLS. CSF1R is the receptor for the cytokine CSF-1, which regulates the proliferation and differentiation of microglia in the CNS (Stanley et al., 1997). Upon ligand binding, wildtype CSF1R homodimerizes and auto-phosphorylates tyrosines in its cytoplasmic domain, but mutant HDLS CSF1R does not (Rademakers et al., 2011). Moreover, a mouse model with reduced CSF1R expression in microglia displays memory deficits, microglial activation, and demyelination (Biundo et al., 2021). Thus, CSF1R inactivating mutations likely contribute to HDLS myelin pathology through microglia.

Though no treatment currently exists, microglia replacement therapy has been proposed for HDLS. This would involve microglia depletion followed by replacement with either HSCs or engineered microglia expressing wildtype CSF1R (Han et al., 2020). Indeed *Csf1r* knockout mice can be partially rescued by bone marrow transplants and donor-derived cells engraft into the brain and adopt signature microglial genes (Bennett et al., 2018). Though more validation of this approach is needed, it presents a promising avenue for treatment of this leukodystrophy.

## Conclusion

The field of leukodystrophies is exemplary in identifying underlying genetic mutations. Perhaps due to decreased sequencing costs, many novel mutations are being discovered each year. This wealth of genetic information has provided not only a rich foundation for understanding the mechanistic underpinnings of leukodystrophies, but also insights into normal oligodendrocyte and myelin development, astrocyte cell biology, as well as glia–glia and glia–neuron interactions. Cellular mechanisms attributed to hypomyelination in leukodystrophies include contributions to lipid metabolism and myelin biogenesis by various organelles, such as lysosomes, peroxisomes, and mitochondria, as well as enzymes like ASPA. In addition, astrocytes may contribute to etiologies in leukodystrophies such as AxD, VWM, and AGS.

Selection of therapeutic strategies in translational studies of leukodystrophy have relied on basic research results that determined mode of genetic inheritance as well as cellular mechanisms. For autosomal recessive and X-linked leukodystrophies, replacement therapies and other therapies targeted at restoring loss of function have been validated for certain disorders. For autosomal dominant leukodystrophies, therapies aim to decrease expression of mutant or overexpressed proteins. ASOs, a new generation of neurotherapeutic that binds to RNA targeting sequences in order to achieve gene silencing, hold great promise. ASOs have been FDA-approved and are successful in targeting other genetic neurological diseases like SMA. Such applications would require careful validation in cell and animal models, but having a strong base of cellular targets and mechanisms is an important first step.

Future clinical studies in humans may face multiple challenges, including adverse or toxic effects, reproducibility in humans, and small sample sizes in rarer leukodystrophies. However, a solid foundation of many translational studies will hopefully lead to promising therapeutics approaches that may 1 day help patients affected by leukodystrophies.

## References

[B1] Abrams C. K. Scherer S. S. Flores-Obando R. Freidin M. M. Wong S. Lamantea E. (2014). A new mutation in GJC2 associated with subclinical leukodystrophy. J. Neurol. 261 (10), 1929–1938. 10.1007/s00415-014-7429-1 25059390 PMC4301586

[B2] Aerts J. M. Groener J. E. Kuiper S. Donker-Koopman W. E. Strijland A. Ottenhoff R. (2008). Elevated globotriaosylsphingosine is a hallmark of Fabry disease. Proc. Natl. Acad. Sci. U. S. A. 105 (8), 2812–2817. 10.1073/pnas.0712309105 18287059 PMC2268542

[B3] Al-Abdi L. Al Murshedi F. Elmanzalawy A. Al Habsi A. Helaby R. Ganesh A. (2020). CNP deficiency causes severe hypomyelinating leukodystrophy in humans. Hum. Genet. 139 (5), 615–622. 10.1007/s00439-020-02144-4 32128616

[B4] Aldenhoven M. Kurtzberg J. (2015). Cord blood is the optimal graft source for the treatment of pediatric patients with lysosomal storage diseases: Clinical outcomes and future directions. Cytotherapy 17 (6), 765–774. 10.1016/j.jcyt.2015.03.609 25840940

[B5] Ali Z. S. Van Der Voorn J. P. Powers J. M. (2007). A comparative morphologic analysis of adult onset leukodystrophy with neuroaxonal spheroids and pigmented glia-a role for oxidative damage. J. Neuropathol. Exp. Neurol. 66 (7), 660–672. 10.1097/nen.0b013e3180986247 17620991

[B6] Amado D. A. Davidson B. L. (2021). Gene therapy for ALS: A review. Mol. Ther. 29 (12), 3345–3358. 10.1016/j.ymthe.2021.04.008 33839324 PMC8636154

[B7] Arimbasseri A. G. Blewett N. H. Iben J. R. Lamichhane T. N. Cherkasova V. Hafner M. (2015). RNA polymerase III output is functionally linked to tRNA dimethyl-G26 modification. PLoS Genet. 11 (12), e1005671. 10.1371/journal.pgen.1005671 26720005 PMC4697793

[B8] Aubourg P. Sevin C. Cartier N. (2011). “Mouse models of metachromatic leukodystrophy and adrenoleukodystrophy,” in *Animal Models of Dementia. Neuromethods* ed. De Deyn P. Van Dam D. (Totowa, NJ: Humana Press), Vol. 48. 10.1007/978-1-60761-898-0_26

[B9] Axelsson R. Röyttä M. Sourander P. Akesson H. O. Andersen O. (1984). Hereditary diffuse leucoencephalopathy with spheroids. Acta Psychiatr. Scand. Suppl. 314, 1–65. 6595937

[B10] Baba Y. Ghetti B. Baker M. C. Uitti R. J. Hutton M. L. Yamaguchi K. (2006). Hereditary diffuse leukoencephalopathy with spheroids: Clinical, pathologic and genetic studies of a new kindred. Acta Neuropathol. 111 (4), 300–311. 10.1007/s00401-006-0046-z 16523341

[B11] Back S. A. Luo N. L. Borenstein N. S. Levine J. M. Volpe J. J. Kinney H. C. (2001). Late oligodendrocyte progenitors coincide with the developmental window of vulnerability for human perinatal white matter injury. J. Neurosci. 21 (4), 1302–1312. 10.1523/JNEUROSCI.21-04-01302.2001 11160401 PMC6762224

[B12] Baldwin K. T. Tan C. X. Strader S. T. Jiang C. Savage J. T. Elorza-Vidal X. (2021). HepaCAM controls astrocyte self-organization and coupling. Neuron 109 (15), 2427–2442. 10.1016/j.neuron.2021.05.025 34171291 PMC8547372

[B13] Barkovich A. J. Kjos B. O. Jackson D. E. Jr Norman D. (1988). Normal maturation of the neonatal and infant brain: MR imaging at 1.5 T. Radiology 166 (1 Pt 1), 173–180. 10.1148/radiology.166.1.3336675 3336675

[B14] Barth P. G. Hoffmann G. F. Jaeken J. Wanders R. J. Duran M. Jansen G. A. (1993). L-2-hydroxyglutaric acidaemia: Clinical and biochemical findings in 12 patients and preliminary report on L-2-hydroxyacid dehydrogenase. J. Inherit. Metab. Dis. 16 (4), 753–761. 10.1007/BF00711907 8412018

[B15] Baskin J. M. Wu X. Christiano R. Oh M. S. Schauder C. M. Gazzerro E. (2016). The leukodystrophy protein FAM126A (hyccin) regulates PtdIns(4)P synthesis at the plasma membrane. Nat. Cell. Biol. 18 (1), 132–138. 10.1038/ncb3271 26571211 PMC4689616

[B16] Battaglia R. A. Beltran A. S. Delic S. Dumitru R. Robinson J. A. Kabiraj P. (2019). Site-specific phosphorylation and caspase cleavage of GFAP are new markers of Alexander disease severity. Elife 8, e47789. 10.7554/eLife.47789 31682229 PMC6927689

[B17] Batzios S. P. Zafeiriou D. I. (2012). Developing treatment options for metachromatic leukodystrophy. Mol. Genet. Metab. 105 (1), 56–63. 10.1016/j.ymgme.2011.10.002 22078456

[B18] Båvner A. Shafaati M. Hansson M. Olin M. Shpitzen S. Meiner V. (2010). On the mechanism of accumulation of cholestanol in the brain of mice with a disruption of sterol 27-hydroxylase. J. Lipid Res. 51 (9), 2722–2730. 10.1194/jlr.M008326 20511491 PMC2918454

[B19] Beltran-Quintero M. L. Bascou N. A. Poe M. D. Wenger D. A. Saavedra-Matiz C. A. Nichols M. J. (2019). Early progression of Krabbe disease in patients with symptom onset between 0 and 5 months. Orphanet J. Rare Dis. 14 (1), 46. 10.1186/s13023-019-1018-4 30777126 PMC6378723

[B20] Benjamin E. R. Flanagan J. J. Schilling A. Chang H. H. Agarwal L. Katz E. (2009). The pharmacological chaperone 1-deoxygalactonojirimycin increases alpha-galactosidase A levels in Fabry patient cell lines. J. Inherit. Metab. Dis. 32 (3), 424–440. 10.1007/s10545-009-1077-0 19387866

[B21] Bennett F. C. Bennett M. L. Yaqoob F. Mulinyawe S. B. Grant G. A. Hayden Gephart M. (2018). A combination of ontogeny and CNS environment establishes microglial identity. Neuron 98 (6), 1170–1183. 10.1016/j.neuron.2018.05.014 29861285 PMC6023731

[B22] Berger J. Forss-Petter S. Eichler F. S. (2014). Pathophysiology of X-linked adrenoleukodystrophy. Biochimie 98 (100), 135–142. 10.1016/j.biochi.2013.11.023 24316281 PMC3988840

[B23] Berginer V. M. Salen G. Shefer S. (1984). Long-term treatment of cerebrotendinous xanthomatosis with chenodeoxycholic acid. N. Engl. J. Med. 311 (26), 1649–1652. 10.1056/NEJM198412273112601 6504105

[B24] Bernard G. Chouery E. Putorti M. L. Tétreault M. Takanohashi A. Carosso G. (2011). Mutations of POLR3A encoding a catalytic subunit of RNA polymerase Pol III cause a recessive hypomyelinating leukodystrophy. Am. J. Hum. Genet. 89 (3), 415–423. 10.1016/j.ajhg.2011.07.014 21855841 PMC3169829

[B25] Bernard G. Vanderver A. (2017). “POLR3-Related leukodystrophy,” in GeneReviews® [internet], ed. Adam M. P. Ardinger H. H. Pagon R. A. Wallace S. E. Bean L. J. H. Gripp K. W. (Seattle, WA: University of Washington, Seattle). 22855961

[B26] Biancheri R. Zara F. Rossi A. Mathot M. Nassogne M. C. Yalcinkaya C. (2011). Hypomyelination and congenital cataract: Broadening the clinical phenotype. Arch. Neurol. 68 (9), 1191–1194. 10.1001/archneurol.2011.201 21911699

[B27] Bidchol A. M. Dalal A. Trivedi R. Shukla A. Nampoothiri S. Sankar V. H. (2015). Recurrent and novel GLB1 mutations in India. Gene 567 (2), 173–181. 10.1016/j.gene.2015.04.078 25936995

[B28] Bieganski T. Dawydzik B. Kozlowski K. (1999). Spondylo-epimetaphyseal dysplasia: A new X-linked variant with mental retardation. Eur. J. Pediatr. 158 (10), 809–814. 10.1007/s004310051211 10486082

[B29] Biffi A. Montini E. Lorioli L. Cesani M. Fumagalli F. Plati T. (2013). Lentiviral hematopoietic stem cell gene therapy benefits metachromatic leukodystrophy. Science 341 (6148), 1233158. 10.1126/science.1233158 23845948

[B30] Bilir B. Yapici Z. Yalcinkaya C. Baris I. Carvalho C. M. Bartnik M. (2013). High frequency of GJA12/GJC2 mutations in Turkish patients with Pelizaeus-Merzbacher disease. Clin. Genet. 83 (1), 66–72. 10.1111/j.1399-0004.2012.01846.x 22283455 PMC3381985

[B31] Biundo F. Chitu V. Shlager G. G. L. Park E. S. Gulinello M. E. Saha K. (2021). Microglial reduction of colony stimulating factor-1 receptor expression is sufficient to confer adult onset leukodystrophy. Glia 69 (3), 779–791. 10.1002/glia.23929 33079443 PMC8575656

[B32] Bogorad A. M. Lin K. Y. Marintchev A. (2018). eIF2B mechanisms of action and regulation: A thermodynamic view. Biochemistry 57 (9), 1426–1435. 10.1021/acs.biochem.7b00957 29425030 PMC5840040

[B33] Bokhari M. R. Samanta D. Bokhari S. R. A. (2022). in Canavan Disease. StatPearls [internet] (Treasure Island, FL: StatPearls Publishing). 28613566

[B34] Bosch A. Estévez R. (2021). Megalencephalic leukoencephalopathy: Insights into pathophysiology and perspectives for therapy. Front. Cell. Neurosci. 14, 627887. 10.3389/fncel.2020.627887 33551753 PMC7862579

[B35] Bottelbergs A. Verheijden S. Hulshagen L. Gutmann D. H. Goebbels S. Nave K. A. (2010). Axonal integrity in the absence of functional peroxisomes from projection neurons and astrocytes. Glia 58 (13), 1532–1543. 10.1002/glia.21027 20578053

[B36] Bourre J. M. Jacque C. Nguyen-Legros J. Bornhofen J. H. Araoz C. A. Daudu O. (1978). Pelizaeus-merzbacher disease: Biochemical analysis of isolated myelin (electron-microscopy; protein, lipid and unsubstituted fatty acids analysis). Eur. Neurol. 17 (6), 317–326. 10.1159/000114969 744198

[B37] Bradbury A. M. Bagel J. H. Nguyen D. Lykken E. A. Pesayco Salvador J. Jiang X. (2020). Krabbe disease successfully treated via monotherapy of intrathecal gene therapy. J. Clin. Investig. 130 (9), 4906–4920. 10.1172/JCI133953 32773406 PMC7456224

[B38] Brady R. O. Gal A. E. Bradley R. M. Martensson E. Warshaw A. L. Laster L. (1967). Enzymatic defect in Fabry's disease. Ceramidetrihexosidase deficiency. N. Engl. J. Med. 276 (21), 1163–1167. 10.1056/NEJM196705252762101 6023233

[B39] Brazelton T. R. Rossi F. M. Keshet G. I. Blau H. M. (2000). From marrow to brain: Expression of neuronal phenotypes in adult mice. Science 290 (5497), 1775-1779. 10.1126/science.290.5497.1775 11099418

[B40] Britsch S. Goerich D. E. Riethmacher D. Peirano R. I. Rossner M. Nave K. A. (2001). The transcription factor Sox10 is a key regulator of peripheral glial development. Genes. Dev. 15 (1), 66–78. 10.1101/gad.186601 11156606 PMC312607

[B41] Brodsky M. C. Hunter J. S. (2011). Positional ocular flutter and thickened optic nerves as sentinel signs of Krabbe disease. J. AAPOS 15 (6), 595–597. 10.1016/j.jaapos.2011.05.024 22153408

[B42] Brück W. Herms J. Brockmann K. Schulz-Schaeffer W. Hanefeld F. (2001). Myelinopathia centralis diffusa (vanishing white matter disease): Evidence of apoptotic oligodendrocyte degeneration in early lesion development. Ann. Neurol. 50 (4), 532–536. 10.1002/ana.1227 11601505

[B43] Brussino A. Vaula G. Cagnoli C. Mauro A. Pradotto L. Daniele D. (2009). A novel family with Lamin B1 duplication associated with adult-onset leucoencephalopathy. J. Neurol. Neurosurg. Psychiatry 80 (2), 237–240. 10.1136/jnnp.2008.147330 19151023

[B44] Bugiani M. Dubey M. Breur M. Postma N. L. Dekker M. P. Ter Braak T. (2017). Megalencephalic leukoencephalopathy with cysts: The *glialcam*-null mouse model. Ann. Clin. Transl. Neurol. 4 (7), 450–465. 10.1002/acn3.405 28695146 PMC5497535

[B45] Byun D. S. Kasama T. Shimizu T. Yorifuji H. Seyama Y. (1988). Effect of cholestanol feeding on sterol concentrations in the serum, liver, and cerebellum of mice. J. Biochem. 103 (2), 375–379. 10.1093/oxfordjournals.jbchem.a122277 3372493

[B46] Cai J. Zhu Q. Zheng K. Li H. Qi Y. Cao Q. (2010). Co-localization of Nkx6.2 and Nkx2.2 homeodomain proteins in differentiated myelinating oligodendrocytes. Glia 58 (4), 458–68. 10.1002/glia.20937 PMC2807475 19780200

[B47] Cali J. J. Hsieh C. L. Francke U. Russell D. W. (1991). Mutations in the bile acid biosynthetic enzyme sterol 27-hydroxylase underlie cerebrotendinous xanthomatosis. J. Biol. Chem. 266 (12), 7779–7783. 10.1016/s0021-9258(20)89518-0 2019602 PMC4449724

[B48] Carson B. E. De Jesus O. (20215202). in StatPearls [internet]. Treasure island (FL) (Tampa, Florida: StatPearls Publishing).Cerebrotendinous Xanthomatosis

[B49] Carson J. H. Worboys K. Ainger K. Barbarese E. (1997). Translocation of myelin basic protein mRNA in oligodendrocytes requires microtubules and kinesin. Cell. Motil. Cytoskelet. 38 (4), 318–328. 10.1002/(SICI)1097-0169(1997)38:4<318::AID-CM2>3.0.CO;2-# 9415374

[B50] Cesani M. Lorioli L. Grossi S. Amico G. Fumagalli F. Spiga I. (2016). Mutation update of ARSA and PSAP genes causing metachromatic leukodystrophy. Hum. Mutat. 37 (1), 16–27. 10.1002/humu.22919 26462614

[B51] Chakraborty G. Mekala P. Yahya D. Wu G. Ledeen R. W. (2001). Intraneuronal N-acetylaspartate supplies acetyl groups for myelin lipid synthesis: Evidence for myelin-associated aspartoacylase. J. Neurochem. 78 (4), 736–745. 10.1046/j.1471-4159.2001.00456.x 11520894

[B52] Chang C. C. Lui C. C. Wang J. J. Huang S. H. Lu C. H. Chen C. (2010). Multi-parametric neuroimaging evaluation of cerebrotendinous xanthomatosis and its correlation with neuropsychological presentations. BMC Neurol. 10, 59. 10.1186/1471-2377-10-59 20602799 PMC2909944

[B53] Chanoumidou K. Mozafari S. Baron-Van Evercooren A. Kuhlmann T. (2020). Stem cell derived oligodendrocytes to study myelin diseases. Glia 68 (4), 705–720. 10.1002/glia.23733 31633852

[B54] Chelban V. Alsagob M. Kloth K. Chirita-Emandi A. Vandrovcova J. Maroofian R. (2020). Genetic and phenotypic characterization of NKX6-2-related spastic ataxia and hypomyelination. Eur. J. Neurol. 27 (2), 334–342. 10.1111/ene.14082 31509304 PMC6946857

[B55] Chelban V. Patel N. Vandrovcova J. Zanetti M. N. Lynch D. S. Ryten M. (2017). Mutations in NKX6-2 cause progressive spastic ataxia and hypomyelination. Am. J. Hum. Genet. 100 (6), 969–977. 10.1016/j.ajhg.2017.05.009 28575651 PMC5473715

[B56] Cheng A. Kawahata I. Fukunaga K. (2020). Fatty acid binding protein 5 mediates cell death by psychosine exposure through mitochondrial macropores formation in oligodendrocytes. Biomedicines 8 (12), 635. 10.3390/biomedicines8120635 PMC7766880 33419250

[B57] Choi J. Yang A. Song A. Lim M. Kim J. Jang J. H. (2018). Oculodentodigital dysplasia with a novel mutation in *GJA1* diagnosed by targeted gene panel sequencing: A case report and literature review. Ann. Clin. Lab. Sci. 48 (6), 776–781. 30610049

[B58] Choquet K. Forget D. Meloche E. Dicaire M. J. Bernard G. Vanderver A. (2019). Leukodystrophy-associated *POLR3A* mutations down-regulate the RNA polymerase III transcript and important regulatory RNA *BC200* . J. Biol. Chem. 294 (18), 7445–7459. 10.1074/jbc.RA118.006271 30898877 PMC6509492

[B59] Chou W. C. Guo Z. Guo H. Chen L. Zhang G. Liang K. (2021). AIM2 in regulatory T cells restrains autoimmune diseases. Nature 591 (7849), 300–305. 10.1038/s41586-021-03231-w 33505023 PMC8080937

[B60] Conant A. Curiel J. Pizzino A. Sabetrasekh P. Murphy J. Bloom M. (2018). Absence of axoglial paranodal junctions in a child with CNTNAP1 mutations, hypomyelination, and arthrogryposis. J. Child. Neurol. 33 (10), 642–650. 10.1177/0883073818776157 29882456 PMC6800098

[B61] Crow Y. J. (2013). Aicardi-Goutières syndrome. Handb. Clin. Neurol. 113, 1629–1635. 10.1016/B978-0-444-59565-2.00031-9 23622384

[B62] Crow Y. J. Leitch A. Hayward B. E. Garner A. Parmar R. Griffith E. (2006). Mutations in genes encoding ribonuclease H2 subunits cause Aicardi-Goutières syndrome and mimic congenital viral brain infection. Nat. Genet. 38 (8), 910–916. 10.1038/ng1842 16845400

[B63] Crow Y. J. Shetty J. Livingston J. H. (2020). Treatments in aicardi-goutières syndrome. Dev. Med. Child. Neurol. 62 (1), 42–47. 10.1111/dmcn.14268 31175662

[B64] Curiel J. Rodríguez Bey G. Takanohashi A. Bugiani M. Fu X. Wolf N. I. (2017). TUBB4A mutations result in specific neuronal and oligodendrocytic defects that closely match clinically distinct phenotypes. Hum. Mol. Genet. 26 (22), 4506–4518. 10.1093/hmg/ddx338 28973395 PMC7462055

[B65] da Silva C. G. Bueno A. R. Schuck P. F. Leipnitz G. Ribeiro C. A. Wannmacher C. M. (2003). L-2-hydroxyglutaric acid inhibits mitochondrial creatine kinase activity from cerebellum of developing rats. Int. J. Dev. Neurosci. 21 (4), 217–224. 10.1016/s0736-5748(03)00035-2 12781789

[B66] Dash P. K. Raj D. H. Sahu H. (2015). Megalencephalic leucoencephalopathy with subcortical cysts: Subcortical diffuse leucoencephalopathy associated with white matter cystic degeneration. BMJ Case Rep. 21, 2015, bcr2015211921. 10.1136/bcr-2015-211921 PMC4577658 26392452

[B67] de Rosa V. Secondo A. Pannaccione A. Ciccone R. Formisano L. Guida N. (2019). D-Aspartate treatment attenuates myelin damage and stimulates myelin repair. EMBO Mol. Med. 11 (1), e9278. 10.15252/emmm.201809278 30559305 PMC6328990

[B68] de Waard D. M. Bugiani M. (2020). Astrocyte-oligodendrocyte-microglia crosstalk in astrocytopathies. Front. Cell. Neurosci. 14, 608073. 10.3389/fncel.2020.608073 33328899 PMC7710860

[B69] Dooves S. Bugiani M. Postma N. L. Polder E. Land N. Horan S. T. (2016). Astrocytes are central in the pathomechanisms of vanishing white matter. J. Clin. Investig. 126 (4), 1512–1524. 10.1172/JCI83908 26974157 PMC4811153

[B70] Dorboz I. Dumay-Odelot H. Boussaid K. Bouyacoub Y. Barreau P. Samaan S. (2018). Mutation in *POLR3K* causes hypomyelinating leukodystrophy and abnormal ribosomal RNA regulation. Neurol. Genet. 4 (6), e289. 10.1212/NXG.0000000000000289 30584594 PMC6283457

[B71] Du X. Hu H. (2021). The roles of 2-hydroxyglutarate. Front. Cell. Dev. Biol. 9, 651317. 10.3389/fcell.2021.651317 33842477 PMC8033037

[B72] Duarri A. Teijido O. López-Hernández T. Scheper G. C. Barriere H. Boor I. (2008). Molecular pathogenesis of megalencephalic leukoencephalopathy with subcortical cysts: Mutations in MLC1 cause folding defects. Hum. Mol. Genet. 17 (23), 3728–3739. 10.1093/hmg/ddn269 18757878 PMC2581428

[B73] Dubey M. Bugiani M. Ridder M. C. Postma N. L. Brouwers E. Polder E. (2015). Mice with megalencephalic leukoencephalopathy with cysts: A developmental angle. Ann. Neurol. 77 (1), 114–131. 10.1002/ana.24307 25382142

[B74] Duell P. B. Salen G. Eichler F. S. DeBarber A. E. Connor S. L. Casaday L. (2018). Diagnosis, treatment, and clinical outcomes in 43 cases with cerebrotendinous xanthomatosis. J. Clin. Lipidol. 12 (5), 1169–1178. 10.1016/j.jacl.2018.06.008 30017468

[B75] Dugas J. C. Tai Y. C. Speed T. P. Ngai J. Barres B. A. (2006). Functional genomic analysis of oligodendrocyte differentiation. J. Neurosci. 26 (43), 10967–10983. 10.1523/JNEUROSCI.2572-06.2006 17065439 PMC6674672

[B76] Edgar J. M. McLaughlin M. Barrie J. A. McCulloch M. C. Garbern J. Griffiths I. R. (2004). Age-related axonal and myelin changes in the rumpshaker mutation of the Plp gene. Acta Neuropathol. 107 (4), 331–335. 10.1007/s00401-003-0808-9 14745569

[B77] Edgerley K. Barnicoat A. Offiah A. C. Calder A. D. Mankad K. Thomas N. S. (2021). AIFM1-associated X-linked spondylometaphyseal dysplasia with cerebral hypomyelination. Am. J. Med. Genet. A 185 (4), 1228–1235. 10.1002/ajmg.a.62072 33439541

[B78] Ehrlich M. Mozafari S. Glatza M. Starost L. Velychko S. Hallmann A. L. (2017). Rapid and efficient generation of oligodendrocytes from human induced pluripotent stem cells using transcription factors. Proc. Natl. Acad. Sci. U. S. A. 114 (11), E2243-E2252. 10.1073/pnas.1614412114 28246330 PMC5358375

[B79] Eichler F. Duncan C. Musolino P. L. Orchard P. J. De Oliveira S. Thrasher A. J. (2017). Hematopoietic stem-cell gene therapy for cerebral adrenoleukodystrophy. N. Engl. J. Med. 377 (17), 1630–1638. 10.1056/NEJMoa1700554 28976817 PMC5708849

[B80] Eldridge R. Anayiotos C. P. Schlesinger S. Cowen D. Bever C. Patronas N. (1984). Hereditary adult-onset leukodystrophy simulating chronic progressive multiple sclerosis. N. Engl. J. Med. 311 (15), 948–953. 10.1056/NEJM198410113111504 6472420

[B81] Elitt M. S. Barbar L. Shick H. E. Powers B. E. Maeno-Hikichi Y. Madhavan M. (2020). Suppression of proteolipid protein rescues Pelizaeus-Merzbacher disease. Nature 585 (7825), 397–403. 10.1038/s41586-020-2494-3 32610343 PMC7810164

[B82] Elorza-Vidal X. Xicoy-Espaulella E. Pla-Casillanis A. Alonso-Gardón M. Gaitán-Peñas H. Engel-Pizcueta C. (2020). Structural basis for the dominant or recessive character of GLIALCAM mutations found in leukodystrophies. Hum. Mol. Genet. 29, 1107–1120. 10.1093/hmg/ddaa009 31960914 PMC7206653

[B83] Elpidorou M. Poulter J. A. Szymanska K. Baron W. Junger K. Boldt K. (2022). Missense mutation of MAL causes a rare leukodystrophy similar to Pelizaeus-Merzbacher disease. Eur. J. Hum. Genet. 25. 10.1038/s41431-022-01050-9 PMC9259702 35217805

[B84] Eng C. M. Guffon N. Wilcox W. R. Germain D. P. Lee P. Waldek S. International Collaborative Fabry Disease Study Group (2001). Safety and efficacy of recombinant human alpha-galactosidase A replacement therapy in Fabry's disease. N. Engl. J. Med. 345 (1), 9–16. 10.1056/NEJM200107053450102 11439963

[B85] Escolar M. L. Poe M. D. Provenzale J. M. Richards K. C. Allison J. Wood S. (2005). Transplantation of umbilical-cord blood in babies with infantile Krabbe's disease. N. Engl. J. Med. 352 (20), 2069–2081. 10.1056/NEJMoa042604 15901860

[B86] Fancy S. P. Harrington E. P. Yuen T. J. Silbereis J. C. Zhao C. Baranzini S. E. (2011). Axin2 as regulatory and therapeutic target in newborn brain injury and remyelination. Nat. Neurosci. 14 (8), 1009–1016. 10.1038/nn.2855 21706018 PMC3145042

[B87] Favrais G. van de Looij Y. Fleiss B. Ramanantsoa N. Bonnin P. Stoltenburg-Didinger G. (2011). Systemic inflammation disrupts the developmental program of white matter. Ann. Neurol. 70 (4), 550–565. 10.1002/ana.22489 21796662

[B88] Fellgiebel A. Müller M. J. Mazanek M. Baron K. Beck M. Stoeter P. (2005). White matter lesion severity in male and female patients with Fabry disease. Neurology 65 (4), 600–602. 10.1212/01.wnl.0000173030.70057.eb 16116124

[B89] Feng T. Sheng R. R. Solé-Domènech S. Ullah M. Zhou X. Mendoza C. S. (2020). A role of the frontotemporal lobar degeneration risk factor TMEM106B in myelination. Brain 143 (7), 2255–2271. 10.1093/brain/awaa154 32572497 PMC7363491

[B90] Ferdinandusse S. Denis S. Mooyer P. A. Dekker C. Duran M. Soorani-Lunsing R. J. (2006a). Clinical and biochemical spectrum of D-bifunctional protein deficiency. Ann. Neurol. 59 (1), 92–104. 10.1002/ana.20702 16278854

[B91] Ferdinandusse S. Ylianttila M. S. Gloerich J. Koski M. K. Oostheim W. Waterham H. R. (2006b). Mutational spectrum of D-bifunctional protein deficiency and structure-based genotype-phenotype analysis. Am. J. Hum. Genet. 78 (1), 112–124. 10.1086/498880 16385454 PMC1380208

[B92] Ferrera D. Canale C. Marotta R. Mazzaro N. Gritti M. Mazzanti M. (2014). Lamin B1 overexpression increases nuclear rigidity in autosomal dominant leukodystrophy fibroblasts. FASEB J. 28 (9), 3906–3918. 10.1096/fj.13-247635 24858279 PMC4139899

[B93] Fields R. D. (2010). Neuroscience. Change in the brain's white matter. Science 330 (6005), 768–769. 10.1126/science.1199139 21051624 PMC3201847

[B94] Filbin M. T. Tennekoon G. I. (1993). Homophilic adhesion of the myelin P0 protein requires glycosylation of both molecules in the homophilic pair. J. Cell. Biol. 122 (2), 451–459. 10.1083/jcb.122.2.451 7686552 PMC2119647

[B95] Finnsson J. Sundblom J. Dahl N. Melberg A. Raininko R. (2015). LMNB1-related autosomal-dominant leukodystrophy: Clinical and radiological course. Ann. Neurol. 78 (3), 412–425. 10.1002/ana.24452 26053668 PMC5054845

[B96] Flechsig P. E. (1920). *Anatomie des menschlichen gehirns und rückenmarks auf myelogenetischer grundlage. V. 1*. Vol. 1. G. Thieme.

[B378] Fletcher J. L. Kondagari G. S. Vite C. H. Williamson P. Taylor R. M.. Oligodendrocyte loss during the disease course in a canine model of the lysosomal storage disease fucosidosis. J. Neuropathol. Exp. Neurol. 73 (6), 536–547. 10.1097/NEN.0000000000000075 PMC4117195 24806306

[B97] Fletcher J. L. Kondagari G. S. Wright A. L. Thomson P. C. Williamson P. Taylor R. M. (2011). Myelin genes are downregulated in canine fucosidosis. Biochim. Biophys. Acta 1812 (11), 1418–1426. 10.1016/j.bbadis.2011.06.001 21683140

[B98] Fogli A. Schiffmann R. Bertini E. Ughetto S. Combes P. Eymard-Pierre E. (2004). The effect of genotype on the natural history of eIF2B-related leukodystrophies. Neurology 62 (9), 1509–1517. 10.1212/01.wnl.0000123259.67815.db 15136673

[B99] Fogli A. Wong K. Eymard-Pierre E. Wenger J. Bouffard J. P. Goldin E. (2002). Cree leukoencephalopathy and CACH/VWM disease are allelic at the EIF2B5 locus. Ann. Neurol. 52 (4), 506–510. 10.1002/ana.10339 12325082

[B100] Forss-Petter S. Werner H. Berger J. Lassmann H. Molzer B. Schwab M. H. (1997). Targeted inactivation of the X-linked adrenoleukodystrophy gene in mice. J. Neurosci. Res. 50 (5), 829–843. 10.1002/(SICI)1097-4547(19971201)50:5<829::AID-JNR19>3.0.CO;2-W 9418970

[B101] Francis J. S. Markov V. Wojtas I. D. Gray S. McCown T. Samulski R. J. (2021). Preclinical biodistribution, tropism, and efficacy of oligotropic AAV/Olig001 in a mouse model of congenital white matter disease. Mol. Ther. Methods Clin. Dev. 20, 520–534. 10.1016/j.omtm.2021.01.009 33614826 PMC7878967

[B102] Friedman J. Smith D. E. Issa M. Y. Stanley V. Wang R. Mendes M. I. (2019). Biallelic mutations in valyl-tRNA synthetase gene VARS are associated with a progressive neurodevelopmental epileptic encephalopathy. Nat. Commun. 10 (1), 707. 10.1038/s41467-018-07067-3 30755602 PMC6372641

[B103] Fu M. M. McAlear T. S. Nguyen H. Oses-Prieto J. A. Valenzuela A. Shi R. D. (2019). The Golgi outpost protein TPPP nucleates microtubules and is critical for myelination. Cell. 179 (1), 132–146. 10.1016/j.cell.2019.08.025 31522887 PMC7214773

[B104] Gal A. (2010). “Molecular genetics of fabry disease and genotype–phenotype correlation,” in Fabry disease, ed. Elstein D. Altarescu G. Beck M. (Dordrecht: Springer). 10.1007/978-90-481-9033-1_1

[B105] Garbern J. Y. Yool D. A. Moore G. J. Wilds I. B. Faulk M. W. Klugmann M. (2002). Patients lacking the major CNS myelin protein, proteolipid protein 1, develop length-dependent axonal degeneration in the absence of demyelination and inflammation. Brain 125 (Pt 3), 551–561. 10.1093/brain/awf043 11872612

[B106] Gargareta V. I. Reuschenbach J. Siems S. B. Sun T. Piepkorn L. Mangana C. (2022). Conservation and divergence of myelin proteome and oligodendrocyte transcriptome profiles between humans and mice. Elife 11, e77019. 10.7554/eLife.77019 35543322 PMC9094742

[B107] Germain D. P. Hughes D. A. Nicholls K. Bichet D. G. Giugliani R. Wilcox W. R. (2016). Treatment of fabry's disease with the pharmacologic chaperone migalastat. N. Engl. J. Med. 375 (6), 545–555. 10.1056/NEJMoa1510198 27509102

[B108] Geva M. Cabilly Y. Assaf Y. Mindroul N. Marom L. Raini G. (2010). A mouse model for eukaryotic translation initiation factor 2B-leucodystrophy reveals abnormal development of brain white matter. Brain 133 (Pt 8), 2448–2461. 10.1093/brain/awq180 20826436

[B109] Gieselmann V. Krägeloh-Mann I. (2010). Metachromatic leukodystrophy-an update. Neuropediatrics 41 (1), 1–6. 10.1055/s-0030-1253412 20571983

[B110] Gieselmann V. (2003). Metachromatic leukodystrophy: Recent research developments. J. Child. Neurol. 18 (9), 591–594. 10.1177/08830738030180090301 14572136

[B111] Gilbert A. Elorza-Vidal X. Rancillac A. Chagnot A. Yetim M. Hingot V. (2021). Megalencephalic leukoencephalopathy with subcortical cysts is a developmental disorder of the gliovascular unit. Elife 10, e71379. 10.7554/eLife.71379 34723793 PMC8598235

[B112] Giorgio E. Pesce E. Pozzi E. Sondo E. Ferrero M. Morerio C. (2021). A high-content drug screening strategy to identify protein level modulators for genetic diseases: A proof-of-principle in autosomal dominant leukodystrophy. Hum. Mutat. 42 (1), 102–116. 10.1002/humu.24147 33252173

[B113] Goffeau A. Barrell B. G. Bussey H. Davis R. W. Dujon B. Feldmann H. (1996). Life with 6000 genes. Science 274 (5287), 563–567. 10.1126/science.274.5287.546 8849441

[B114] Groeschel S. Kühl J. S. Bley A. E. Kehrer C. Weschke B. Döring M. (2016). Long-term outcome of allogeneic hematopoietic stem cell transplantation in patients with juvenile metachromatic leukodystrophy compared with nontransplanted control patients. JAMA Neurol. 73 (9), 1133–1140. 10.1001/jamaneurol.2016.2067 27400410

[B115] Groeschel S. Vollmer B. King M. D. Connelly A. (2010). Developmental changes in cerebral grey and white matter volume from infancy to adulthood. Int. J. Dev. Neurosci. 28 (6), 481–489. 10.1016/j.ijdevneu.2010.06.004 20600789

[B116] Guasto A. Dubail J. Aguilera-Albesa S. Paganini C. Vanhulle C. Haouari W. (2022). Biallelic variants in SLC35B2 cause a novel chondrodysplasia with hypomyelinating leukodystrophy. Brain. 24, awac110. 10.1093/brain/awac110 35325049

[B117] Hagberg B. Sourander P. Svennerholm L. (1963). Diagnosis of Krabbe's infantile leucodystrophy. J. Neurol. Neurosurg. Psychiatry 26 (3), 195–198. 10.1136/jnnp.26.3.195 13951860 PMC1074213

[B118] Hagemann T. L. Powers B. Mazur C. Kim A. Wheeler S. Hung G. (2018). Antisense suppression of glial fibrillary acidic protein as a treatment for Alexander disease. Ann. Neurol. 83 (1), 27–39. 10.1002/ana.25118 29226998 PMC5876100

[B119] Han J. Sarlus H. Wszolek Z. K. Karrenbauer V. D. Harris R. A. (2020). Microglial replacement therapy: A potential therapeutic strategy for incurable CSF1R-related leukoencephalopathy. Acta Neuropathol. Commun. 8 (1), 217. 10.1186/s40478-020-01093-3 33287883 PMC7720517

[B120] Hein S. Schönfeld P. Kahlert S. Reiser G. (2008). Toxic effects of X-linked adrenoleukodystrophy-associated, very long chain fatty acids on glial cells and neurons from rat hippocampus in culture. Hum. Mol. Genet. 17 (12), 1750–1761. 10.1093/hmg/ddn066 18344355

[B121] Heng M. Y. Lin S. T. Verret L. Huang Y. Kamiya S. Padiath Q. S. (2013). Lamin B1 mediates cell-autonomous neuropathology in a leukodystrophy mouse model. J. Clin. Investig. 123 (6), 2719–2729. 10.1172/JCI66737 23676464 PMC3668844

[B122] Herbert A. L. Fu M. M. Drerup C. M. Gray R. S. Harty B. L. Ackerman S. D. (2017). Dynein/dynactin is necessary for anterograde transport of *Mbp* mRNA in oligodendrocytes and for myelination *in vivo* . Proc. Natl. Acad. Sci. U. S. A. 114 (43), E9153-E9162. 10.1073/pnas.1711088114 29073112 PMC5664533

[B123] Herrero M. Mandelboum S. Elroy-Stein O. (2019). eIF2B mutations cause mitochondrial malfunction in oligodendrocytes. Neuromolecular Med. 21 (3), 303–313. 10.1007/s12017-019-08551-9 31134486

[B124] Hess B. Saftig P. Hartmann D. Coenen R. Lüllmann-Rauch R. Goebel H. H. (1996). Phenotype of arylsulfatase A-deficient mice: Relationship to human metachromatic leukodystrophy. Proc. Natl. Acad. Sci. U. S. A. 93 (25), 14821–14826. 10.1073/pnas.93.25.14821 8962139 PMC26220

[B125] Hilz M. J. Kolodny E. H. Brys M. Stemper B. Haendl T. Marthol H. (2004). Reduced cerebral blood flow velocity and impaired cerebral autoregulation in patients with Fabry disease. J. Neurol. 251 (5), 564–570. 10.1007/s00415-004-0364-9 15164189

[B126] Hobson G. M. Huang Z. Sperle K. Sistermans E. Rogan P. K. Garbern J. Y. (2006). Splice-site contribution in alternative splicing of PLP1 and DM20: Molecular studies in oligodendrocytes. Hum. Mutat. 27 (1), 69–77. 10.1002/humu.20276 16287154

[B127] Hoegg-Beiler M. B. Sirisi S. Orozco I. J. Ferrer I. Hohensee S. Auberson M. (2014). Disrupting MLC1 and GlialCAM and ClC-2 interactions in leukodystrophy entails glial chloride channel dysfunction. Nat. Commun. 5, 3475. 10.1038/ncomms4475 24647135

[B128] Holtschmidt H. Sandhoff K. Kwon H. Y. Harzer K. Nakano T. Suzuki K. (1991). Sulfatide activator protein. Alternative splicing that generates three mRNAs and a newly found mutation responsible for a clinical disease. J. Biol. Chem. 266 (12), 7556–7560. 10.1016/s0021-9258(20)89483-6 2019586

[B129] Hopkin R. J. Bissler J. Banikazemi M. Clarke L. Eng C. M. Germain D. P. (2008). Characterization of fabry disease in 352 pediatric patients in the fabry registry. Pediatr. Res. 64 (5), 550–555. 10.1203/PDR.0b013e318183f132 18596579

[B130] Hoshino H. Kubota M. (2014). Canavan disease: Clinical features and recent advances in research. Pediatr. Int. 56 (4), 477–483. 10.1111/ped.12422 24977939

[B131] Hughes D. A. Nicholls K. Shankar S. P. Sunder-Plassmann G. Koeller D. Nedd K. (2017). Oral pharmacological chaperone migalastat compared with enzyme replacement therapy in fabry disease: 18-month results from the randomised phase III ATTRACT study. J. Med. Genet. 54 (4), 288–296. 10.1136/jmedgenet-2016-104178 27834756 PMC5502308

[B132] Hughes E. G. Kang S. H. Fukaya M. Bergles D. E. (2013). Oligodendrocyte progenitors balance growth with self-repulsion to achieve homeostasis in the adult brain. Nat. Neurosci. 16 (6), 668–676. 10.1038/nn.3390 23624515 PMC3807738

[B133] Hulshagen L. Krysko O. Bottelbergs A. Huyghe S. Klein R. Van Veldhoven P. P. (2008). Absence of functional peroxisomes from mouse CNS causes dysmyelination and axon degeneration. J. Neurosci. 28 (15), 4015–4027. 10.1523/JNEUROSCI.4968-07.2008 18400901 PMC6670456

[B134] I Amaral A. Hadera M. G. Kotter M. Sonnewald U. (2017). Oligodendrocytes do not export NAA-derived aspartate *in vitro* . Neurochem. Res. 42 (3), 827–837. 10.1007/s11064-016-1985-y 27394419 PMC5357468

[B135] Í Dali C. Groeschel S. Moldovan M. Farah M. H. Krägeloh-Mann I. Wasilewski M. (2021). Intravenous arylsulfatase A in metachromatic leukodystrophy: A phase 1/2 study. Ann. Clin. Transl. Neurol. 8 (1), 66–80. 10.1002/acn3.51254 33332761 PMC7818087

[B136] Inoue K. Khajavi M. Ohyama T. Hirabayashi S. Wilson J. Reggin J. D. (2004). Molecular mechanism for distinct neurological phenotypes conveyed by allelic truncating mutations. Nat. Genet. 36 (4), 361–369. 10.1038/ng1322 15004559

[B137] Inoue K. Osaka H. Sugiyama N. Kawanishi C. Onishi H. Nezu A. (1996). A duplicated PLP gene causing Pelizaeus-Merzbacher disease detected by comparative multiplex PCR. Am. J. Hum. Genet. 59 (1), 32–39. 8659540 PMC1915126

[B138] Inoue K. Shilo K. Boerkoel C. F. Crowe C. Sawady J. Lupski J. R. (2002). Congenital hypomyelinating neuropathy, central dysmyelination, and waardenburg-hirschsprung disease: Phenotypes linked by SOX10 mutation. Ann. Neurol. 52 (6), 836–842. 10.1002/ana.10404 12447940

[B139] Inoue K. Tanabe Y. Lupski J. R. (1999). Myelin deficiencies in both the central and the peripheral nervous systems associated with a SOX10 mutation. Ann. Neurol. 46 (3), 313–318. 10.1002/1531-8249(199909)46:3<313::aid-ana6>3.0.co;2-7 10482261

[B140] Itoh Y. Esaki T. Cook M. Qasba P. Shimoji K. Alroy J. (2001). Local and global cerebral blood flow and glucose utilization in the alpha-galactosidase A knockout mouse model of Fabry disease. J. Neurochem. 79 (6), 1217–1224. 10.1046/j.1471-4159.2001.00669.x 11752062

[B141] Jablonska B. Scafidi J. Aguirre A. Vaccarino F. Nguyen V. Borok E. (2012). Oligodendrocyte regeneration after neonatal hypoxia requires FoxO1-mediated p27Kip1 expression. J. Neurosci. 32 (42), 14775–14793. 10.1523/JNEUROSCI.2060-12.2012 23077062 PMC3517297

[B142] Jain P. Ramesh K. Mohamed A. Kumar A. Gulati S. (2012). Teaching NeuroImages: Distinct neuroimaging features of fucosidosis. Neurology 78 (5), e33. 10.1212/WNL.0b013e3182452910 22291069

[B143] Jakovcevski I. Filipovic R. Mo Z. Rakic S. Zecevic N. (2009). Oligodendrocyte development and the onset of myelination in the human fetal brain. Front. Neuroanat. 3, 5. 10.3389/neuro.05.005.2009 19521542 PMC2694674

[B144] Joss S. K. Ghazawy S. Tomkins S. Ahmed M. Bradbury J. Sheridan E. (2008). Variable expression of neurological phenotype in autosomal recessive oculodentodigital dysplasia of two sibs and review of the literature. Eur. J. Pediatr. 167 (3), 341–345. 10.1007/s00431-007-0468-1 17476528

[B145] Kajihara R. Numakawa T. Odaka H. Yaginuma Y. Fusaki N. Okumiya T. (2020). Novel drug candidates improve ganglioside accumulation and neural dysfunction in GM1 gangliosidosis models with autophagy activation. Stem Cell. Rep. 14 (5): 909–923. 10.1016/j.stemcr.2020.03.012 PMC7220856 32302553

[B146] Kaminski D. Yaghootfam C. Matthes F. Reßing A. Gieselmann V. Matzner U. (2021). Brain cell type-specific endocytosis of arylsulfatase A identifies limitations of enzyme-based therapies for metachromatic leukodystrophy. Hum. Mol. Genet. 29 (23), 3807–3817. 10.1093/hmg/ddaa277 33367737

[B147] Karumuthil-Melethil S. Marshall M. S. Heindel C. Jakubauskas B. Bongarzone E. R. Gray S. J. (2016). Intrathecal administration of AAV/GALC vectors in 10-11-day-old twitcher mice improves survival and is enhanced by bone marrow transplant. J. Neurosci. Res. 94 (11), 1138–1151. 10.1002/jnr.23882 27638599 PMC5028109

[B148] Kassmann C. M. Lappe-Siefke C. Baes M. Brügger B. Mildner A. Werner H. B. (2007). Axonal loss and neuroinflammation caused by peroxisome-deficient oligodendrocytes. Nat. Genet. 39 (8), 969–976. 10.1038/ng2070 17643102

[B149] Kassmann C. M. (2014). Myelin peroxisomes - essential organelles for the maintenance of white matter in the nervous system. Biochimie 98, 111–118. 10.1016/j.biochi.2013.09.020 24120688

[B150] Kettwig M. Schubach M. Zimmermann F. A. Klinge L. Mayr J. A. Biskup S. (2015). From ventriculomegaly to severe muscular atrophy: Expansion of the clinical spectrum related to mutations in AIFM1. Mitochondrion 21, 12–18. 10.1016/j.mito.2015.01.001 25583628

[B151] Kevelam S. H. Taube J. R. van Spaendonk R. M. Bertini E. Sperle K. Tarnopolsky M. (2015). Altered PLP1 splicing causes hypomyelination of early myelinating structures. Ann. Clin. Transl. Neurol. 2 (6), 648–661. 10.1002/acn3.203 26125040 PMC4479525

[B152] Khan A. Barber D. L. Huang J. Rupar C. A. Rip J. W. Auray-Blais C. (2021). Lentivirus-mediated gene therapy for Fabry disease. Nat. Commun. 12 (1), 1178. 10.1038/s41467-021-21371-5 33633114 PMC7907075

[B153] Kim D. Lee Y. R. Choi T. I. Kim S. H. Kang H. C. Kim C. H. (2021). Comparative proteome research in a zebrafish model for vanishing white matter disease. Int. J. Mol. Sci. 22 (5), 2707. 10.3390/ijms22052707 33800130 PMC7962458

[B154] Kim Y. I. Bhandari S. Lee J. N. Yoo K. W. Kim S. J. Oh G. S. (2014). Developmental roles of D-bifunctional protein-A zebrafish model of peroxisome dysfunction. Mol. Cells 37 (1), 74–80. 10.14348/molcells.2014.2300 24552713 PMC3907007

[B155] King K. E. Kim S. Whitley C. B. Jarnes-Utz J. R. (2020). The juvenile gangliosidoses: A timeline of clinical change. Mol. Genet. Metab. Rep. 25, 100676. 10.1016/j.ymgmr.2020.100676 33240792 PMC7674119

[B156] Klein Z. A. Takahashi H. Ma M. Stagi M. Zhou M. Lam T. T. (2017). Loss of TMEM106B ameliorates lysosomal and frontotemporal dementia-related phenotypes in progranulin-deficient mice. Neuron 95 (2), 281–296. 10.1016/j.neuron.2017.06.026 28728022 PMC5558861

[B157] Klugmann M. Schwab M. H. Pühlhofer A. Schneider A. Zimmermann F. Griffiths I. R. (1997). Assembly of CNS myelin in the absence of proteolipid protein. Neuron 18 (1), 59–70. 10.1016/s0896-6273(01)80046-5 9010205

[B158] Kobayashi T. Shinnoh N. Kondo A. Yamada T. (1997). Adrenoleukodystrophy protein-deficient mice represent abnormality of very long chain fatty acid metabolism. Biochem. Biophys. Res. Commun. 232 (3), 631–636. 10.1006/bbrc.1997.6340 9126326

[B159] Kobayashi T. Yamanaka T. Jacobs J. M. Teixeira F. Suzuki K. (1980). The twitcher mouse: An enzymatically authentic model of human globoid cell leukodystrophy (Krabbe disease). Brain Res. 202 (2), 479–483. 10.1016/0006-8993(80)90159-6 7437911

[B160] Kölker S. Pawlak V. Ahlemeyer B. Okun J. G. Hörster F. Mayatepek E. (2002). NMDA receptor activation and respiratory chain complex V inhibition contribute to neurodegeneration in d-2-hydroxyglutaric aciduria. Eur. J. Neurosci. 16 (1), 21–28. 10.1046/j.1460-9568.2002.02055.x 12153528

[B161] Komatsuzaki S. Zielonka M. Mountford W. K. Kölker S. Hoffmann G. F. Garbade S. F. (2019). Clinical characteristics of 248 patients with Krabbe disease: Quantitative natural history modeling based on published cases. Genet. Med. 21 (10), 2208–2215. 10.1038/s41436-019-0480-7 30899093

[B162] Kondagari G. S. Fletcher J. L. Cruz R. Williamson P. Hopwood J. J. Taylor R. M. (2015). The effects of intracisternal enzyme replacement versus sham treatment on central neuropathology in preclinical canine fucosidosis. Orphanet J. Rare Dis. 10, 143. 10.1186/s13023-015-0357-z 26537923 PMC4632352

[B163] Kondagari G. S. King B. M. Thomson P. C. Williamson P. Clements P. R. Fuller M. (2011). Treatment of canine fucosidosis by intracisternal enzyme infusion. Exp. Neurol. 230 (2), 218–226. 10.1016/j.expneurol.2011.04.019 21575633

[B164] Kousseff B. G. Beratis N. G. Strauss L. Brill P. W. Rosenfield R. E. Kaplan B. (1976). Fucosidosis type 2. Pediatrics 57 (2), 205–213. 10.1542/peds.57.2.205 814528

[B165] Koyama S. Sekijima Y. Ogura M. Hori M. Matsuki K. Miida T. (2021). Cerebrotendinous xanthomatosis: Molecular pathogenesis, clinical spectrum, diagnosis, and disease-modifying treatments. J. Atheroscler. Thromb. 28 (9), 905–925. 10.5551/jat.RV17055 33967188 PMC8532057

[B166] Kranendijk M. Struys E. A. van Schaftingen E. Gibson K. M. Kanhai W. A. van der Knaap M. S. (2010). IDH2 mutations in patients with D-2-hydroxyglutaric aciduria. Science 330 ((6002), 336. 10.1126/science.1192632 20847235

[B167] Krivit W. Shapiro E. G. Peters C. Wagner J. E. Cornu G. Kurtzberg J. (1998). Hematopoietic stem-cell transplantation in globoid-cell leukodystrophy. N. Engl. J. Med. 338 (16), 1119–1126. 10.1056/NEJM199804163381605 9545360

[B168] Kumar S. Biancotti J. C. Matalon R. de Vellis J. (2009). Lack of aspartoacylase activity disrupts survival and differentiation of neural progenitors and oligodendrocytes in a mouse model of Canavan disease. J. Neurosci. Res. 87 (15), 3415–3427. 10.1002/jnr.22233 19739253

[B169] Lanciotti A. Brignone M. S. Molinari P. Visentin S. De Nuccio C. Macchia G. (2012). Megalencephalic leukoencephalopathy with subcortical cysts protein 1 functionally cooperates with the TRPV4 cation channel to activate the response of astrocytes to osmotic stress: Dysregulation by pathological mutations. Hum. Mol. Genet. 21 (10), 2166–2180. 10.1093/hmg/dds032 22328087

[B170] Latour Y. L. Yoon R. Thomas S. E. Grant C. Li C. Sena-Esteves M. (2019). Human *GLB1* knockout cerebral organoids: A model system for testing AAV9-mediated *GLB1* gene therapy for reducing GM1 ganglioside storage in GM1 gangliosidosis. Mol. Genet. Metab. Rep. 21, 100513. 10.1016/j.ymgmr.2019.100513 31534909 PMC6744524

[B171] Laukka J. J. Kamholz J. Bessert D. Skoff R. P. (2016). Novel pathologic findings in patients with Pelizaeus-Merzbacher disease. Neurosci. Lett. 627, 222–232. 10.1016/j.neulet.2016.05.028 27222925 PMC4948744

[B172] Lee W. C. Kang D. Causevic E. Herdt A. R. Eckman E. A. Eckman C. B. (2010). Molecular characterization of mutations that cause globoid cell leukodystrophy and pharmacological rescue using small molecule chemical chaperones. J. Neurosci. 30 (16), 5489–5497. 10.1523/JNEUROSCI.6383-09.2010 20410102 PMC3278277

[B173] Leegwater P. A. Yuan B. Q. van der Steen J. Mulders J. Könst A. A. Boor P. K. (2001). Mutations of MLC1 (KIAA0027), encoding a putative membrane protein, cause megalencephalic leukoencephalopathy with subcortical cysts. Am. J. Hum. Genet. 68 (4), 831–838. 10.1086/319519 11254442 PMC1275636

[B174] Lenders M. Stypmann J. Duning T. Schmitz B. Brand S. M. Brand E. (2016). Serum-mediated inhibition of enzyme replacement therapy in fabry disease. J. Am. Soc. Nephrol. 27 (1), 256–264. 10.1681/ASN.2014121226 25933799 PMC4696578

[B175] Li R. Johnson A. B. Salomons G. S. van der Knaap M. S. Rodriguez D. Boespflug-Tanguy O. (2006). Propensity for paternal inheritance of de novo mutations in Alexander disease. Hum. Genet. 119 (1-2), 137–144. 10.1007/s00439-005-0116-7 16365765

[B176] Li Y. Sands M. S. (2014). Experimental therapies in the murine model of globoid cell leukodystrophy. Pediatr. Neurol. 51 (5), 600–606. 10.1016/j.pediatrneurol.2014.08.003 25240259 PMC4252788

[B177] Lieblein-Boff J. C. McKim D. B. Shea D. T. Wei P. Deng Z. Sawicki C. (2013). Neonatal *E. coli* infection causes neuro-behavioral deficits associated with hypomyelination and neuronal sequestration of iron. J. Neurosci. 33 (41), 16334–16345. 10.1523/JNEUROSCI.0708-13.2013 24107964 PMC3792468

[B178] Lin J. P. Mironova Y. A. Shrager P. Giger R. J. (2017). LRP1 regulates peroxisome biogenesis and cholesterol homeostasis in oligodendrocytes and is required for proper CNS myelin development and repair. Elife 6, e30498. 10.7554/eLife.30498 29251594 PMC5752207

[B179] Lin N. H. Yang A. W. Chang C. H. Perng M. D. (2021). Elevated GFAP isoform expression promotes protein aggregation and compromises astrocyte function. FASEB J. 35 (5), e21614. 10.1096/fj.202100087R 33908669

[B180] Livingston J. H. Crow Y. J. (2016). Neurologic phenotypes associated with mutations in TREX1, RNASEH2A, RNASEH2B, RNASEH2C, SAMHD1, ADAR1, and IFIH1: Aicardi-goutières syndrome and beyond. Neuropediatrics 47 (6), 355–360. 10.1055/s-0036-1592307 27643693

[B181] Lo Martire V. Alvente S. Bastianini S. Berteotti C. Bombardi C. Calandra-Buonaura G. (2018). Mice overexpressing lamin B1 in oligodendrocytes recapitulate the age-dependent motor signs, but not the early autonomic cardiovascular dysfunction of autosomal-dominant leukodystrophy (ADLD). Exp. Neurol. 301 (Pt A), 1–12. 10.1016/j.expneurol.2017.12.006 PMC5809293 29262292

[B182] Loddenkemper T. Grote K. Evers S. Oelerich M. Stögbauer F. (2002). Neurological manifestations of the oculodentodigital dysplasia syndrome. J. Neurol. 249 (5), 584–595. 10.1007/s004150200068 12021949

[B183] Loeb H. Tondeur M. Jonniaux G. Mockel-Pohl S. Vamos-Hurwitz E. (1969). Biochemical and ultrastructural studies in a case of mucopolysaccharidosis "F" (fucosidosis). Helv. Paediatr. Acta 24 (5), 519–537. 4247654

[B184] Lok H. C. Kwok J. B. (2021). The role of white matter dysfunction and leukoencephalopathy/leukodystrophy genes in the aetiology of frontotemporal dementias: Implications for novel approaches to therapeutics. Int. J. Mol. Sci. 22 (5), 2541. 10.3390/ijms22052541 33802612 PMC7961524

[B185] López-Hernández T. Ridder M. C. Montolio M. Capdevila-Nortes X. Polder E. Sirisi S. (2011). Mutant GlialCAM causes megalencephalic leukoencephalopathy with subcortical cysts, benign familial macrocephaly, and macrocephaly with retardation and autism. Am. J. Hum. Genet. 88 (4), 422–432. 10.1016/j.ajhg.2011.02.009 21419380 PMC3071909

[B186] Lossos A. Elazar N. Lerer I. Schueler-Furman O. Fellig Y. Glick B. (2015). Myelin-associated glycoprotein gene mutation causes Pelizaeus-Merzbacher disease-like disorder. Brain 138 (Pt 9), 2521–2536. 10.1093/brain/awv204 26179919 PMC4643626

[B187] Lu J. F. Lawler A. M. Watkins P. A. Powers J. M. Moser A. B. Moser H. W. (1997). A mouse model for X-linked adrenoleukodystrophy. Proc. Natl. Acad. Sci. U. S. A. 94 (17), 9366–9371. 10.1073/pnas.94.17.9366 9256488 PMC23196

[B188] Lumbreras S. Ricobaraza A. Baila-Rueda L. Gonzalez-Aparicio M. Mora-Jimenez L. Uriarte I. (2021). Gene supplementation of *CYP27A1* in the liver restores bile acid metabolism in a mouse model of cerebrotendinous xanthomatosis. Mol. Ther. Methods Clin. Dev. 22, 210–221. 10.1016/j.omtm.2021.07.002 PMC8399082 34485606

[B189] Lutz S. E. Zhao Y. Gulinello M. Lee S. C. Raine C. S. Brosnan C. F. (2009). Deletion of astrocyte connexins 43 and 30 leads to a dysmyelinating phenotype and hippocampal CA1 vacuolation. J. Neurosci. 29 (24), 7743–7752. 10.1523/JNEUROSCI.0341-09.2009 19535586 PMC2737812

[B190] MacDermot K. D. Holmes A. Miners A. H. (2001). Anderson-Fabry disease: Clinical manifestations and impact of disease in a cohort of 60 obligate carrier females. J. Med. Genet. 38 (11), 769–775. 10.1136/jmg.38.11.769 11732485 PMC1734754

[B191] MacDermot K. D. Holmes A. Miners A. H. (2001). Anderson-Fabry disease: Clinical manifestations and impact of disease in a cohort of 98 hemizygous males. J. Med. Genet. 38 (11), 750–760. 10.1136/jmg.38.11.750 11694547 PMC1734761

[B192] Maelfait J. Liverpool L. Rehwinkel J. (2020). Nucleic acid sensors and programmed cell death. J. Mol. Biol. 432 (2), 552–568. 10.1016/j.jmb.2019.11.016 31786265 PMC7322524

[B193] Marelli A. J. Mackie A. S. Ionescu-Ittu R. Rahme E. Pilote L. (2007). Congenital heart disease in the general population: Changing prevalence and age distribution. Circulation 115 (2), 163–172. 10.1161/CIRCULATIONAHA.106.627224 17210844

[B194] Martinez M. (2001). Restoring the DHA levels in the brains of Zellweger patients. J. Mol. Neurosci. 16 (2-3), 309–316. 10.1385/JMN:16:2-3:309 11478386

[B195] McNab F. Mayer-Barber K. Sher A. Wack A. O'Garra A. (2015). Type I interferons in infectious disease. Nat. Rev. Immunol. 15 (2), 87–103. 10.1038/nri3787 25614319 PMC7162685

[B196] Mendes M. I. Green L. M. C. Bertini E. Tonduti D. Aiello C. Smith D. (2020). RARS1-related hypomyelinating leukodystrophy: Expanding the spectrum. Ann. Clin. Transl. Neurol. 7 (1), 83–93. 10.1002/acn3.50960 31814314 PMC6952319

[B197] Mendes M. I. Gutierrez Salazar M. Guerrero K. Thiffault I. Salomons G. S. Gauquelin L. (2018). Bi-Allelic mutations in EPRS, encoding the glutamyl-prolyl-aminoacyl-tRNA synthetase, cause a hypomyelinating leukodystrophy. Am. J. Hum. Genet. 102 (4), 676–684. 10.1016/j.ajhg.2018.02.011 29576217 PMC5985283

[B198] Merheb E. Cui M. H. DuBois J. C. Branch C. A. Gulinello M. Shafit-Zagardo B. (2021). Defective myelination in an RNA polymerase III mutant leukodystrophic mouse. Proc. Natl. Acad. Sci. U. S. A. 118 (40), e2024378118. 10.1073/pnas.2024378118 34583988 PMC8501794

[B199] Meschkat M. Steyer A. M. Weil M. T. Kusch K. Jahn O. Piepkorn L. (2022). White matter integrity in mice requires continuous myelin synthesis at the inner tongue. Nat. Commun. 13 (1), 1163. 10.1038/s41467-022-28720-y 35246535 PMC8897471

[B200] Meservey L. M. Topkar V. V. Fu M. M. (2021). mRNA transport and local translation in glia. Trends Cell. Biol. 31 (6), 419–423. 10.1016/j.tcb.2021.03.006 33840591 PMC8328280

[B201] Messing A. Head M. W. Galles K. Galbreath E. J. Goldman J. E. Brenner M. (1998). Fatal encephalopathy with astrocyte inclusions in GFAP transgenic mice. Am. J. Pathol. 152 (2), 391–398. 9466565 PMC1857948

[B202] Mezey E. Chandross K. J. Harta G. Maki R. A. McKercher S. R. (2000). Turning blood into brain: Cells bearing neuronal antigens generated *in vivo* from bone marrow. Science 290 (5497), 1779–1782. 10.1126/science.290.5497.1779 11099419

[B203] Miano M. Lanino E. Gatti R. Morreale G. Fondelli P. Celle M. E. (2001). Four year follow-up of a case of fucosidosis treated with unrelated donor bone marrow transplantation. Bone Marrow Transpl. 27 (7), 747–751. 10.1038/sj.bmt.1702994 11360116

[B204] Mierzewska H. Rydzanicz M. Biegański T. Kosinska J. Mierzewska-Schmidt M. Ługowska A. (2017). Spondyloepimetaphyseal dysplasia with neurodegeneration associated with AIFM1 mutation - a novel phenotype of the mitochondrial disease. Clin. Genet. 91 (1), 30–37. 10.1111/cge.12792 27102849

[B205] Mimault C. Giraud G. Courtois V. Cailloux F. Boire J. Y. Dastugue B. (1999). Proteolipoprotein gene analysis in 82 patients with sporadic pelizaeus-merzbacher disease: Duplications, the major cause of the disease, originate more frequently in male germ cells, but point mutations do not. The clinical European network on brain dysmyelinating disease. Am. J. Hum. Genet. 65 (2), 360–369. 10.1086/302483 10417279 PMC1377935

[B206] Minchenberg S. B. Massa P. T. (2019). The control of oligodendrocyte bioenergetics by interferon-gamma (IFN-γ) and Src homology region 2 domain-containing phosphatase-1 (SHP-1). J. Neuroimmunol. 331, 46–57. 10.1016/j.jneuroim.2017.10.015 29113698 PMC5924629

[B207] Miyake N. Wolf N. I. Cayami F. K. Crawford J. Bley A. Bulas D. (2017). X-linked hypomyelination with spondylometaphyseal dysplasia (H-SMD) associated with mutations in AIFM1. Neurogenetics 18 (4), 185–194. 10.1007/s10048-017-0520-x 28842795 PMC5705759

[B208] Molander-Melin M. Pernber Z. Franken S. Gieselmann V. Månsson J. E. Fredman P. (2004). Accumulation of sulfatide in neuronal and glial cells of arylsulfatase A deficient mice. J. Neurocytol. 33 (4), 417–427. 10.1023/B:NEUR.0000046572.53905.2c 15520527

[B209] Moore D. F. Altarescu G. Barker W. C. Patronas N. J. Herscovitch P. Schiffmann R. (2003). White matter lesions in Fabry disease occur in 'prior' selectively hypometabolic and hyperperfused brain regions. Brain Res. Bull. 62 (3), 231–240. 10.1016/j.brainresbull.2003.09.021 14698356

[B210] Morrone A. Bardelli T. Donati M. A. Giorgi M. Di Rocco M. Gatti R. (2000). beta-galactosidase gene mutations affecting the lysosomal enzyme and the elastin-binding protein in GM1-gangliosidosis patients with cardiac involvement. Hum. Mutat. 15 (4), 354–366 10.1002/(SICI)1098-1004(200004)15:4<354::AID-HUMU8>3.0.CO;2-L 10737981

[B211] Morse L. E. Rosman N. P. (2006). Myoclonic seizures in Krabbe disease: A unique presentation in late-onset type. Pediatr. Neurol. 35 (2), 154–157. 10.1016/j.pediatrneurol.2006.02.004 16876017

[B212] Moser A. B. Kreiter N. Bezman L. Lu S. Raymond G. V. Naidu S. (1999). Plasma very long chain fatty acids in 3, 000 peroxisome disease patients and 29, 000 controls. Ann. Neurol. 45 (1), 100–110. 10.1002/1531-8249(199901)45:1<100::aid-art16>3.0.co;2-u 9894883

[B213] Nagy J. I. Ionescu A. V. Lynn B. D. Rash J. E. (2003). Coupling of astrocyte connexins Cx26, Cx30, Cx43 to oligodendrocyte Cx29, Cx32, Cx47: Implications from normal and connexin32 knockout mice. Glia 44 (3), 205–218. 10.1002/glia.10278 14603462 PMC1852517

[B214] Nave K. A. Lai C. Bloom F. E. Milner R. J. (1987). Splice site selection in the proteolipid protein (PLP) gene transcript and primary structure of the DM-20 protein of central nervous system myelin. Proc. Natl. Acad. Sci. U. S. A. 84 (16), 5665–5669. 10.1073/pnas.84.16.5665 2441390 PMC298923

[B215] Nawaz S. Kippert A. Saab A. S. Werner H. B. Lang T. Nave K. A. (2009). Phosphatidylinositol 4,5-bisphosphate-dependent interaction of myelin basic protein with the plasma membrane in oligodendroglial cells and its rapid perturbation by elevated calcium. J. Neurosci. 29 (15), 4794–4807. 10.1523/JNEUROSCI.3955-08.2009 19369548 PMC6665343

[B216] Nestrasil I. Ahmed A. Utz J. M. Rudser K. Whitley C. B. Jarnes-Utz J. R. (2018). Distinct progression patterns of brain disease in infantile and juvenile gangliosidoses: Volumetric quantitative MRI study. Mol. Genet. Metab. 123 (2), 97–104. 10.1016/j.ymgme.2017.12.432 29352662 PMC5832355

[B217] Ng N. Cabral-da-Silva M. C. Maksour S. Berg T. Engel M. Silva D. M. (2020). Identification of repurposable cytoprotective drugs in vanishing white matter disease patient-derived cells. Transl. Med. Commun. 21, 18. 10.1186/s41231-020-00071-0

[B218] Nguyen H. Meservey L. M. Ishiko-Silveria N. Zhou M. Huang T. T. Fu M. M. (2020). Fear deficits in hypomyelinated tppp knock-out mice. eNeuro 7 (5), ENEURO.0170–20.2020. 10.1523/ENEURO.0170-20.2020 PMC7540923 32878961

[B219] Nobuta H. Yang N. Ng Y. H. Marro S. G. Sabeur K. Chavali M. (2019). Oligodendrocyte death in pelizaeus-merzbacher disease is rescued by iron chelation. Cell. Stem Cell. 25 (4), 531–541. 10.1016/j.stem.2019.09.003 31585094 PMC8282124

[B220] Norton W. T. Poduslo S. E. (1973). Myelination in rat brain: Changes in myelin composition during brain maturation. J. Neurochem. 21 (4), 759–773. 10.1111/j.1471-4159.1973.tb07520.x 4754856

[B221] Nota B. Struys E. A. Pop A. Jansen E. E. Fernandez Ojeda M. R. Kanhai W. A. (2013). Deficiency in SLC25A1, encoding the mitochondrial citrate carrier, causes combined D-2- and L-2-hydroxyglutaric aciduria. Am. J. Hum. Genet. 92 (4), 627–631. 10.1016/j.ajhg.2013.03.009 23561848 PMC3617390

[B222] Numasawa-Kuroiwa Y. Okada Y. Shibata S. Kishi N. Akamatsu W. Shoji M. (2014). Involvement of ER stress in dysmyelination of Pelizaeus-Merzbacher Disease with PLP1 missense mutations shown by iPSC-derived oligodendrocytes. Stem Cell. Rep. 24 (51), 648–661. 10.1016/j.stemcr.2014.03.007 PMC4050482 24936452

[B223] O'Brien J. S. (1965). Stability of the myelin membrane. Science 147 (3662), 1099–1107. 10.1126/science.147.3662.1099 14242030

[B224] Odermatt B. Wellershaus K. Wallraff A. Seifert G. Degen J. Euwens C. (2003). Connexin 47 (Cx47)-deficient mice with enhanced green fluorescent protein reporter gene reveal predominant oligodendrocytic expression of Cx47 and display vacuolized myelin in the CNS. J. Neurosci. 23 (11), 4549–4559. 10.1523/JNEUROSCI.23-11-04549.2003 12805295 PMC6740816

[B225] Olivieri I. Cattalini M. Tonduti D. La Piana R. Uggetti C. Galli J. (2013). Dysregulation of the immune system in aicardi-goutières syndrome: Another example in a TREX1-mutated patient. Lupus 22 (10), 1064–1069. 10.1177/0961203313498800 23918923

[B226] Orsini J. J. Saavedra-Matiz C. A. Gelb M. H. Caggana M. (2016). Newborn screening for Krabbe's disease. J. Neurosci. Res. 94 (11), 1063–1075. 10.1002/jnr.23781 27638592 PMC5328187

[B227] Orthmann-Murphy J. L. Freidin M. Fischer E. Scherer S. S. Abrams C. K. (2007). Two distinct heterotypic channels mediate gap junction coupling between astrocyte and oligodendrocyte connexins. J. Neurosci. 27 (51), 13949–13957. 10.1523/JNEUROSCI.3395-07.2007 18094232 PMC6673504

[B228] Owczarek-Lipska M. Mulahasanovic L. Obermaier C. D. Hörtnagel K. Neubauer B. A. Korenke G. C. (2019). Novel mutations in the GJC2 gene associated with Pelizaeus-Merzbacher-like disease. Mol. Biol. Rep. 46 (4), 4507–4516. 10.1007/s11033-019-04906-4 31270756

[B229] Pace N. P. Benoit V. Agius D. Grima M. A. Parascandalo R. Hilbert P. (2019). Two novel GJA1 variants in oculodentodigital dysplasia. Mol. Genet. Genomic Med. 7 (9), e882. 10.1002/mgg3.882 31347275 PMC6732303

[B230] Padiath Q. S. Saigoh K. Schiffmann R. Asahara H. Yamada T. Koeppen A. (2006). Lamin B1 duplications cause autosomal dominant leukodystrophy. Nat Genet. 38 (10), 1114–1123. 10.1038/ng0207-276c 16951681

[B231] Paker A. M. Sunness J. S. Brereton N. H. Speedie L. J. Albanna L. Dharmaraj S. (2010). Docosahexaenoic acid therapy in peroxisomal diseases: Results of a double-blind, randomized trial. Neurology 75 (9), 826–830. 10.1212/WNL.0b013e3181f07061 20805528 PMC3013498

[B232] Park K. Hoff K. J. Wethekam L. Stence N. Saenz M. Moore J. K. (2021). Kinetically stabilizing mutations in beta tubulins create isotype-specific brain malformations. Front. Cell. Dev. Biol. 9, 765992. 10.3389/fcell.2021.765992 34869359 PMC8637541

[B233] Patil S. A. Maegawa G. H. (2013). Developing therapeutic approaches for metachromatic leukodystrophy. Drug Des. devel. Ther. 7, 729–745. 10.2147/DDDT.S15467 PMC3743609 23966770

[B234] Patzig J. Erwig M. S. Tenzer S. Kusch K. Dibaj P. Möbius W. (2016). Septin/anillin filaments scaffold central nervous system myelin to accelerate nerve conduction. Elife 5, e17119. 10.7554/eLife.17119 27504968 PMC4978525

[B235] Paznekas W. A. Boyadjiev S. A. Shapiro R. E. Daniels O. Wollnik B. Keegan C. E. (2003). Connexin 43 (GJA1) mutations cause the pleiotropic phenotype of oculodentodigital dysplasia. Am. J. Hum. Genet. 72 (2), 408–418. 10.1086/346090 12457340 PMC379233

[B236] Pierre G. Setchell K. Blyth J. Preece M. A. Chakrapani A. McKiernan P. (2008). Prospective treatment of cerebrotendinous xanthomatosis with cholic acid therapy. J. Inherit. Metab. Dis. 31 (Suppl. 2), S241–S245. 10.1007/s10545-008-0815-z 19125350

[B237] Pingault V. Bondurand N. Kuhlbrodt K. Goerich D. E. Préhu M. O. Puliti A. (1998). SOX10 mutations in patients with Waardenburg-Hirschsprung disease. Nat. Genet. 18 (2), 171–173. 10.1038/ng0298-171 9462749

[B238] Poitelon Y. Kopec A. M. Belin S. (2020). Myelin fat facts: An overview of lipids and fatty acid metabolism. Cells 9 (4), 812. 10.3390/cells9040812 PMC7226731 32230947

[B239] Poll-The B. T. Gootjes J. Duran M. De Klerk J. B. Wenniger-Prick L. J. Admiraal R. J. (2004). Peroxisome biogenesis disorders with prolonged survival: Phenotypic expression in a cohort of 31 patients. Am. J. Med. Genet. A 126A (4), 333–338. 10.1002/ajmg.a.20664 15098231

[B240] Prada V. Passalacqua M. Bono M. Luzzi P. Scazzola S. Nobbio L. A. (2012). Gain of glycosylation: A new pathomechanism of myelin protein zero mutations. Ann. Neurol. 71 (3), 427–431. 10.1002/ana.22695 22451207 PMC3315062

[B241] Priller J. Flügel A. Wehner T. Boentert M. Haas C. A. Prinz M. (2001). Targeting gene-modified hematopoietic cells to the central nervous system: Use of green fluorescent protein uncovers microglial engraftment. Nat. Med. 7 (12), 1356–1361. 10.1038/nm1201-1356 11726978

[B242] Prust M. Wang J. Morizono H. Messing A. Brenner M. Gordon E. (2011). GFAP mutations, age at onset, and clinical subtypes in Alexander disease. Neurology 77 (13), 1287–1294. 10.1212/WNL.0b013e3182309f72 21917775 PMC3179649

[B243] Pujol A. Hindelang C. Callizot N. Bartsch U. Schachner M. Mandel J. L. (2002). Late onset neurological phenotype of the X-ALD gene inactivation in mice: A mouse model for adrenomyeloneuropathy. Hum. Mol. Genet. 11 (5), 499–505. 10.1093/hmg/11.5.499 11875044

[B244] Raasakka A. Kursula P. (20201820). Flexible players within the sheaths: The intrinsically disordered proteins of myelin in health and disease. Cells 9 (2), 470. 10.3390/cells9020470 PMC7072810 32085570

[B245] Rademakers R. Baker M. Nicholson A. M. Rutherford N. J. Finch N. Soto-Ortolaza A. (2011). Mutations in the colony stimulating factor 1 receptor (CSF1R) gene cause hereditary diffuse leukoencephalopathy with spheroids. Nat. Genet. 44 (2), 200–205. 10.1038/ng.1027 22197934 PMC3267847

[B246] Rafi M. A. Luzi P. Wenger D. A. (2021). Can early treatment of twitcher mice with high dose AAVrh10-GALC eliminate the need for BMT? Bioimpacts 11 (2), 135–146. 10.34172/bi.2021.21 33842284 PMC8022232

[B247] Raskind W. H. Williams C. A. Hudson L. D. Bird T. D. (1991). Complete deletion of the proteolipid protein gene (PLP) in a family with X-linked Pelizaeus-Merzbacher disease. Am. J. Hum. Genet. 49 (6), 1355–1360. 1720927 PMC1686465

[B248] Ratti S. Rusciano I. Mongiorgi S. Owusu Obeng E. Cappellini A. Teti G. (2021). Cell signaling pathways in autosomal-dominant leukodystrophy (ADLD): The intriguing role of the astrocytes. Cell. Mol. Life Sci. 78 (6), 2781–2795. 10.1007/s00018-020-03661-1 33034697 PMC8004488

[B249] Regier D. S. Tifft C. J. Rothermel C. E. (20131993–2022). “ *GLB1-*Related disorders,” in GeneReviews® [internet], ed. Adam M. P. Ardinger H. H. Pagon R. A. Wallace S. E. Bean L. J. H. Gripp K. W. (Seattle, WA: University of Washington, Seattle). 24156116

[B250] Ribeiro R. T. Seminotti B. Zanatta Â. de Oliveira F. H. Amaral A. U. Leipnitz G. (2021). Neuronal death, glial reactivity, microglia activation, oxidative stress and bioenergetics impairment caused by intracerebroventricular administration of D-2-hydroxyglutaric acid to neonatal rats. Neuroscience 471, 115–132. 10.1016/j.neuroscience.2021.07.024 34333063

[B251] Rolyan H. Tyurina Y. Y. Hernandez M. Amoscato A. A. Sparvero L. J. Nmezi B. C. (2015). Defects of lipid synthesis are linked to the age-dependent demyelination caused by lamin B1 overexpression. J. Neurosci. 35 (34), 12002–12017. 10.1523/JNEUROSCI.1668-15.2015 26311780 PMC4549407

[B252] Rombach S. M. Smid B. E. Bouwman M. G. Linthorst G. E. Dijkgraaf M. G. Hollak C. E. (2013). Long term enzyme replacement therapy for fabry disease: Effectiveness on kidney, heart and brain. Orphanet J. Rare Dis. 8, 47. 10.1186/1750-1172-8-47 23531228 PMC3626869

[B253] Rosenberg J. B. Kaminsky S. M. Aubourg P. Crystal R. G. Sondhi D. (2016). Gene therapy for metachromatic leukodystrophy. J. Neurosci. Res. 94 (11), 1169–1179. 10.1002/jnr.23792 27638601 PMC5027970

[B254] Rossi M. Balint B. Millar Vernetti P. Bhatia K. P. Merello M. (2018). Genetic dystonia-ataxia syndromes: Clinical spectrum, diagnostic approach, and treatment options. Mov. Disord. Clin. Pract. 5 (4), 373–382. 10.1002/mdc3.12635 30363394 PMC6174447

[B255] Rzem R. Achouri Y. Marbaix E. Schakman O. Wiame E. Marie S. (2015). A mouse model of L-2-hydroxyglutaric aciduria, a disorder of metabolite repair. PLoS One 10 (3), e0119540. 10.1371/journal.pone.0119540 25763823 PMC4357467

[B256] Rzem R. Veiga-da-Cunha M. Noël G. Goffette S. Nassogne M. C. Tabarki B. (2004). A gene encoding a putative FAD-dependent L-2-hydroxyglutarate dehydrogenase is mutated in L-2-hydroxyglutaric aciduria. Proc. Natl. Acad. Sci. U. S. A. 101 (48), 16849–16854. 10.1073/pnas.0404840101 15548604 PMC534725

[B257] Rzem R. Vincent M. F. Van Schaftingen E. Veiga-da-Cunha M. (2007). L-2-hydroxyglutaric aciduria, a defect of metabolite repair. J. Inherit. Metab. Dis. 30 (5), 681–689. 10.1007/s10545-007-0487-0 17603759

[B258] Saito K. Shigetomi E. Yasuda R. Sato R. Nakano M. Tashiro K. (2018). Aberrant astrocyte Ca2+ signals "AxCa signals" exacerbate pathological alterations in an Alexander disease model. Glia 66 (5), 1053–1067. 10.1002/glia.23300 29383757

[B259] Saitoh M. Sakakihara Y. Mizuguchi M. Itoh M. Takashima S. Iwamori M. (2007). Increase of ceramide monohexoside and dipalmitoyl glycerophospholipids in the brain of Zellweger syndrome. Neurosci. Lett. 417 (2), 165–170. 10.1016/j.neulet.2007.01.083 17399899

[B260] Sakai N. (2009). Pathogenesis of leukodystrophy for Krabbe disease: Molecular mechanism and clinical treatment. Brain Dev. 31 (7), 485–487. 10.1016/j.braindev.2009.03.001 19332366

[B261] Salen G. Meriwether T. W. Nicolau G. (1975). Chenodeoxycholic acid inhibits increased cholesterol and cholestanol synthesis in patients with cerebrotendinous xanthomatosis. Biochem. Med. 14 (1), 57–74. 10.1016/0006-2944(75)90020-4 1212241

[B262] Samuraki M. Komai K. Hasegawa Y. Kimura M. Yamaguchi S. Terada N. (2008). A successfully treated adult patient with L-2-hydroxyglutaric aciduria. Neurology 70 (13), 1051–1052. 10.1212/01.wnl.0000287141.90944.95 18362286

[B263] Sanchez V. B. Ali S. Escobar A. Cuajungco M. P. (2019). Transmembrane 163 (TMEM163) protein effluxes zinc. Arch. Biochem. Biophys., 677, 108166. 10.1016/j.abb.2019.108166 PMC6864316 31697912

[B264] Sano R. Annunziata I. Patterson A. Moshiach S. Gomero E. Opferman J. (2009). GM1-ganglioside accumulation at the mitochondria-associated ER membranes links ER stress to Ca(2+)-dependent mitochondrial apoptosis. Mol. Cell. 36 (3), 500–511. 10.1016/j.molcel.2009.10.021 19917257 PMC2782904

[B265] Sase S. Almad A. A. Boecker C. A. Guedes-Dias P. Li J. J. Takanohashi A. (2020). *TUBB4A* mutations result in both glial and neuronal degeneration in an H-ABC leukodystrophy mouse model. Elife 9, e52986. 10.7554/eLife.52986 32463361 PMC7255805

[B266] Sase S. Takanohashi A. Vanderver A. Almad A. (2018). Astrocytes, an active player in Aicardi-Goutières syndrome. Brain Pathol. 28 (3), 399–407 10.1111/bpa.12600 PMC8028286 29740948

[B267] Sassa T. Wakashima T. Ohno Y. Kihara A. (2014). Lorenzo's oil inhibits ELOVL1 and lowers the level of sphingomyelin with a saturated very long-chain fatty acid. J. Lipid Res. 55 (3), 524–530. 10.1194/jlr.M044586 24489110 PMC3934736

[B268] Schaeren-Wiemers N. Bonnet A. Erb M. Erne B. Bartsch U. Kern F. (2004). The raft-associated protein MAL is required for maintenance of proper axon-glia interactions in the central nervous system. J. Cell. Biol. 166 (5), 731–742. 10.1083/jcb.200406092 15337780 PMC2172435

[B269] Schiffmann R. Moller J. R. Trapp B. D. Shih H. H. Farrer R. G. Katz D. A. (1994). Childhood ataxia with diffuse central nervous system hypomyelination. Ann. Neurol. 35 (3), 331–340. 10.1002/ana.410350314 8122885

[B270] Schiffmann R. van der Knaap M. S. Fogli A. Boespflug-Tanguy O. (2019). Abbink TEM, childhood ataxia with central nervous system hypomyelination/vanishing white matter. GeneReviews® [internet] ed. Adam M. P. Ardinger H. H. Pagon R. A. (Seattle, WA: University of Washington, Seattle). https://www.ncbi.nlm.nih.gov/books/NBK1258/. 20301435

[B271] Schiffmann R. Warnock D. G. Banikazemi M. Bultas J. Linthorst G. E. Packman S. (2009). Fabry disease: Progression of nephropathy, and prevalence of cardiac and cerebrovascular events before enzyme replacement therapy. Nephrol. Dial. Transpl. 24 (7), 2102–2111. 10.1093/ndt/gfp031 PMC2698092 19218538

[B272] Schmitt A. Gofferje V. Weber M. Meyer J. Mössner R. Lesch K. P. (2003). The brain-specific protein MLC1 implicated in megalencephalic leukoencephalopathy with subcortical cysts is expressed in glial cells in the murine brain. Glia 44 (3), 283–295. 10.1002/glia.10304 14603469

[B273] Schuster J. Sundblom J. Thuresson A. C. Hassin-Baer S. Klopstock T. Dichgans M. (2011). Genomic duplications mediate overexpression of lamin B1 in adult-onset autosomal dominant leukodystrophy (ADLD) with autonomic symptoms. Neurogenetics 12 (1), 65–72. 10.1007/s10048-010-0269-y 21225301

[B274] Schwartz N. B. Domowicz M. S. (2018). Proteoglycans in brain development and pathogenesis. FEBS Lett. 592 (23), 3791–3805. 10.1002/1873-3468.13026 29513405

[B275] Schweighauser M. Arseni D. Bacioglu M. Huang M. Lövestam S. Shi Y. (2022). Age-dependent formation of TMEM106B amyloid filaments in human brains. Nature 28, 310–314. 10.1038/s41586-022-04650-z PMC9095482 35344985

[B276] Segel R. Anikster Y. Zevin S. Steinberg A. Gahl W. A. Fisher D. (2011). A safety trial of high dose glyceryl triacetate for Canavan disease. Mol. Genet. Metab. 103 (3), 203–206. 10.1016/j.ymgme.2011.03.012 21474353

[B277] Serizawa S. Otsuka H. Seyama Y. Yamakawa T. (1982). Studies on the biosynthesis of cholestanol in cultured cells. J. Biochem. 92 (5), 1547–1557. 10.1093/oxfordjournals.jbchem.a134079 6818224

[B278] Sevin C. Aubourg P. Cartier N. (2007). Enzyme, cell and gene-based therapies for metachromatic leukodystrophy. J. Inherit. Metab. Dis. 30 (2), 175–183. 10.1007/s10545-007-0540-z 17347913

[B279] Sevrioukova I. F. (2016). Structure/function relations in AIFM1 variants associated with neurodegenerative disorders. J. Mol. Biol. 428 (18), 3650–3665. 10.1016/j.jmb.2016.05.004 27178839

[B280] Shapiro E. Krivit W. Lockman L. Jambaqué I. Peters C. Cowan M. (2000). Long-term effect of bone-marrow transplantation for childhood-onset cerebral X-linked adrenoleukodystrophy. Lancet 356 (9231), 713–718. 10.1016/S0140-6736(00)02629-5 11085690

[B281] Shin J. Y. Worman H. J. (2022). Molecular pathology of laminopathies. Annu. Rev. Pathol. 17, 159–180. 10.1146/annurev-pathol-042220-034240 34672689 PMC8881990

[B282] Shinya A. Takahashi M. Sato N. Nishida Y. Inaba A. Inaji M. (2021). Oculo-dento-digital dysplasia presenting as spastic paraparesis which was successfully treated by intrathecal baclofen therapy. Intern Med. 60 (14), 2301–2305. 10.2169/internalmedicine.6145-20 33612672 PMC8355389

[B283] Shukla A. Das Bhowmik A. Hebbar M. Rajagopal K. V. Girisha K. M. Gupta N. (2018). Homozygosity for a nonsense variant in AIMP2 is associated with a progressive neurodevelopmental disorder with microcephaly, seizures, and spastic quadriparesis. J. Hum. Genet. 63 (1), 19–25. 10.1038/s10038-017-0363-1 29215095

[B284] Siekierska A. Stamberger H. Deconinck T. Oprescu S. N. Partoens M. Zhang Y. C4RCD Research Group; AR working group of the EuroEPINOMICS RES Consortium (2019). Biallelic VARS variants cause developmental encephalopathy with microcephaly that is recapitulated in vars knockout zebrafish. Nat. Commun. 10 (1), 708. 10.1038/s41467-018-07953-w 30755616 PMC6372652

[B285] Simons C. Dyment D. Bent S. J. Crawford J. D'Hooghe M. Kohlschütter A. Venkateswaran S. Care4Rare Consortium (2017). A recurrent de novo mutation in TMEM106B causes hypomyelinating leukodystrophy. Brain 140 (12), 3105–3111. 10.1093/brain/awx314 29186371 PMC5841038

[B286] Simons C. Wolf N. I. McNeil N. Caldovic L. Devaney J. M. Takanohashi A. (2013). A de novo mutation in the β-tubulin gene TUBB4A results in the leukoencephalopathy hypomyelination with atrophy of the basal ganglia and cerebellum. Am. J. Hum. Genet. 92 (5), 767–773. 10.1016/j.ajhg.2013.03.018 23582646 PMC3644625

[B287] Sipione S. Monyror J. Galleguillos D. Steinberg N. Kadam V. (2020). Gangliosides in the brain: Physiology, pathophysiology and therapeutic applications. Front. Neurosci. 14, 572965. 10.3389/fnins.2020.572965 33117120 PMC7574889

[B288] Sirisi S. Folgueira M. López-Hernández T. Minieri L. Pérez-Rius C. Gaitán-Peñas H. (2014). Megalencephalic leukoencephalopathy with subcortical cysts protein 1 regulates glial surface localization of GLIALCAM from fish to humans. Hum. Mol. Genet. 23 (19), 5069–5086. 10.1093/hmg/ddu231 24824219

[B289] Sistermans E. A. de Coo R. F. De Wijs I. J. Van Oost B. A. (1998). Duplication of the proteolipid protein gene is the major cause of Pelizaeus-Merzbacher disease. Neurology 50 (6), 1749–1754. 10.1212/wnl.50.6.1749 9633722

[B290] Snaidero N. Möbius W. Czopka T. Hekking L. H. Mathisen C. Verkleij D. (2014). Myelin membrane wrapping of CNS axons by PI(3,4,5)P3-dependent polarized growth at the inner tongue. Cell. 156 (1-2), 277–290. 10.1016/j.cell.2013.11.044 24439382 PMC4862569

[B291] Snaidero N. Velte C. Myllykoski M. Raasakka A. Ignatev A. Werner H. B. (2017). Antagonistic functions of MBP and CNP establish cytosolic channels in CNS myelin. Cell. Rep. 18 (2), 314–323. 10.1016/j.celrep.2016.12.053 28076777 PMC5263235

[B292] Son M. Y. Kwak J. E. Seol B. Lee D. Y. Jeon H. Cho Y. S. (2015). A novel human model of the neurodegenerative disease GM1 gangliosidosis using induced pluripotent stem cells demonstrates inflammasome activation. J. Pathol. 237 (1), 98–110. 10.1002/path.4551 25925601

[B293] Southwood C. He C. Garbern J. Kamholz J. Arroyo E. Gow A. (2004). CNS myelin paranodes require Nkx6-2 homeoprotein transcriptional activity for normal structure. J. Neurosci. 24 (50), 11215–11225. 10.1523/JNEUROSCI.3479-04.2004 15601927 PMC6730372

[B294] Spada M. Pagliardini S. Yasuda M. Tukel T. Thiagarajan G. Sakuraba H. (2006). High incidence of later-onset fabry disease revealed by newborn screening. Am. J. Hum. Genet. 79 (1), 31–40. 10.1086/504601 16773563 PMC1474133

[B295] Stahl W. L. Sumi S. M. Swanson P. D. (1971). Subcellular distribution of cerebral cholestanol in cerebrotendinous xanthomatosis. J. Neurochem. 18 (3), 403–413. 10.1111/j.1471-4159.1971.tb11968.x 5559251

[B296] Stanley E. R. Berg K. L. Einstein D. B. Lee P. S. Pixley F. J. Wang Y. (1997). Biology and action of colony-stimulating factor-1. Mol. Reprod. Dev. 46 (1), 4–10 10.1002/(SICI)1098-2795(199701)46:1<4::AID-MRD2>3.0.CO;2-V 8981357

[B297] Steenweg M. E. Vanderver A. Blaser S. Bizzi A. de Koning T. J. Mancini G. M. (2010). Magnetic resonance imaging pattern recognition in hypomyelinating disorders. Brain 133 (10), 2971–2982. 10.1093/brain/awq257 20881161 PMC3589901

[B298] Steinberg S. J. Dodt G. Raymond G. V. Braverman N. E. Moser A. B. Moser H. W. (2006). Peroxisome biogenesis disorders. Biochim. Biophys. Acta 1763 (12), 1733–1748. 10.1016/j.bbamcr.2006.09.010 17055079

[B299] Steinberg S. J. Raymond G. V. Braverman N. E. Moser A. B. (2020). “Zellweger spectrum disorder,” in GeneReviews® [internet], ed. Adam M. P. Ardinger H. H. Pagon R. A. Wallace S. E. Bean L. J. H. Gripp K. W. (Seattle, WA: University of Washington, Seattle).

[B300] Stepien K. M. Ciara E. Jezela-Stanek A. (2020). Fucosidosis-clinical manifestation, long-term outcomes, and genetic profile-review and case series. Genes. (Basel) 11 (11), 1383. 10.3390/genes11111383 PMC7700486 33266441

[B301] Stoll B. J. Hansen N. I. Adams-Chapman I. Fanaroff A. A. Hintz S. R. Vohr B. National Institute of Child Health and Human Development Neonatal Research Network (2004). Neurodevelopmental and growth impairment among extremely low-birth-weight infants with neonatal infection. JAMA 292 (19), 2357–2365. 10.1001/jama.292.19.2357 15547163

[B302] Stolt C. C. Rehberg S. Ader M. Lommes P. Riethmacher D. Schachner M. (2002). Terminal differentiation of myelin-forming oligodendrocytes depends on the transcription factor Sox10. Genes. Dev. 16 (2), 165–170. 10.1101/gad.215802 11799060 PMC155320

[B303] Stradomska T. J. Syczewska M. Jamroz E. Pleskaczyńska A. Kruczek P. Ciara E. (2020). Serum very long-chain fatty acids (VLCFA) levels as predictive biomarkers of diseases severity and probability of survival in peroxisomal disorders. PLoS One 15 (9), e0238796. 32946460 10.1371/journal.pone.0238796 PMC7500652

[B304] Stradomska T. J. Tylki-Szymańska A. (2009). Serum very-long-chain fatty acids levels determined by gas chromatography in the diagnosis of peroxisomal disorders in Poland. Folia Neuropathol. 47 (4), 306–313. 20054782

[B305] Stroobants S. Wolf H. Callaerts-Vegh Z. Dierks T. Lübke T. D'Hooge R. (2018). Sensorimotor and neurocognitive dysfunctions parallel early telencephalic neuropathology in fucosidosis mice. Front. Behav. Neurosci. 12, 69. 10.3389/fnbeh.2018.00069 29706874 PMC5906539

[B306] Struys E. A. Salomons G. S. Achouri Y. Van Schaftingen E. Grosso S. Craigen W. J. (2005). Mutations in the D-2-hydroxyglutarate dehydrogenase gene cause D-2-hydroxyglutaric aciduria. Am. J. Hum. Genet. 76 (2), 358–360. 10.1086/427890 15609246 PMC1196381

[B307] Susin S. A. Lorenzo H. K. Zamzami N. Marzo I. Snow B. E. Brothers G. M. (1999). Molecular characterization of mitochondrial apoptosis-inducing factor. Nature 397 (6718), 441–446. 10.1038/17135 9989411

[B308] Suzuki K. Suzuki Y. (1970). Globoid cell leucodystrophy (Krabbe's disease): deficiency of galactocerebroside beta-galactosidase. Proc. Natl. Acad. Sci. U. S. A. 66 (2), 302–309. 10.1073/pnas.66.2.302 5271165 PMC283044

[B309] Sweeley C. C. Fabry's Disease Klionsky B. (1963). Fabry's Disease: Classification as a Sphingolipidosis and Partial Characterization of a Novel Glycolipid. J. Biol. Chem. 238, 3148–3150. 10.1016/s0021-9258(18)51888-3 14081947

[B310] Taft R. J. Vanderver A. Leventer R. J. Damiani S. A. Simons C. Grimmond S. M. (2013). Mutations in DARS cause hypomyelination with brain stem and spinal cord involvement and leg spasticity. Am. J. Hum. Genet. 92 (5), 774–780. 10.1016/j.ajhg.2013.04.006 23643384 PMC3644624

[B311] Takamura A. Higaki K. Kajimaki K. Otsuka S. Ninomiya H. Matsuda J. (2008). Enhanced autophagy and mitochondrial aberrations in murine G(M1)-gangliosidosis. Biochem. Biophys. Res. Commun. 367 (3), 616–622. 10.1016/j.bbrc.2007.12.187 18190792

[B312] Tantzer S. Sperle K. Kenaley K. Taube J. Hobson G. M. (2018). Morpholino Antisense Oligomers as a Potential Therapeutic Option for the Correction of Alternative Splicing in PMD, SPG2, and HEMS. Mol. Ther. Nucleic Acids 12, 420–432. 10.1016/j.omtn.2018.05.019 30195779 PMC6036941

[B313] Taube J. R. Sperle K. Banser L. Seeman P. Cavan B. C. Garbern J. Y. (2014). PMD patient mutations reveal a long-distance intronic interaction that regulates PLP1/DM20 alternative splicing. Hum. Mol. Genet. 23 (20), 5464–5478. 10.1093/hmg/ddu271 24890387 PMC4168831

[B314] Taylor R. M. Farrow B. R. Stewart G. J. (1992). Amelioration of clinical disease following bone marrow transplantation in fucosidase-deficient dogs. Am. J. Med. Genet. 42 (4), 628–632. 10.1002/ajmg.1320420439 1609845

[B315] Tessitore A. del P. Martin M. Sano R. Ma Y. Mann L. (2004). GM1-ganglioside-mediated activation of the unfolded protein response causes neuronal death in a neurodegenerative gangliosidosis. Mol. Cell. 15 (5), 753–766. 10.1016/j.molcel.2004.08.029 15350219

[B316] Tétreault M. Choquet K. Orcesi S. Tonduti D. Balottin U. Teichmann M. (2011). Recessive mutations in POLR3B, encoding the second largest subunit of Pol III, cause a rare hypomyelinating leukodystrophy. Am. J. Hum. Genet. 89 (5), 652–655. 10.1016/j.ajhg.2011.10.006 22036172 PMC3213403

[B317] Thakurela S. Garding A. Jung R. B. Müller C. Goebbels S. White R. (2016). The transcriptome of mouse central nervous system myelin. Sci. Rep. 6, 25828. 10.1038/srep25828 27173133 PMC4865983

[B318] Theda C. Moser A. B. Powers J. M. Moser H. W. (1992). Phospholipids in X-linked adrenoleukodystrophy white matter: fatty acid abnormalities before the onset of demyelination. J. Neurol. Sci. 110 (1-2), 195–204. 10.1016/0022-510x(92)90028-j 1506859

[B319] Tomassy G. S. Dershowitz L. B. Arlotta P. (2016). Diversity Matters: A Revised Guide to Myelination. Trends Cell. Biol. 26 (2), 135–147. 10.1016/j.tcb.2015.09.002 26442841 PMC4727993

[B320] Traboulsi E. I. Faris B. M. Der Kaloustian V. M. (1986). Persistent hyperplastic primary vitreous and recessive oculo-dento-osseous dysplasia. Am. J. Med. Genet. 24 (1), 95–100. 10.1002/ajmg.1320240111 3085500

[B321] Trajkovic K. Dhaunchak A. S. Goncalves J. T. Wenzel D. Schneider A. Bunt G. (2006). Neuron to glia signaling triggers myelin membrane exocytosis from endosomal storage sites. J. Cell. Biol. 172 (6), 937–948. 10.1083/jcb.200509022 16520383 PMC2063736

[B322] Turk B. R. Theda C. Fatemi A. Moser A. B. (2020). X-linked adrenoleukodystrophy: Pathology, pathophysiology, diagnostic testing, newborn screening and therapies. Int. J. Dev. Neurosci. 80 (1), 52–72. 10.1002/jdn.10003 31909500 PMC7041623

[B323] Uchino A. Nagai M. Kanazawa N. Ichinoe M. Yanagisawa N. Adachi K. (2020). An autopsy case of GM1 gangliosidosis type II in a patient who survived a long duration with artificial respiratory support. Neuropathology 40 (4), 379–388. 10.1111/neup.12651 32219895

[B324] Uhlenberg B. Schuelke M. Rüschendorf F. Ruf N. Kaindl A. M. Henneke M. (2004). Mutations in the gene encoding gap junction protein alpha 12 (connexin 46.6) cause Pelizaeus-Merzbacher-like disease. Am. J. Hum. Genet. 75 (2), 251–260. 10.1086/422763 15192806 PMC1216059

[B325] Vagli C. Fisicaro F. Vinciguerra L. Puglisi V. Rodolico M. S. Giordano A. (2020). Cerebral Hemodynamic Changes to Transcranial Doppler in Asymptomatic Patients with Fabry's Disease. Brain Sci. 10 (8), 546. 10.3390/brainsci10080546 PMC7464747 32806660

[B326] Vahsen N. Candé C. Brière J. J. Bénit P. Joza N. Larochette N. (2004). AIF deficiency compromises oxidative phosphorylation. EMBO J. 23 (23), 4679–4689. 10.1038/sj.emboj.7600461 15526035 PMC533047

[B327] Vallstedt A. Klos J. M. Ericson J. (2005). Multiple dorsoventral origins of oligodendrocyte generation in the spinal cord and hindbrain. Neuron 45 (1), 55–67. 10.1016/j.neuron.2004.12.026 15629702

[B328] Van Deerlin V. M. Sleiman P. M. Martinez-Lage M. Chen-Plotkin A. Wang L. S. Graff-Radford N. R. (2010). Common variants at 7p21 are associated with frontotemporal lobar degeneration with TDP-43 inclusions. Nat. Genet. 42 (3), 234–239. 10.1038/ng.536 20154673 PMC2828525

[B377] van der Knaap M. S. Fogli A. Boespflug-Tanguy O. Abbink T. E. M. Schiffmann R. (2003). “Childhood ataxia with central nervous system hypomyelination/vanishing white matter,” in GeneReviews® [internet], ed. Adam M. P. Ardinger H. H. Pagon R. A. Wallace S. E. Bean L. J. H. Gripp K. W. (Seattle, WA: University of Washington, Seattle). 20301435

[B329] van der Knaap M. S. Abbink T. E. M. Min R. (20031993). “Megalencephalic Leukoencephalopathy with Subcortical Cysts,” in GeneReviews® [internet]. Editors Adam M. P. Ardinger H. H. Pagon R. A. Wallace S. E. Bean L. J. H. Gripp K. W. (Seattle (WA): University of Washington, Seattle).

[B330] van der Knaap M. S. Barth P. G. Gabreëls F. J. Franzoni E. Begeer J. H. Stroink H. (1997). A new leukoencephalopathy with vanishing white matter. Neurology 48 (4), 845–855. 10.1212/wnl.48.4.845 9109866

[B331] van der Knaap M. S. Barth P. G. Stroink H. van Nieuwenhuizen O. Arts W. F. Hoogenraad F. (1995). Leukoencephalopathy with swelling and a discrepantly mild clinical course in eight children. Ann. Neurol. 37 (3), 324–334. 10.1002/ana.410370308 7695231

[B332] van der Knaap M. S. Bonkowsky J. L. Vanderver A. Schiffmann R. Krägeloh-Mann I. Bertini E. (2022). Therapy trial design in vanishing white matter: an expert consortium opinion. Neurol. Genet. 8 (2), e657. 10.1212/NXG.0000000000000657 35128050 PMC8811717

[B333] van der Knaap M. S. Boor I. Estévez R. (2012). Megalencephalic leukoencephalopathy with subcortical cysts: chronic white matter oedema due to a defect in brain ion and water homoeostasis. Lancet. Neurol. 11 (11), 973–985. 10.1016/S1474-4422(12)70192-8 23079554

[B334] van der Knaap M. S. Leegwater P. A. Könst A. A. Visser A. Naidu S. Oudejans C. B. (2002). Mutations in each of the five subunits of translation initiation factor eIF2B can cause leukoencephalopathy with vanishing white matter. Ann. Neurol. 51 (2), 264–270. 10.1002/ana.10112 11835386

[B335] van der Knaap M. S. Linnankivi T. Paetau A. Feigenbaum A. Wakusawa K. Haginoya K. (2007). Hypomyelination with atrophy of the basal ganglia and cerebellum: follow-up and pathology. Neurology 69 (2), 166–171. 10.1212/01.wnl.0000265592.74483.a6 17620549

[B336] van der Knaap M. S. Naidu S. Pouwels P. J. Bonavita S. van Coster R. Lagae L. (2002). New syndrome characterized by hypomyelination with atrophy of the basal ganglia and cerebellum. AJNR. Am. J. Neuroradiol. 23 (9), 1466–1474. 12372733 PMC7976795

[B337] van Egmond M. E. Pouwels P. J. Boelens J. J. Lindemans C. A. Barkhof F. Steenwijk M. D. (2013). Improvement of white matter changes on neuroimaging modalities after stem cell transplant in metachromatic leukodystrophy. JAMA Neurol. 70 (6), 779–782. 10.1001/jamaneurol.2013.629 23608771

[B338] van Grunsven E. G. Mooijer P. A. Aubourg P. Wanders R. J. (1999). Enoyl-CoA hydratase deficiency: identification of a new type of D-bifunctional protein deficiency. Hum. Mol. Genet. 8 (8), 1509–1516. 10.1093/hmg/8.8.1509 10400999

[B339] Van Haren K. van der Voorn J. P. Peterson D. R. van der Knaap M. S. Powers J. M. (2004). The life and death of oligodendrocytes in vanishing white matter disease. J. Neuropathol. Exp. Neurol. 63 (6), 618–630. 10.1093/jnen/63.6.618 15217090

[B340] Van Hoof F. Hers H. G. (1968). Mucopolysaccharidosis by absence of alpha-fucosidase. Lancet 1 (7553), 1198. 10.1016/s0140-6736(68)91895-3 4172303

[B341] van Rappard D. F. Boelens J. J. van Egmond M. E. Kuball J. van Hasselt P. M. Oostrom K. J. (2016). Efficacy of hematopoietic cell transplantation in metachromatic leukodystrophy: the Dutch experience. Blood 127 (24), 3098–3101. 10.1182/blood-2016-03-708479 27118454

[B342] Vanderver A. Hussey H. Schmidt J. L. Pastor W. Hoffman H. J. (2012). Relative incidence of inherited white matter disorders in childhood to acquired pediatric demyelinating disorders. Semin. Pediatr. Neurol. 19 (4), 219–223. 10.1016/j.spen.2012.10.001 23245555 PMC3797524

[B343] Vanrietvelde F. Lemmerling M. Mespreuve M. Crevits L. De Reuck J. Kunnen M. (2000). MRI of the brain in cerebrotendinous xanthomatosis (van Bogaert-Scherer-Epstein disease). Eur. Radiol. 10 (4), 576–578. 10.1007/s003300050964 10795535

[B344] Vellodi A. Cragg H. Winchester B. Young E. Young J. Downie C. J. (1995). Allogeneic bone marrow transplantation for fucosidosis. Bone Marrow Transpl. 15 (1), 153–158. 7742750

[B345] Verheijden S. Bottelbergs A. Krysko O. Krysko D. V. Beckers L. De Munter S. (2013). Peroxisomal multifunctional protein-2 deficiency causes neuroinflammation and degeneration of Purkinje cells independent of very long chain fatty acid accumulation. Neurobiol. Dis. 58, 258–269. 10.1016/j.nbd.2013.06.006 23777740

[B346] Von Figura K. Gieselmann V. Jaeken J. (2001). Metachromatic leukodystrophy. New York: McGraw-Hill, 3695–3724.The Metabolic and Molecular Bases of Inherited Disease

[B347] Vulinovic F. Krajka V. Hausrat T. J. Seibler P. Alvarez-Fischer D. Madoev H. (2018). Motor protein binding and mitochondrial transport are altered by pathogenic TUBB4A variants. Hum. Mutat. 39 (12), 1901–1915. 10.1002/humu.23913 30079973

[B348] Waldek S. Patel M. R. Banikazemi M. Lemay R. Lee P. (2009). Life expectancy and cause of death in males and females with Fabry disease: findings from the Fabry Registry. Genet. Med. 11 (11), 790–796. 10.1097/GIM.0b013e3181bb05bb 19745746

[B349] Wallraff A. Köhling R. Heinemann U. Theis M. Willecke K. Steinhäuser C. (2006). The impact of astrocytic gap junctional coupling on potassium buffering in the hippocampus. J. Neurosci. 26 (20), 5438–5447. 10.1523/JNEUROSCI.0037-06.2006 16707796 PMC6675300

[B350] Wang R. Y. Lelis A. Mirocha J. Wilcox W. R. (2007). Heterozygous Fabry women are not just carriers, but have a significant burden of disease and impaired quality of life. Genet. Med. 9 (1), 34–45. 10.1097/gim.0b013e31802d8321 17224688

[B351] Wasserstein M. P. Andriola M. Arnold G. Aron A. Duffner P. Erbe R. W. (2016). Clinical outcomes of children with abnormal newborn screening results for Krabbe disease in New York State. Genet. Med. 18 (12), 1235–1243. 10.1038/gim.2016.35 27171547

[B352] Weigel M. Wang L. Fu M. M. (2021). Microtubule organization and dynamics in oligodendrocytes, astrocytes, and microglia. Dev. Neurobiol. 81 (3), 310–320. 10.1002/dneu.22753 32324338

[B353] Weismann C. M. Ferreira J. Keeler A. M. Su Q. Qui L. Shaffer S. A. (2015). Systemic AAV9 gene transfer in adult GM1 gangliosidosis mice reduces lysosomal storage in CNS and extends lifespan. Hum. Mol. Genet. 24 (15), 4353–4364. 10.1093/hmg/ddv168 25964428 PMC4492398

[B354] Wenger D. A. Luzi P. (2020). “Krabbe Disease: Globoid Cell Leukodystrophy,” in Rosenberg’s molecular and genetic basis of neurological and psychiatric disease (Elsevier), 481–491.

[B355] Wiesinger C. Kunze M. Regelsberger G. Forss-Petter S. Berger J. (2013). Impaired very long-chain acyl-CoA β-oxidation in human X-linked adrenoleukodystrophy fibroblasts is a direct consequence of ABCD1 transporter dysfunction. J. Biol. Chem. 288 (26), 19269–19279. 10.1074/jbc.M112.445445 23671276 PMC3696697

[B356] Wilcox W. R. Linthorst G. E. Germain D. P. Feldt-Rasmussen U. Waldek S. Richards S. M. (2012). Anti-α-galactosidase A antibody response to agalsidase beta treatment: data from the Fabry Registry. Mol. Genet. Metab. 105 (3), 443–449. 10.1016/j.ymgme.2011.12.006 22227322

[B357] Wilhelmsson U. Li L. Pekna M. Berthold C. H. Blom S. Eliasson C. (2004). Absence of glial fibrillary acidic protein and vimentin prevents hypertrophy of astrocytic processes and improves post-traumatic regeneration. J. Neurosci. 24 (21), 5016–5021. 10.1523/JNEUROSCI.0820-04.2004 15163694 PMC6729371

[B358] Willems P. J. Darby J. K. DiCioccio R. A. Nakashima P. Eng C. Kretz K. A. (1988). Identification of a mutation in the structural alpha-L-fucosidase gene in fucosidosis. Am. J. Hum. Genet. 43 (5), 756–763. 2903668 PMC1715535

[B359] Willems P. J. Gatti R. Darby J. K. Romeo G. Durand P. Dumon J. E. (1991). Fucosidosis revisited: a review of 77 patients. Am. J. Med. Genet. 38 (1), 111–131. 10.1002/ajmg.1320380125 2012122

[B360] Wolf N. I. Biancheri R. Zara F. Bruno C. Gazzerro E. Rossi A. (2021). “Hypomyelination and Congenital Cataract,” in GeneReviews® [internet], ed. Adam M. P. Ardinger H. H. Pagon R. A. Wallace S. E. Bean L. J. H. Gripp K. W. (Seattle, WA: University of Washington, Seattle). 20301737

[B361] Wolf N. I. ffrench-Constant C. van der Knaap M. S. (2021). Hypomyelinating leukodystrophies - unravelling myelin biology. Nat. Rev. Neurol. 17 (2), 88–103. 10.1038/s41582-020-00432-1 33324001

[B362] Wong K. Armstrong R. C. Gyure K. A. Morrison A. L. Rodriguez D. Matalon R. (2000). Foamy cells with oligodendroglial phenotype in childhood ataxia with diffuse central nervous system hypomyelination syndrome. Acta Neuropathol. 100 (6), 635–646. 10.1007/s004010000234 11078215

[B363] Woodward L. J. Anderson P. J. Austin N. C. Howard K. Inder T. E. (2006). Neonatal MRI to predict neurodevelopmental outcomes in preterm infants. N. Engl. J. Med. 355 (7), 685–694. 10.1056/NEJMoa053792 16914704

[B364] Wright M. D. Poe M. D. DeRenzo A. Haldal S. Escolar M. L. (2017). Developmental outcomes of cord blood transplantation for Krabbe disease: A 15-year study. Neurology 89 (13), 1365–1372. 10.1212/WNL.0000000000004418 28855403 PMC5649761

[B365] Yahalom G. Tsabari R. Molshatzki N. Ephraty L. Cohen H. Hassin-Baer S. (2013). Neurological outcome in cerebrotendinous xanthomatosis treated with chenodeoxycholic acid: early versus late diagnosis. Clin. Neuropharmacol. 36 (3), 78–83. 10.1097/WNF.0b013e318288076a 23673909

[B366] Yam G. H. Zuber C. Roth J. (2005). A synthetic chaperone corrects the trafficking defect and disease phenotype in a protein misfolding disorder. FASEB J. 19 (1), 12–18. 10.1096/fj.04-2375com 15629890

[B367] Yamamoto A. Fukumura S. Habata Y. Miyamoto S. Nakashima M. Takashima S. (2021). Novel HSD17B4 Variants Cause Progressive Leukodystrophy in Childhood: Case Report and Literature Review. Child. Neurol. Open 8, 2329048X211048613. 10.1177/2329048X211048613 PMC8512218 34660840

[B368] Yan H. Helman G. Murthy S. E. Ji H. Crawford J. Kubisiak T. (2019). Heterozygous Variants in the Mechanosensitive Ion Channel TMEM63A Result in Transient Hypomyelination during Infancy. Am. J. Hum. Genet. 105 (5), 996–1004. 10.1016/j.ajhg.2019.09.011 31587869 PMC6848986

[B369] Yan H. Yang S. Hou Y. Ali S. Escobar A. Gao K. (2022). Functional Study of TMEM163 Gene Variants Associated with Hypomyelination Leukodystrophy. Cells 11 (8), 1285. 10.3390/cells11081285 35455965 PMC9031525

[B370] Yilmaz K. (2009). Riboflavin treatment in a case with l-2-hydroxyglutaric aciduria. Eur. J. Paediatr. Neurol. 13 (1), 57–60. 10.1016/j.ejpn.2008.01.003 18343698

[B371] Yoshida T. Sasaki M. Yoshida M. Namekawa M. Okamoto Y. Tsujino S. Alexander Disease Study Group in Japan (2011). Nationwide survey of Alexander disease in Japan and proposed new guidelines for diagnosis. J. Neurol. 258 (11), 1998–2008. 10.1007/s00415-011-6056-3 21533827

[B372] Zara F. Biancheri R. Bruno C. Bordo L. Assereto S. Gazzerro E. (2006). Deficiency of hyccin, a newly identified membrane protein, causes hypomyelination and congenital cataract. Nat. Genet. 38 (10), 1111–1113. 10.1038/ng1870 16951682

[B373] Zerbin-Rüdin E. Peiffer J. (1964). [Genetic contribution to the problem of the late form of Pelizaeus-Merzbacher disease]. Humangenetik 1 (2), 107–122. 10.1007/BF00389627 5869474

[B374] Zhang Y. Chen K. Sloan S. A. Bennett M. L. Scholze A. R. O'Keeffe S. (2014). An RNA-sequencing transcriptome and splicing database of glia, neurons, and vascular cells of the cerebral cortex. J Neurosci. 3435 (362), 11929–11947. 10.1523/JNEUROSCI.1860-14.2014 PMC4152602 25186741

[B375] Zhou X. Nicholson A. M. Ren Y. Brooks M. Jiang P. Zuberi A. (2020). Loss of TMEM106B leads to myelination deficits: implications for frontotemporal dementia treatment strategies. Brain 143 (6), 1905–1919. 10.1093/brain/awaa141 32504082 PMC7296855

[B376] Zuchero J. B. Fu M. M. Sloan S. A. Ibrahim A. Olson A. Zaremba A. (2015). CNS myelin wrapping is driven by actin disassembly. Dev. CellDev Cell. 34 (2), 152–167. 10.1016/j.devcel.2015.06.011 PMC4519368 26166300

